# Plasma metabolomics and quantitative interstitial abnormalities in ever-smokers

**DOI:** 10.1186/s12931-023-02576-2

**Published:** 2023-11-04

**Authors:** Bina Choi, Raúl San José Estépar, Suneeta Godbole, Jeffrey L. Curtis, Jennifer M. Wang, Rubén San José Estépar, Ivan O. Rosas, Jared R. Mayers, Brian D. Hobbs, Craig P. Hersh, Samuel Y. Ash, MeiLan K. Han, Russell P. Bowler, Kathleen A. Stringer, George R. Washko, Wassim W. Labaki

**Affiliations:** 1 Division of Pulmonary and Critical Care Medicine, Department of Medicine, Brigham and Women’s Hospital, 75 Francis Street, Pulmonary-PBB-CA-3, Boston, MA 02115 USA; 2 Applied Chest Imaging Laboratory, Brigham and Women’s Hospital, Boston, MA USA; 3 Department of Radiology, Brigham and Women’s Hospital, Boston, MA USA; 4 Anschutz Medical Campus, Department of Biostatistics and Informatics, University of Colorado, Aurora, CO USA; 5 Division of Pulmonary and Critical Care Medicine, Department of Internal Medicine, University of Michigan, Ann Arbor, MI USA; 6 Medical Service, VA Ann Arbor Healthcare System, Ann Arbor, MI USA; 7 Baylor College of Medicine, Houston, TX USA; 8 Channing Division of Network Medicine, Department of Medicine, Brigham and Women’s Hospital, Boston, MA USA; 9 Department of Critical Care, South Shore Hospital, South Weymouth, MA USA; 10 Division of Pulmonary, Critical Care and Sleep Medicine, Department of Medicine, National Jewish Health, Denver, CO USA; 11 Department of Clinical Pharmacy, College of Pharmacy, University of Michigan, Ann Arbor, MI USA

**Keywords:** Lung Diseases, Interstitial, Metabolomics, Pulmonary Emphysema, Tomography, X-Ray Computed, Cross-Sectional Studies

## Abstract

**Background:**

Quantitative interstitial abnormalities (QIA) are an automated computed tomography (CT) finding of early parenchymal lung disease, associated with worse lung function, reduced exercise capacity, increased respiratory symptoms, and death. The metabolomic perturbations associated with QIA are not well known. We sought to identify plasma metabolites associated with QIA in smokers. We also sought to identify shared and differentiating metabolomics features between QIA and emphysema, another smoking-related advanced radiographic abnormality.

**Methods:**

In 928 former and current smokers in the Genetic Epidemiology of COPD cohort, we measured QIA and emphysema using an automated local density histogram method and generated metabolite profiles from plasma samples using liquid chromatography–mass spectrometry (Metabolon). We assessed the associations between metabolite levels and QIA using multivariable linear regression models adjusted for age, sex, body mass index, smoking status, pack-years, and inhaled corticosteroid use, at a Benjamini–Hochberg False Discovery Rate p-value of ≤ 0.05. Using multinomial regression models adjusted for these covariates, we assessed the associations between metabolite levels and the following CT phenotypes: QIA-predominant, emphysema-predominant, combined-predominant, and neither- predominant. Pathway enrichment analyses were performed using MetaboAnalyst.

**Results:**

We found 85 metabolites significantly associated with QIA, with overrepresentation of the nicotinate and nicotinamide, histidine, starch and sucrose, pyrimidine, phosphatidylcholine, lysophospholipid, and sphingomyelin pathways. These included metabolites involved in inflammation and immune response, extracellular matrix remodeling, surfactant, and muscle cachexia. There were 75 metabolites significantly different between QIA-predominant and emphysema-predominant phenotypes, with overrepresentation of the phosphatidylethanolamine, nicotinate and nicotinamide, aminoacyl-tRNA, arginine, proline, alanine, aspartate, and glutamate pathways.

**Conclusions:**

Metabolomic correlates may lend insight to the biologic perturbations and pathways that underlie clinically meaningful quantitative CT measurements like QIA in smokers.

**Supplementary Information:**

The online version contains supplementary material available at 10.1186/s12931-023-02576-2.

## Background

Smokers without interstitial lung disease or emphysema may have quantitative interstitial abnormalities (QIA), which are subtle parenchymal changes on chest computed tomography (CT) scans detected by an automated method [1]. The presence and progression of QIA (also called interstitial features in prior work) are associated with worse lung function, reduced exercise capacity, increased respiratory symptoms, and death [1–4]. Risk factors for QIA include advanced age, current smoking status, the *MUC5B* polymorphism, and female sex [1–4]. Given its shared risk and clinical factors to both idiopathic pulmonary fibrosis (IPF) and chronic obstructive pulmonary disease (COPD), QIA may be a precursor to advanced parenchymal diseases in some patients [5, 6], for whom existing therapies slow the future development or decrease symptoms of disease but do not reverse the parenchymal damage [7–10]. QIA may be a useful target for early intervention, but the biomarkers associated with QIA remain unclear.

Metabolomics may be useful for understanding the biochemical perturbations associated with an early stage of lung disease like QIA. Metabolomics is the field of the identification and measurement of small molecules (≤ 1500 Daltons) in a single biological specimen [11]. Endogenous metabolites are end-products of enzymatic reactions and linked by metabolic pathways, making them downstream of genomics, transcriptomics, and proteomics; they can also be derived from food, medications, microbiota, and the environment [12, 13]. Metabolism may be perturbed in disease and can directly reflect the underlying pathogenic mechanisms [12].

Prior work has shown that serum and plasma metabolomic analyses are useful in the study of established and advanced lung diseases. Serum and plasma metabolomics have been used to discriminate healthy controls from those with COPD or IPF [14, 15] and to detect the presence and extent of radiographic emphysema and other measures of disease severity [16–18]. Systemic metabolomics may similarly reflect and provide biochemical insight into more subtle parenchymal injury like QIA.

In this study, we used a global metabolomics assay that captures a broad range of chemical classes of metabolites to identify those associated with QIA in a well-characterized cohort of ever-smokers. Additionally, given shared risk factors between QIA and quantitative emphysema, we sought to identify shared and differentiating metabolite profiles in QIA-predominant versus emphysema-predominant CT phenotypes.

## Methods

This was a cross-sectional cohort study of metabolomics features associated with QIA. The Genetic Epidemiology of COPD (COPDGene) is a prospective observational study of over 10,000 former and current smokers (ever-smokers) aged 45–80 years with at least a 10 pack-year smoking history and no prior history of bronchiectasis or interstitial lung disease (ILD) [19]. Participants self-reporting as Non-Hispanic White or Black were recruited at 21 study centers across the United States. For this study, we used data from questionnaires, chest CT scans, and blood samples collected at the five-year follow up (visit 2) of the study (2013–2017), as previously described [19]. The COPDGene study (NCT00608764) was approved by the institutional review board for ethical review at all 21 participating centers (Additional file 1). All participants provided written informed consent.

### Chest CT measurements

**Fig. 1:**
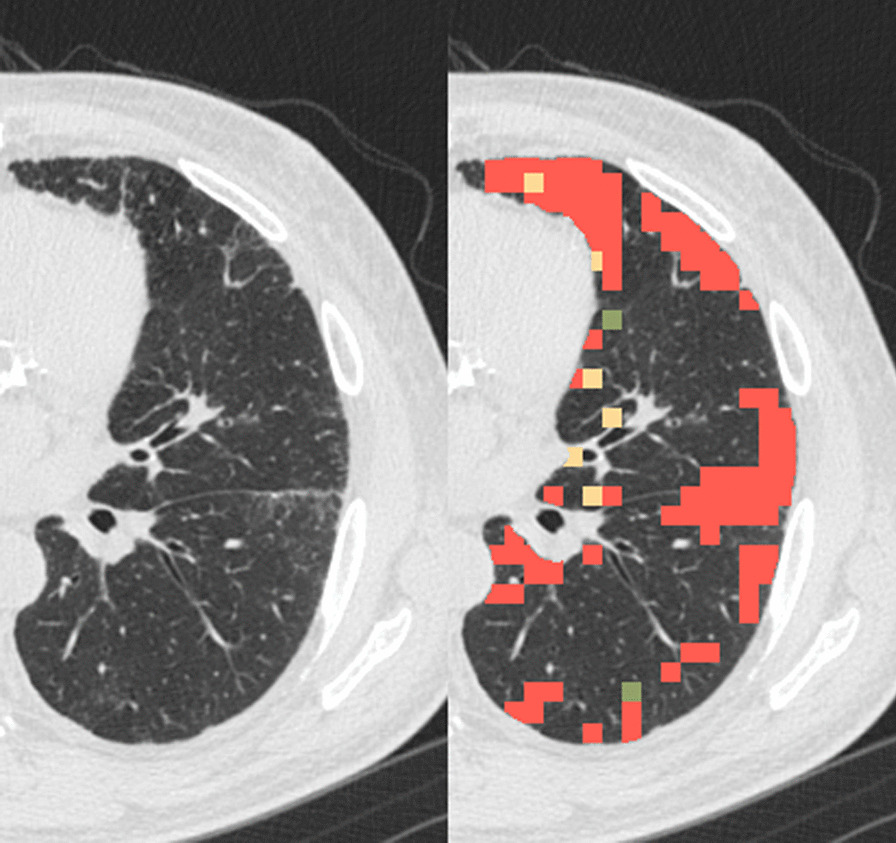
Quantitative interstitial abnormalities (QIA). The left image is an example computed tomography (CT) cut of a left lung, and the right image is the same cut with QIA shaded

We measured QIA and quantitative emphysema in 4,778 participants using a previously-published automated tool [1]. The tool employs a machine learning classifier using local density histograms and distances from the pleural surface to identify the voxels of total CT lung volume as radiologic features. The percentages of total lung volume of reticulation, subpleural lines, ground glass opacities, honeycombing, linear scarring, centrilobular nodules, and other nodularity features were summed to yield the total QIA percent (Fig. 1); panlobular and centrilobular emphysema features were summed to yield total emphysema percent. We used continuous QIA for the primary analysis.

### Plasma metabolomics measurements

**Fig. 2:**
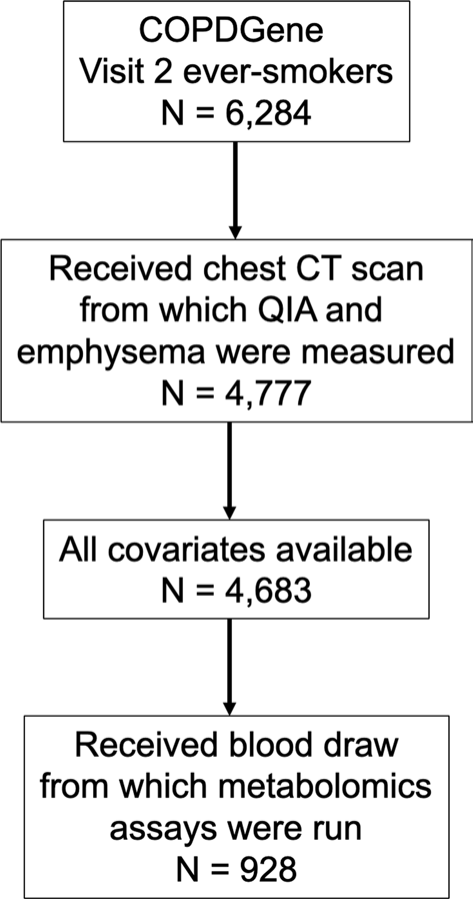
Flow diagram of participants included in the study

Metabolomics assays were run on plasma samples collected from 1,136 participants from two study centers (National Jewish Health, University of Iowa) at visit 2. This analysis included 928 participants with complete quantitative CT and metabolomics level measurements available (Fig. 2). Plasma samples were profiled using the Metabolon Global Metabolomics Platform (Morrisville, NC), as described previously and in the Additional file 1. [20–22]

Metabolite levels were median scaled within each batch. Of the 1,392 metabolites initially profiled by the Metabolon platform, 397 with > 20% missingness were excluded, as done in prior work, referred as the “80%” rule [23]. Of the remaining 995 metabolites, 237 were quantified but chemically unidentified, and they were not used in our analysis. The remaining 758 named metabolites were used in our analysis. For the 400 metabolites that had ≤ 20% missingness, missing values were imputed using k-nearest neighbor sample imputation (k = 10); 358 metabolites had no values missing. Missingness is an inherent element of metabolomics data, which routinely requires pre-processing such as data reduction and imputation [24]. All values were log2-transformed in preparation for statistical analyses. There were 363 involved in lipid, 180 in amino acid, 32 in nucleotide, 24 in carbohydrate, and 10 in energy metabolism (Additional file 1: Table S1). Additionally, there were 25 cofactors and vitamins, 25 peptides, 96 xenobiotics, and 3 partially-characterized metabolites.

### Statistical analyses

We assessed the associations between each metabolite level (individual predictor) and continuous QIA (primary outcome) with univariable linear regression then multivariable linear regression adjusted for age, sex, body mass index (BMI), smoking status, pack-years, and inhaled corticosteroid (ICS) use, using a Benjamini–Hochberg False Discovery Rate (FDR) p-value of ≤ 0.05. Models were adjusted for BMI given obesity perturbs the metabolome, and obesity-related atelectasis may contribute to CT noise [25]. For the secondary analysis, QIA and emphysema were both dichotomized by a cutoff of each measure occupying ≥ 5% or < 5% of the total lung volume; cutoffs were determined based on prior work [1, 26]. We categorized participants into four CT-based phenotypes, defining those with ≥ 5% QIA and < 5% emphysema as QIA-predominant, ≥ 5% QIA and ≥ 5% emphysema as combined-predominant, < 5% QIA and ≥ 5% emphysema as emphysema-predominant, and < 5% QIA and < 5% emphysema as neither-predominant (Additional file 1: Fig. S1). We assessed unadjusted associations between each metabolite level and the CT phenotype (secondary outcome) using analysis of variance (ANOVA), then used multinomial logistic regression adjusted for the covariates listed above, at an FDR of ≤ 0.05, with QIA-predominant used as the reference group. Analyses were performed using R software (version 4.2.2) and implemented using RStudio (version 2022.12.0 + 353). [27, 28]

We performed metabolic pathway enrichment analyses of the significant metabolites using the web platform MetaboAnalyst (V5.0) [29]. L- and D-enantiomer annotations for amino acids were simplified to the L-enantiomer. Metabolites with missing or more than one Human Metabolome Database (HMDB) ID annotations were excluded [30]. The metabolic pathways were mapped to the homo sapiens Kyoto Encyclopedia of Genes and Genomes database (KEGG) [31], then pathway enrichment analyses were performed by global test at an FDR of ≤ 0.05 and topology analyses by relative-betweenness centrality.

## Results

The baseline characteristics of the 928 participants in this analysis are shown in Table 1. The participants had mean age of 67.5 ± 8.6 years, were 50.2% male, and were predominantly former smokers. Mean percent predicted forced expiratory volume in 1 s (FEV1) was 77.8 ± 26.0%, and mean percent predicted forced vital capacity (FVC) was 86.6 ± 18.5%. The mean percentage of lung occupied by QIA was 5.0 ± 4.3% and by emphysema was 8.1 ± 16.0%. In the cohort, 223 had QIA-predominant, 109 had combined-predominant, 133 had emphysema-predominant, and 463 had neither-predominant CT phenotypes (Additional file 1: Table S2). The participants in our cohort, when compared to the rest of the COPDGene cohort with CT measurements and covariables but no metabolomics data available, had similar characteristics but were older, with a greater percentage of former smokers and predominantly White in self-reported race (Additional file 1: Table S3).

**Table 1:** Baseline characteristics

	N = 928
Age, mean ± SD	67.5 ± 8.6
Male	466 (50.2)
Self-reported race	
White	850 (91.6)
Black	78 (8.4)
Former Smoker	688 (74.1)
Pack Years, mean ± SD	44.5 ± 24.3
Body Mass Index (kg/m2), mean ± SD	29.0 ± 6.1
Inhaled corticosteroid use	49 (5.3)
Post Bronchodilator FEV1 (L), mean ± SD	2.0 ± 0.9
Post Bronchodilator FEV1 (percent predicted), mean ± SD	77.8 ± 26.0
Post Bronchodilator FVC (L), mean ± SD	3.1 ± 1.0
Post Bronchodilator FVC (percent predicted), mean ± SD	86.6 ± 18.5
GOLD class	
PRISm (reduced FEV1 and FVC, with a FEV1-to-FVC ratio of ≥ 0.7)	82 (8.8)
GOLD 0	412 (44.4)
GOLD 1	97 (10.5)
GOLD 2	198 (21.4)
GOLD 3	109 (11.8)
GOLD 4	29 (3.1)
Percentage of Lung Occupied by QIA, mean ± SD	5.0 ± 4.3
≥ 5% Percentage of Lung Occupied by QIA	332 (35.8)
Percentage of Lung Occupied by Emphysema, mean ± SD	8.1 ± 16.0
≥ 5% Percentage of Lung Occupied by Emphysema	242 (26.1)

N(%) unless otherwise specified

*FEV1* forced expiratory volume in 1s, FVC forced vital capacity, *GOLD* Global Initiative for Chronic Obstructive Lung Disease, *PRISm* Preserved ratio impaired spirometry, *QIA* quantitative interstitial abnormalities

### Association of metabolomics with QIA

**Fig. 3:**
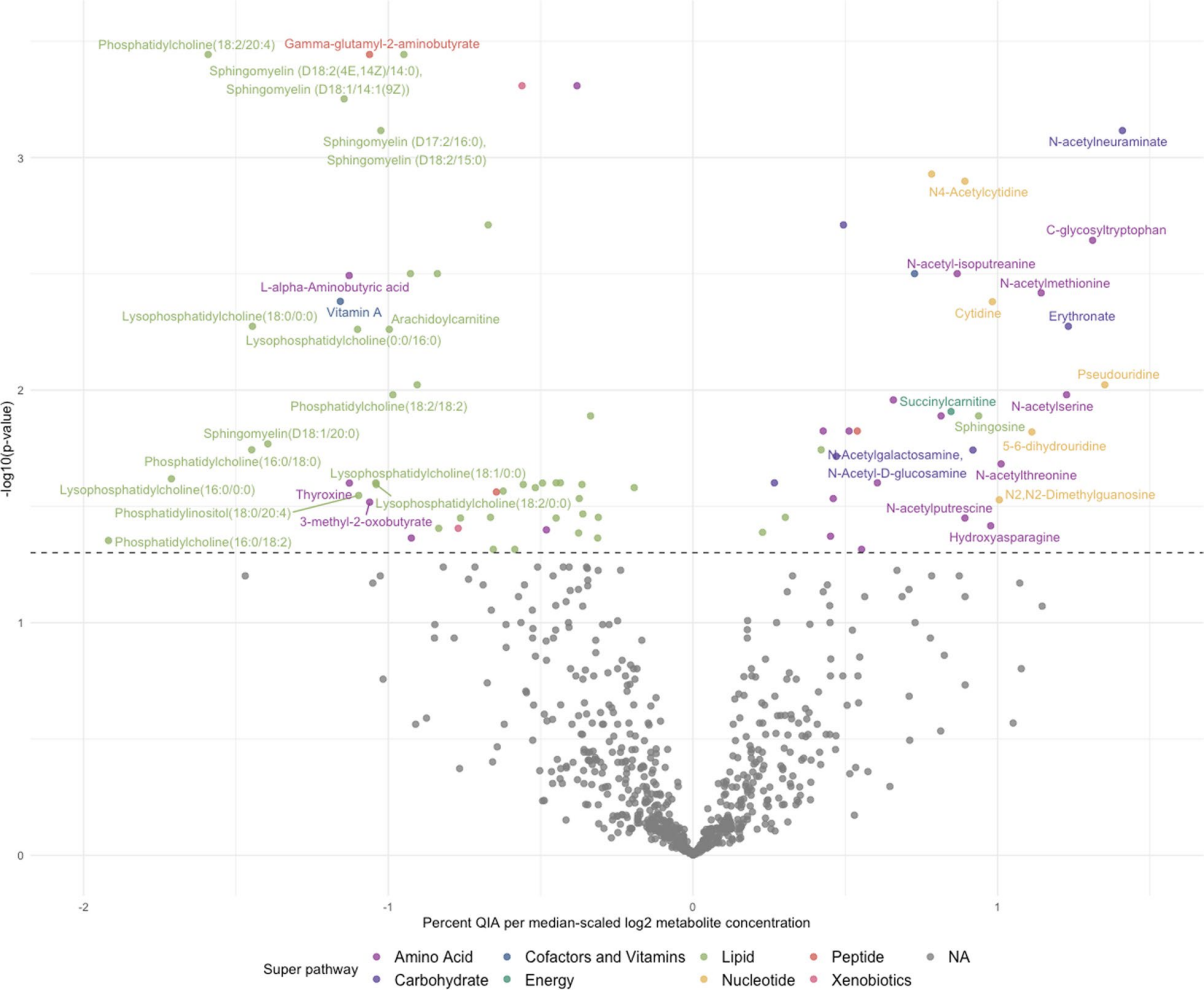
Volcano plot of median-scaled, log-transformed metabolites associated with QIA. Metabolites are colored by super pathway if significantly associated with QIA and colored in gray if insignificant (FDR ≤ 0.05)

We identified significant associations between 223 metabolites and continuous QIA by univariable linear regression (Additional file 1: Table S4). By multivariable regression, 85 metabolites were significantly associated with QIA (Fig. 3, Additional file 1: Table S5), of which 51 metabolites were negatively-associated and 34 positively-associated with QIA, including 44 (51.8%) lipids and 21 (24.7%) amino acids.

**Fig. 4:**
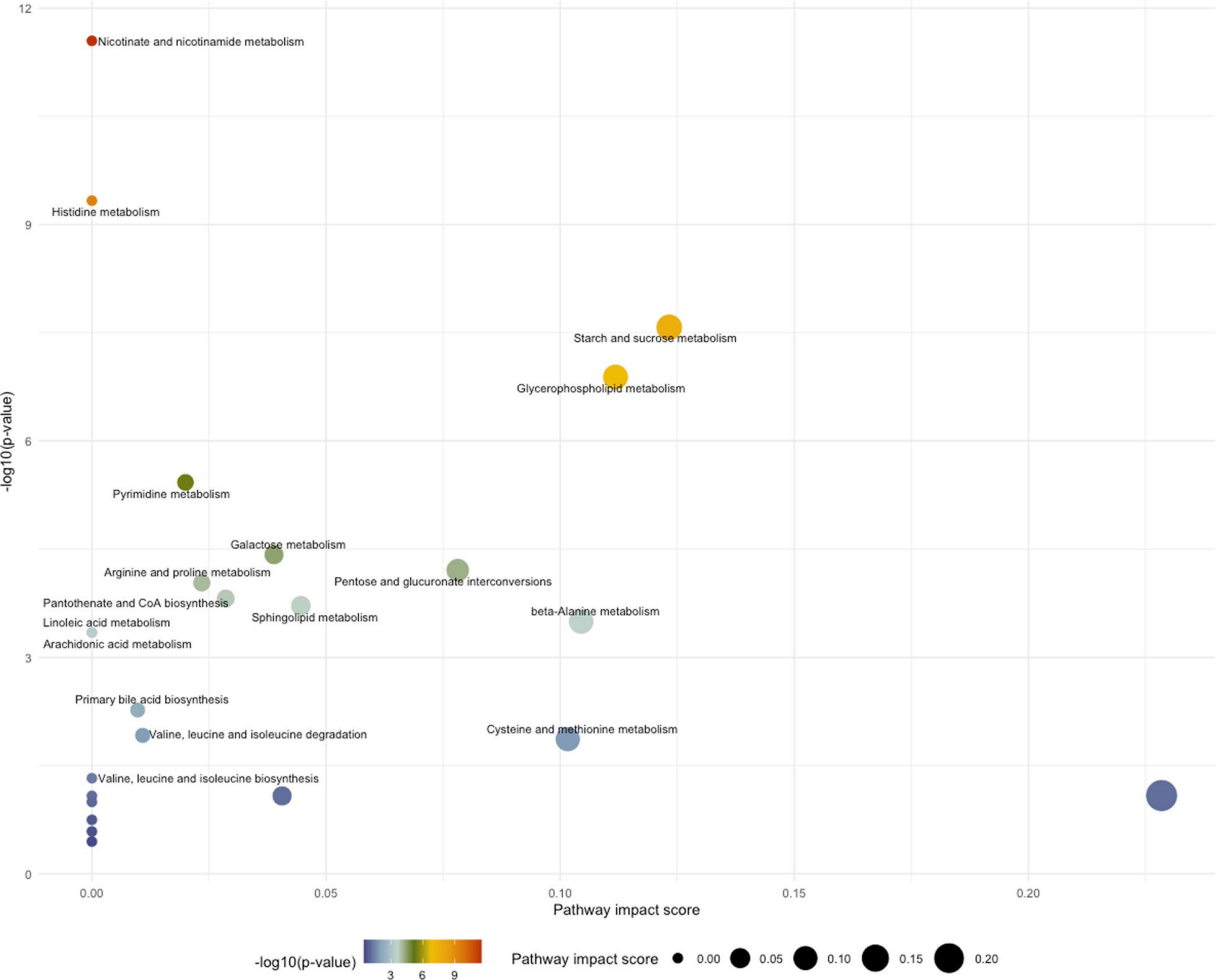
Scatterplots generated from pathway enrichment analysis in MetaboAnalyst. FDR p-values are on the y-axis and pathway impact values on the x-axis. The size of the plotted point correlates to the pathway impact and color (blue to red) correlates to p-values

The top 25 positively-associated and 25 negatively-associated metabolites are shown in Table 2**.** Positively-associated metabolites included the aminosugars *N*-acetylneuraminate and erythronate, the nucleotide pseudouridine, and the amino acid derivatives *C*-glycosyl tryptophan and *N*-acetylserine. Enrichment analysis of these positively-associated metabolites showed overrepresentation of metabolites involved in nicotinate and nicotinamide, histidine, starch and sucrose, and pyrimidine pathways. Negatively-associated metabolites included eight phosphatidylcholines, seven lysophospholipids, and four sphingomyelins; some of these metabolites were represented in the glycerophospholipid and sphingolipid pathways that were significant in the enrichment analysis (Table 3**, **Fig. 4).

**Table 2:** The 25 metabolites that are most positively-associated and 25 metabolites that are most negatively-associated with quantitative interstitial abnormalities in multivariable linear regression models

Metabolite	HMDB ID*	Percent QIA per unit metabolite, *mean [CI]*^*†*^	FDR p-value	Metabolon class
Positively-associated with QIA				
N-acetylneuraminate	0000230	1.41 [0.80 to 2.02]	< 0.001	Carbohydrate; Aminosugar Metabolism
Pseudouridine	0000767	1.35 [0.61 to 2.09]	0.009	Nucleotide; Pyrimidine Metabolism, Uracil containing
C-glycosyltryptophan	0240296	1.31 [0.69 to 1.93]	0.002	Amino Acid; Tryptophan Metabolism
Erythronate	0000613	1.23 [0.59 to 1.87]	0.005	Carbohydrate; Aminosugar Metabolism
N-acetylserine	0002931	1.23 [0.55 to 1.90]	0.001	Amino Acid; Glycine, Serine and Threonine Metabolism
N-acetyl-L-methionine	0011745	1.14 [0.57 to 1.72]	0.004	Amino Acid; Methionine, Cysteine, SAM and Taurine Metabolism
5–6-Dihydrouridine	0000497	1.11 [0.46 to 1.76]	0.015	Nucleotide; Pyrimidine Metabolism, Uracil containing
N-acetylthreonine	0062557	1.01 [0.40 to 1.62]	0.021	Amino Acid; Glycine, Serine and Threonine Metabolism
N2, N2-Dimethylguanosine	0004824	1.01 [0.35 to 1.66]	0.030	Nucleotide; Purine Metabolism, Guanine containing
Cytidine	0000089	0.98 [0.48 to 1.48]	0.004	Nucleotide; Pyrimidine Metabolism, Cytidine containing
Hydroxyasparagine	0341329	0.98 [0.32 to 1.64]	0.038	Amino Acid; Alanine and Aspartate Metabolism
Sphingosine	0000252	0.94 [0.40 to 1.47]	0.013	Lipid; Sphingosines
N-Acetylgalactosamine, N-Acetyl-D-glucosamine^‡^	0000212, 000021^‡^	0.92 [0.37 to 1.47]	0.018	Carbohydrate; Aminosugar Metabolism
N4-Acetylcytidine	0005923	0.89 [0.49 to 1.30]	0.001	Nucleotide; Pyrimidine Metabolism, Cytidine containing
N-Acetylputrescine	0002064	0.89 [0.30 to 1.49]	0.035	Amino Acid; Polyamine Metabolism
N-acetyl-isoputreanine		0.87 [0.44 to 1.29]	0.003	Amino Acid; Polyamine Metabolism
Succinylcarnitine	0061717	0.85 [0.37 to 1.32]	0.012	Energy; TCA Cycle
1-Methyl-4-imidazoleacetate	0002820	0.81 [0.35 to 1.28]	0.013	Amino Acid; Histidine Metabolism
3-ureido-Propionate	0000026	0.78 [0.43 to 1.14]	0.001	Nucleotide; Pyrimidine Metabolism, Uracil containing
Quinolinate	0000232	0.73 [0.37 to 1.08]	0.003	Cofactors and Vitamins; Nicotinate and Nicotinamide Metabolism
Acisoga	0061384	0.66 [0.29 to 1.02]	0.011	Amino Acid; Polyamine Metabolism
5-(galactosylhydroxy)-L-lysine		0.61 [0.23 to 0.98]	0.025	Amino Acid; Lysine Metabolism
N-acetyltaurine	0240253	0.55 [0.16 to 0.94]	0.048	Amino Acid; Methionine, Cysteine, SAM and Taurine Metabolism
Phenylacetylglutamine	0006344	0.54 [0.23 to 0.85]	0.015	Peptide; Acetylated Peptides
1-Carboxyethyltyrosine		0.51 [0.22 to 0.81]	0.015	Amino Acid; Tyrosine Metabolism
Negatively-associated with QIA				
Phosphatidylcholine (18:0/18:2)	0008039	− 2.09 [− 3.26 to − 0.91]	0.013	Lipid; Phosphatidylcholine (PC)
Phosphatidylcholine (16:0/18:2)	0007973	− 1.92 [− 3.25 to − 0.59]	0.044	Lipid; Phosphatidylcholine (PC)
Lysophosphatidylcholine (16:0/0:0)	0010382	− 1.71 [− 2.76 to − 0.66]	0.024	Lipid; Lysophospholipid
Phosphatidylcholine (18:2/20:4)	0008147	− 1.59 [− 2.21 to − 0.97]	< 0.001	Lipid; Phosphatidylcholine (PC)
Phosphatidylcholine (16:0/18:0)	0007970	− 1.45 [− 2.31 to − 0.59]	0.018	Lipid; Phosphatidylcholine (PC)
Lysophosphatidylcholine (18:0/0:0)	0010384	− 1.45 [− 2.19 to − 0.70]	0.005	Lipid; Lysophospholipid
Sphingomyelin (D18:1/20:0)	0012102	− 1.40 [− 2.22 to − 0.57]	0.017	Lipid; Sphingomyelins
Vitamin A	0000305	− 1.16 [− 1.74 to − 0.57]	0.004	Cofactors and Vitamins; Vitamin A Metabolism
Sphingomyelin (D18:2(4E,14Z)/14:0), Sphingomyelin (D18:1/14:1(9Z))^‡^	0240637, 0240612^‡^	− 1.15 [− 1.63 to − 0.66]	< 0.001	Lipid; Sphingomyelins
Thyroxine	0000248	− 1.13 [− 1.83 to − 0.42]	0.025	Amino Acid; Tyrosine Metabolism
L-alpha-Aminobutyric acid	0000452	− 1.13 [− 1.69 to − 0.57]	0.003	Amino Acid; Glutathione Metabolism
Phosphatidylinositol (18:0/20:4)	0009815	− 1.10 [− 1.80 to − 0.39]	0.028	Lipid; Phosphatidylinositol (PI)
Lysophosphatidylcholine (0:0/16:0)	0061702	− 1.10 [− 1.68 to − 0.53]	0.005	Lipid; Lysophospholipid
3-methyl-2-oxobutyrate	0000019	− 1.06 [− 1.75 to − 0.37]	0.030	Amino Acid; Leucine, Isoleucine and Valine Metabolism
Gamma-glutamyl-2-aminobutyrate		− 1.06 [− 1.48 to − 0.64]	< 0.001	Peptide; Gamma-glutamyl Amino Acid
Lysophosphatidylcholine (18:1/0:0)	0002815	− 1.04 [− 1.69 to − 0.39]	0.025	Lipid; Lysophospholipid
Lysophosphatidylcholine (18:2/0:0)	0010386	− 1.04 [− 1.69 to − 0.39]	0.025	Lipid; Lysophospholipid
Sphingomyelin (D17:2/16:0) Sphingomyelin (D18:2/15:0)^‡^	0240677	− 1.02 [− 1.47 to − 0.58]	< 0.001	Lipid; Sphingomyelins
Arachidoylcarnitine	0006460	− 1.00 [− 1.52 to − 0.48]	0.005	Lipid; Fatty Acid Metabolism (Acyl Carnitine, Long Chain Saturated)
Phosphatidylcholine (18:2/18:2)	0008138	− 0.98 [− 1.53 to − 0.44]	0.010	Lipid; Phosphatidylcholine (PC)
Sphingomyelin (D17:1/14:0), Sphingomyelin (D16:1/15:0)^‡^		− 0.95 [− 1.33 to − 0.57]	< 0.001	Lipid; Sphingomyelins
Ceramide (D18:2/22:0)		− 0.93 [− 1.38 to − 0.47]	0.003	Lipid; Ceramides
Citrulline	0000904	− 0.92 [− 1.56 to − 0.29]	0.043	Amino Acid; Urea cycle; Arginine and Proline Metabolism
Ceramide (D20:1/18:0), Ceramide (D16:1/22:0), Ceramide (D18:1/20:0)^‡^	0240684, 0240682, 0004951^‡^	− 0.91 [− 1.40 to − 0.41]	0.009	Lipid; Ceramides
Lysophosphatidylcholine (24:0/0:0)	0010405	− 0.84 [− 1.25 to − 0.43]	0.003	Lipid; Lysophospholipid

*CI* 95% confidence interval, *FDR* Benjamini–Hochberg False Discovery Rate, *HMDB* Human Metabolome Database, *QIA* quantitative interstitial abnormalities

^*^Wishart DS, Guo AC, Oler E, et al., HMDB 5.0: the Human Metabolome Database for 2022. Nucleic Acids Res. 2022. Jan 7;50(D1):D622–31

^†^Multivariable regression models adjusted for age, sex, body mass index, smoking status, pack-years, and inhaled corticosteroid use

^‡^ Cannot be analytically differentiated

**Table 3:** Pathway enrichment analysis of metabolites associated with quantitative interstitial abnormalities

Pathway*	Total in pathway	Hits		FDR p-value	Pathway impact value
Nicotinate and nicotinamide metabolism	15	1	Quinolinate	6.81E−11	0
Histidine metabolism	16	1	Methylimidazoleacetic acid	5.61E−09	0
Starch and sucrose metabolism	18	2	Sucrose; Maltose	2.14E−07	0.123
Glycerophospholipid metabolism	36	2	Phosphatidylcholine (PC); 1-Acyl-sn-glycero-3-phosphocholine (Lysophosphatidylcholine, LPC)	7.83E−0	0.112
Pyrimidine metabolism	39	2	3-Ureidopropionate; Cytidine	1.81E−05	0.020
Galactose metabolism	27	1	Sucrose	1.50E−04	0.039
Pentose and glucuronate interconversions	18	1	D-Xylose	2.11E−04	0.078
Arginine and proline metabolism	38	1	N-Acetylputrescine	2.77E−04	0.023
Pantothenate and CoA biosynthesis	19	2	3-Ureidopropionate; 3-Methyl-2-oxobutanoic acid	4.05E−04	0.029
Sphingolipid metabolism	21	2	Sphingomyelin; Sphingosine	4.60E−04	0.045
beta-Alanine metabolism	21	1	3-Ureidopropionate	6.93E−04	0.104
Arachidonic acid metabolism	36	1	Phosphatidylcholine (PC)	8.32E−04	0
Linoleic acid metabolism	5	1	Phosphatidylcholine (PC)	8.32E−04	0
Primary bile acid biosynthesis	46	1	Taurochenodeoxycholate	0.009	0.010
Valine, leucine and isoleucine degradation	40	1	3-Methyl-2-oxobutanoic acid	0.02	0.011
Cysteine and methionine metabolism	33	2	(S)-2-Aminobutanoate; 2-Oxobutanoate	0.02	0.102
Valine, leucine and isoleucine biosynthesis	8	2	3-Methyl-2-oxobutanoic acid; 2-Oxobutanoate	0.07	0
Arginine biosynthesis	14	1	Citrulline	0.10	0.228
Propanoate metabolism	23	1	2-Oxobutanoate	0.10	0.041
Glycine, serine and threonine metabolism	33	1	2-Oxobutanoate	0.10	0
Tyrosine metabolism	42	1	Thyroxine	0.12	0
alpha-Linolenic acid metabolism	13	2	Phosphatidylcholine (PC); Stearidonic acid	0.19	0
Biosynthesis of unsaturated fatty acids	36	3	Octadecanoic acid; Docosahexaenoic acid; Icosapentaenoic acid	0.27	0
Fatty acid biosynthesis	47	1	Tetradecanoic acid	0.36	0

*Abbreviations:* FDR = Benjamini–Hochberg False Discovery Rate

*Metabolites with a Human Metabolome Database (HMDB) ID number

### Multinomial outcomes of CT phenotypes

Globally amongst the four phenotypes (QIA-predominant, combined-predominant, emphysema-predominant, neither-predominant), 282 metabolites significantly differed by ANOVA. Post-hoc Tukey’s test pairwise comparisons were performed and are shown in Additional file 1: Table S6.

**Fig. 5:**
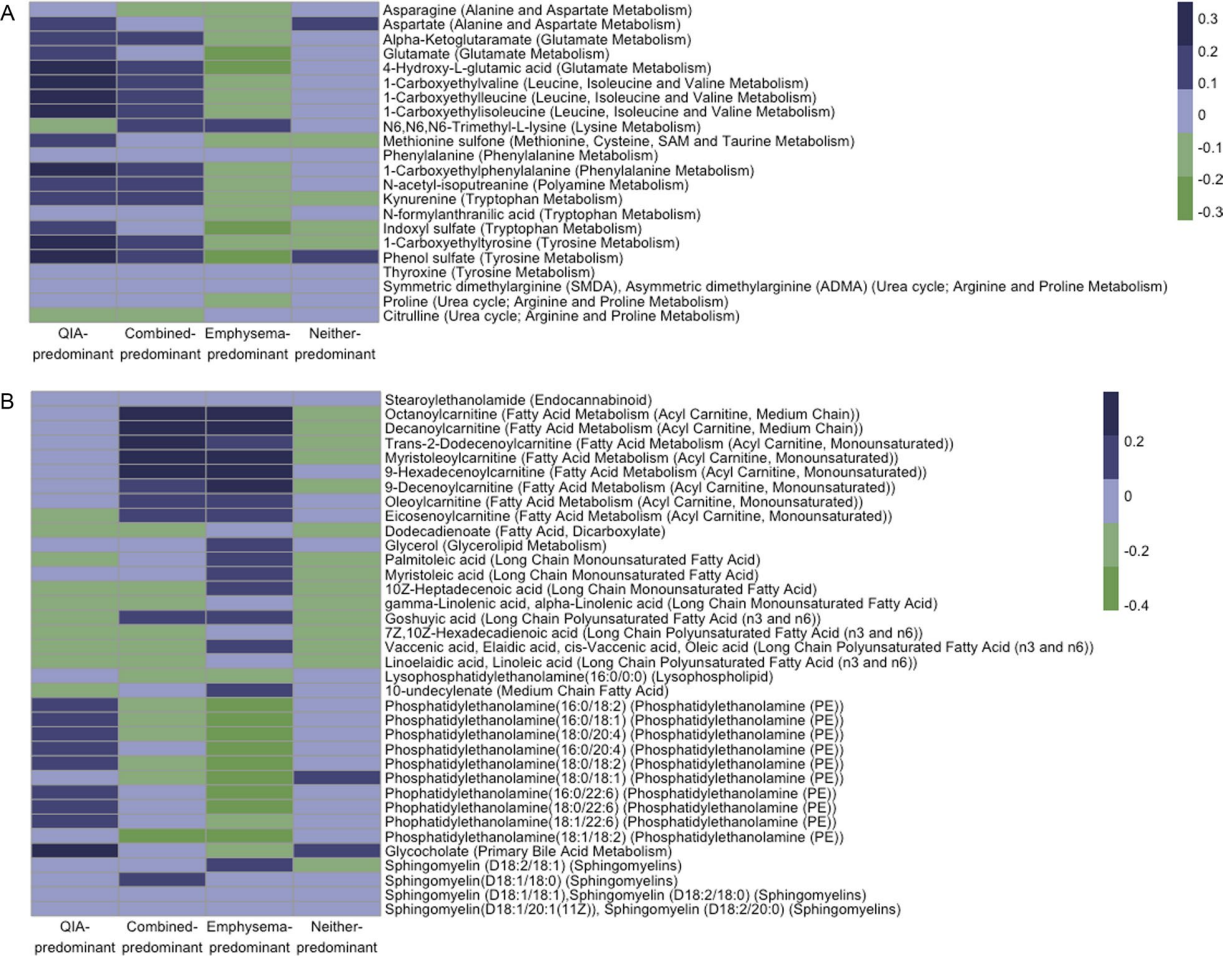
Heatmaps of mean **A** amino acid metabolite values and **B** lipid metabolite values of QIA-predominant, combined-predominant, emphysema-predominant, and neither-predominant groups. Shown are the metabolites that had significant differences in the QIA-predominant and emphysema-predominant groups (FDR p ≤ 0.05)

**Fig. 6:**
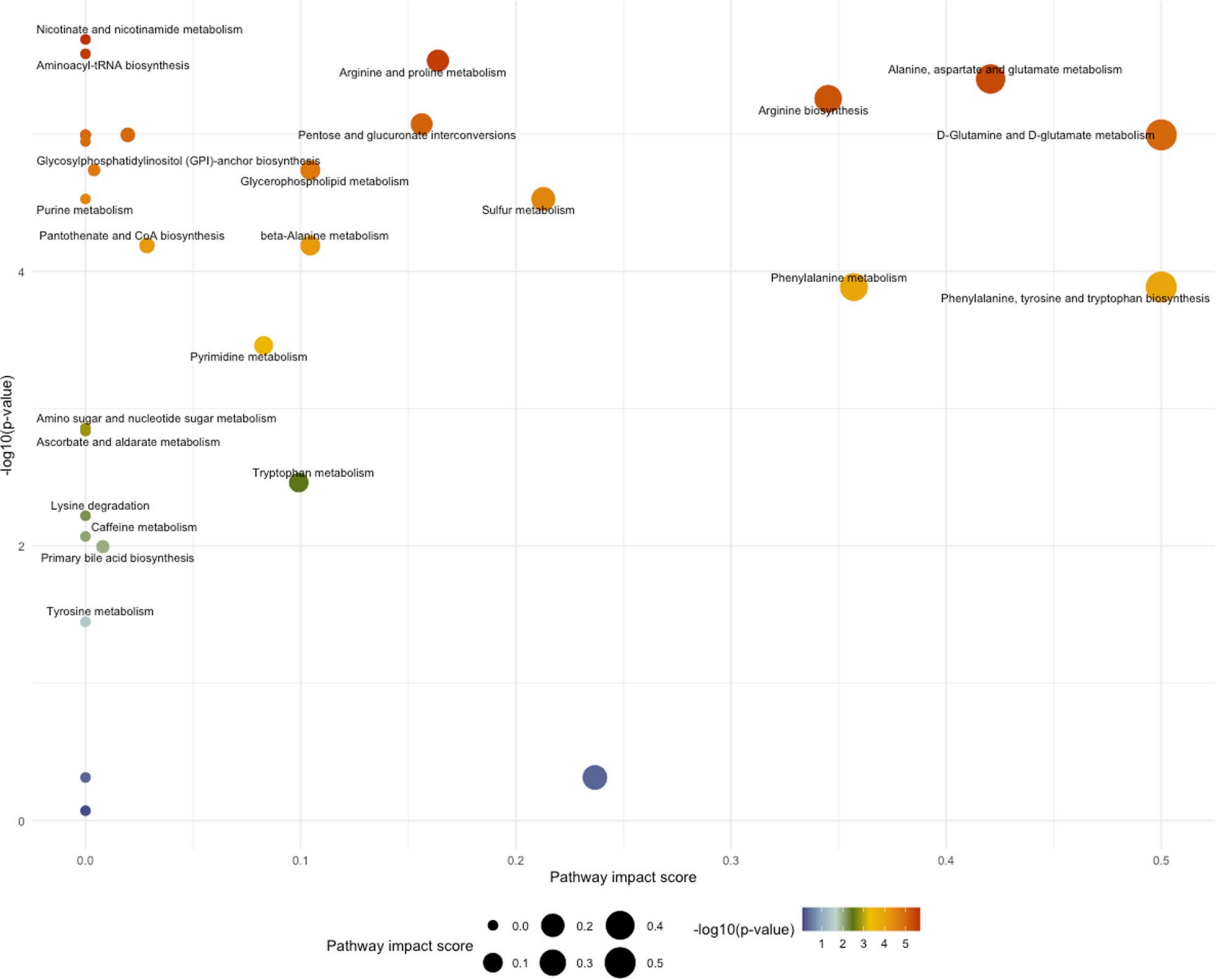
Scatterplots generated from pathway enrichment analysis in MetaboAnalyst of multinomial logistic regression analysis of QIA-predominant and emphysema-predominant phenotypes. FDR p-values are on the y-axis and pathway impact values on the x-axis. The size of the plotted point correlates to pathway impact and colors (blue to red) correlate to p-values

Our adjusted multinomial logistic regression models yielded 75 metabolites that differed significantly between QIA-predominant and emphysema-predominant phenotypes, with 45 associated with higher odds and 30 associated with lower odds of QIA relative to emphysema (Table 4), including 36 (48.0%) lipids and 22 (29.3%) amino acids (Fig. 5**,** Additional file 1: Table S7). Most of the associations of amino acids with QIA-predominance were positive, and they included dimethylarginine (SDMA, ADMA), phenylalanine, asparagine, proline, and kynurenine. Amongst lipids, phosphatidylethanolamines (PE) were most commonly associated with higher odds of QIA-predominance, whereas sphingomyelins (SM) and acyl carnitines were associated with higher odds of emphysema-predominance. Pathway enrichment analysis showed overrepresentation of metabolites involved in PE metabolism (glycerophospholipid and glycosylphosphatidylinositol-anchor pathways), as well as multiple amino acid pathways including those involving nicotinate and nicotinamide, aminoacyl-tRNA, arginine, proline, alanine, aspartate, and glutamate metabolism (Table 5**, **Fig. 6).

**Table 4:** **A** The 25 metabolites that have highest odds of QIA-predominant phenotype and **B** 25 metabolites that have the lowest odds of QIA-predominant phenotype compared to emphysema-predominant phenotype in multinomial logistic regression

Metabolite	HMDB ID*	Odds of QIA-predominant over emphysema-predominant, *mean [CI]*^*†*^	FDR p-value	Metabolon class
Symmetric dimethylarginine (SMDA), Asymmetric dimethylarginine (ADMA)^‡^	0003334, 0001539^‡^	5.19 [2.01–13.40]	0.021	Amino Acid; Urea cycle; Arginine and Proline Metabolism
Phenylalanine	0000159	5.04 [1.73–14.72]	0.040	Amino Acid; Phenylalanine Metabolism
Asparagine	0000168	4.79 [1.99–11.54]	0.018	Amino Acid; Alanine and Aspartate Metabolism
Sulfate	01448	4.05 [1.79–9.17]	0.022	Xenobiotics; Chemical
Proline	0000162, 0003411	3.36 [1.61–7.03]	0.028	Amino Acid; Urea cycle; Arginine and Proline Metabolism
Kynurenine	0000684	2.82 [1.48–5.34]	0.031	Amino Acid; Tryptophan Metabolism
Erythronate	0000613	2.71 [1.46–5.04]	0.031	Carbohydrate; Aminosugar Metabolism
N-acetyl-isoputreanine		2.64 [1.73–4.04]	0.002	Amino Acid; Polyamine Metabolism
Alpha-Ketoglutaramate	0001552	2.57 [1.46–4.50]	0.026	Amino Acid; Glutamate Metabolism
Gamma-Glutamylphenylalanine	0000594	2.49 [1.40–4.42]	0.031	Peptide; Gamma-glutamyl Amino Acid
Phosphatidylethanolamine (16:0/18:2)	0005322	2.37 [1.65–3.40]	< 0.001	Lipid; Phosphatidylethanolamine (PE)
Phosphatidylethanolamine (16:0/18:1)	0005320	2.31 [1.66–3.23]	< 0.001	Lipid; Phosphatidylethanolamine (PE)
Aspartate	0000191	2.25 [1.40–3.61]	0.022	Amino Acid; Alanine and Aspartate Metabolism
Lysophosphatidylethanolamine (16:0/0:0)	0011503	2.16 [1.30–3.58]	0.039	Lipid; Lysophospholipid
Phosphatidylethanolamine (18:0/20:4)	0009003	2.10 [1.39–3.18]	0.018	Lipid; Phosphatidylethanolamine (PE)
Phosphatidylethanolamine (16:0/20:4)	0005323	2.09 [1.43–3.04]	0.011	Lipid; Phosphatidylethanolamine (PE)
3-Ureidopropionate	0000026	1.99 [1.40–2.83]	0.011	Nucleotide; Pyrimidine Metabolism, Uracil containing
N-formylanthranilic acid	0004089	1.97 [1.32–2.95]	0.026	Amino Acid; Tryptophan Metabolism
Phosphatidylethanolamine (18:0/18:2)	0008994	1.94 [1.37–2.74]	0.011	Lipid; Phosphatidylethanolamine (PE)
Succinylcarnitine	0061717	1.92 [1.22–3.01]	0.049	Energy; TCA Cycle
Quinolinate	0000232	1.89 [1.35–2.66]	0.011	Cofactors and Vitamins; Nicotinate and Nicotinamide Metabolism
Gulonate	0003290	1.89 [1.25–2.87]	0.037	Cofactors and Vitamins; Ascorbate and Aldarate Metabolism
Methionine sulfone	0062174	1.85 [1.26–2.70]	0.031	Amino Acid; Methionine, Cysteine, SAM and Taurine Metabolism
1-Carboxyethylphenylalanine		1.81 [1.34–2.43]	0.011	Amino Acid; Phenylalanine Metabolism
Glutamate	0000148	1.80 [1.25–2.61]	0.031	Amino Acid; Glutamate Metabolism
Sphingomyelin (D18:1/20:1(11Z)), Sphingomyelin (D18:2/20:0)^‡^	0240610, 0240632^‡^	0.31 [0.16–0.63]	0.026	Lipid; Sphingomyelins
Thyroxine	0000248	0.32 [0.16–0.64]	0.028	Amino Acid; Tyrosine Metabolism
Sphingomyelin (D18:1/18:1), Sphingomyelin (D18:2/18:0)^‡^	0012101	0.32 [0.16–0.66]	0.031	Lipid; Sphingomyelins
Sphingomyelin (D18:1/18:0)	0001348	0.33 [0.16–0.68]	0.038	Lipid; Sphingomyelins
Citrulline	0000904	0.41 [0.22–0.74]	0.043	Amino Acid; Urea cycle; Arginine and Proline Metabolism
Sphingomyelin (D18:2/18:1)		0.43 [0.26–0.69]	0.020	Lipid; Sphingomyelins
Eicosenoylcarnitine		0.47 [0.31–0.70]	0.011	Lipid; Fatty Acid Metabolism (Acyl Carnitine, Monounsaturated)
Oleoylcarnitine	0005065	0.49 [0.30–0.80]	0.049	Lipid; Fatty Acid Metabolism (Acyl Carnitine, Monounsaturated)
Stearoylethanolamide	0013078	0.50 [0.31–0.80]	0.045	Lipid; Endocannabinoid
Glycerol	0000131	0.53 [0.38–0.74]	0.011	Lipid; Glycerolipid Metabolism
N6,N6,N6-Trimethyl-L-lysine	0001325	0.55 [0.38–0.81]	0.033	Amino Acid; Lysine Metabolism
Homostachydrine	0033433	0.59 [0.43–0.81]	0.024	Xenobiotics; Food Component/Plant
9-Decenoylcarnitine	0013205	0.61 [0.44–0.84]	0.037	Lipid; Fatty Acid Metabolism (Acyl Carnitine, Monounsaturated)
9-Hexadecenoylcarnitine	0013207	0.62 [0.45–0.86]	0.049	Lipid; Fatty Acid Metabolism (Acyl Carnitine, Monounsaturated)
10-undecylenate	0033724	0.63 [0.47–0.85]	0.037	Lipid; Medium Chain Fatty Acid
Myristoleoylcarnitine	0240588	0.63 [0.49–0.82]	0.018	Lipid; Fatty Acid Metabolism (Acyl Carnitine, Monounsaturated)
Trans-2-Dodecenoylcarnitine	13,326	0.64 [0.49–0.84]	0.031	Lipid; Fatty Acid Metabolism (Acyl Carnitine, Monounsaturated)
Dodecadienoate		0.66 [0.52–0.85]	0.028	Lipid; Fatty Acid, Dicarboxylate
Decanoylcarnitine	0000651	0.67 [0.52–0.87]	0.034	Lipid; Fatty Acid Metabolism (Acyl Carnitine, Medium Chain)
Octanoylcarnitine	0000791	0.68 [0.53–0.88]	0.037	Lipid; Fatty Acid Metabolism (Acyl Carnitine, Medium Chain)
Linoelaidic acid, Linoleic acid^‡^	0006270, 0000673^‡^	0.69 [0.54–0.87]	0.033	Lipid; Long Chain Polyunsaturated Fatty Acid (n3 and n6)
gamma-Linolenic acid, alpha-Linolenic acid^‡^	0003073, 0001388^‡^	0.71 [0.57–0.88]	0.031	Lipid; Long Chain Polyunsaturated Fatty Acid (n3 and n6)
Vaccenic acid, Elaidic acid, cis-Vaccenic acid, Oleic acid^‡^	0003231, 0000573, 0240219, 0000207^‡^	0.71 [0.56–0.90]	0.049	Lipid; Long Chain Monounsaturated Fatty Acid
10Z-Heptadecenoic acid	0060038	0.74 [0.61–0.90]	0.041	Lipid; Long Chain Monounsaturated Fatty Acid
7Z,10Z-Hexadecadienoic acid	0000477	0.74 [0.61–0.89]	0.031	Lipid; Long Chain Polyunsaturated Fatty Acid (n3 and n6)

*CI* 95% confidence interval, *FDR* Benjamini–Hochberg False Discovery Rate, *HMDB* Human Metabolome Database, *QIA* quantitative interstitial abnormalities

^*^Wishart DS, Guo AC, Oler E, et al., HMDB 5.0: the Human Metabolome Database for 2022. Nucleic Acids Res. 2022. Jan 7;50(D1):D622–31

^†^Multinomial regression models adjusted for age, sex, body mass index, smoking status, pack-years, and inhaled corticosteroid use. QIA-predominant was initially used as the reference group

^‡^Cannot be analytically differentiated

**Table 5:** Pathway enrichment analysis of metabolites associated QIA-predominant versus emphysema-predominant phenotypes from multinomial regression

Pathway*	Total in pathway	Hits		FDR p-value	Pathway impact value
Nicotinate and nicotinamide metabolism	15	2	Aspartate; Quinolinate	2.71E−05	0
Aminoacyl-tRNA biosynthesis	48	5	Asparagine; Phenylalanine; Aspartate; Proline; Glutamate	2.71E−05	0
Arginine and proline metabolism	38	2	Proline; Glutamate	2.71E−05	0.164
Alanine, aspartate and glutamate metabolism	28	4	Aspartate; Asparagine; Glutamate; 2-Oxoglutaramate	2.71E−05	0.421
Arginine biosynthesis	14	3	Glutamate; Citrulline; Aspartate	2.71E−05	0.345
Pentose and glucuronate interconversions	18	2	Gulonate; D-Xylose	2.71E−05	0.156
D-Glutamine and D-glutamate metabolism	6	1	Glutamate	2.71E−05	0.500
Glutathione metabolism	28	1	Glutamate	2.71E−05	0.020
Glyoxylate and dicarboxylate metabolism	32	1	Glutamate	2.71E−05	0
Butanoate metabolism	15	1	Glutamate	2.71E−05	0
Porphyrin and chlorophyll metabolism	30	1	Glutamate	2.71E−05	0
Nitrogen metabolism	6	1	Glutamate	2.71E−05	0
Histidine metabolism	16	2	Glutamate; Aspartate	2.79E−05	0
Glycerophospholipid metabolism	36	1	Phosphatidylethanolamine	3.91E−05	0.104
Glycosylphosphatidylinositol (GPI)-anchor biosynthesis	14	1	Phosphatidylethanolamine	3.91E−05	0.004
Sulfur metabolism	8	1	Sulfate	5.60E−05	0.213
Purine metabolism	65	1	Sulfate	5.60E−05	0
beta-Alanine metabolism	21	2	Aspartate; 3-Ureidopropionate	1.09E−04	0.104
Pantothenate and CoA biosynthesis	19	2	Aspartate; 3-Ureidopropionate	1.09E−04	0.029
Phenylalanine, tyrosine and tryptophan biosynthesis	4	1	Phenylalanine	1.99E−04	0.500
Phenylalanine metabolism	10	1	Phenylalanine	1.99E−04	0.357
Pyrimidine metabolism	39	2	Orotidine 5'-phosphate; 3-Ureidopropionate	5.06E−04	0.083
Amino sugar and nucleotide sugar metabolism	37	1	beta-D-Fructose	0.002	0
Ascorbate and aldarate metabolism	8	1	Gulonate	0.002	0
Tryptophan metabolism	41	2	Kynurenine; Formylanthranilate	0.004	0.099
Lysine degradation	25	1	N6,N6,N6-Trimethyl-L-lysine	0.007	0
Caffeine metabolism	10	1	7-Methylxanthine	0.01	0
Primary bile acid biosynthesis	46	1	Glycocholate	0.01	0.008
Tyrosine metabolism	42	1	Thyroxine	0.04	0
Glycerolipid metabolism	16	1	Glycerol	0.50	0.237
Galactose metabolism	27	1	Glycerol	0.50	0
Sphingolipid metabolism	21	1	Sphingomyelin	0.85	0

*FDR* Benjamini–Hochberg False Discovery Rate

*Metabolites with a Human Metabolome Database (HMDB) ID number

One metabolite, tryptophan betaine, was significantly associated with lower odds of QIA-predominant compared to neither-predominant groups. There were no significant metabolites between the QIA-predominant and combined-predominant groups. Intriguingly, despite non-significance, and although the unadjusted mean metabolite levels do not completely reflect the multinomial differences, the combined-predominant group had similar levels of amino acids as the QIA-predominant group but, depending on the metabolite, showed directionality with either the QIA-predominant or the emphysema-predominant group (Fig. 5).

## Discussion

To our knowledge, our discovery study was the first global analysis of the metabolomics features of quantitative interstitial abnormalities in a large cohort. Our study of 928 ever-smokers in COPDGene found that 85 plasma metabolites from the Metabolon metabolomics assay were associated with QIA, independent of age, sex, BMI, smoking status, pack-years, and ICS use. These findings highlight the metabolic features of participants with QIA and provide initial insight into the biochemical systemic features associated with these quantitative CT changes. Furthermore, we identified 75 metabolites that differed significantly between participants with QIA-predominant versus emphysema-predominant phenotypes. Such associations of metabolomic differences between participants with shared risk factors but different CT parenchymal findings may be useful as biomarkers that distinguish these smoking-related phenotypes. These associations also help us understand the metabolic processes that may be important in early QIA, but which may be less prominent in later stages of lung injury like emphysema. Lastly, some of the metabolites significant in our analyses were those previously associated with advanced diseases like IPF and COPD, suggesting potential shared pathways in progression that should be studied further.

### Amino acids

Circulating amino acids are involved in numerous processes including cell signaling, regulating gene expression, hormone synthesis and secretion, nutrient metabolism, oxidative stress, and immune response regulation [32]. In particular, smokers with COPD can have perturbations in branched chain amino acid levels important in the skeletal muscle, which may reflect systemic changes including impaired immunity or cachexia. [17]

In our analysis, the tryptophan metabolites quinolinate, kynurenine, and *N*-formylanthranilic acid were associated with higher odds of QIA over emphysema and were notable. These tryptophan derivatives suggest inflammatory activity with QIA and shared overlap with advanced diseases. These three metabolites are downstream in the kynurenine pathway, which normally comprises 95% of tryptophan metabolism and is upregulated in inflammation and immune responses [33]. Quinolinate is also a substrate for nicotinamide adenine dinucleotide (NAD +) synthesis, required for normal cell function and energy production, and this pathway has been proposed to be upregulated in physiological stress [34] and was enriched for QIA in our analysis. In COPD, upregulated kynurenine derivatives are associated with reduced FEV1 [35], and reduction in the precursor tryptophan is associated with COPD exacerbations and emphysema [17]. Patients with IPF have been shown to have significant declines in kynurenine after treatment with the anti-fibrotic pirfenidone, thought to be due to its anti-inflammatory effects. [36]

Several glutamine derivatives were associated with higher odds of QIA over emphysema and in pathway enrichment: glutamate, alpha-ketoglutarate, and 4-hydroxyglutamate. The precursor glutamine is the most abundant amino acid in the body, found in both plasma and skeletal muscle, and plays roles in immune modulation, ammonia transport, and maintenance of cell integrity and function [37]. The downstream derivatives glutamate and alpha-ketoglutarate are crucial intermediates in the Krebs cycle [37]. In patients with COPD compared to controls, plasma glutamine and glutamate are decreased, thought to be from hypermetabolism and muscle depletion [38]. We found higher odds of higher levels of glutamine derivatives in QIA-predominance compared to emphysema-predominance, which suggests that patients with emphysema may be in a more advanced, catabolic state compared those with QIA. These metabolites should be studied as a potential factor in the progression of QIA to advanced disease.

### Lipids

Circulating lipids comprise thousands of individual species with a considerable range of structural diversity and physiological functions, including maintaining the integrity of the lipid bilayer, functional hormones, and cell signaling pathways [39]. In the lungs, lipids are important components of surfactant. [40]

The majority of lipids negatively-associated with QIA were phospholipids, including eight phosphatidylcholines (PCs), significant in enrichment analysis. PCs are the body’s most abundant phospholipids and the major component of surfactant [41]. Some of the PC species negatively-associated with QIA in our analysis were those specifically found in prior studies of IPF and COPD patients demonstrating decreased PC concentrations in the respiratory fluid and blood [18, 42, 43]. Decreased PC levels may also generally reflect oxidative changes in the lungs in the setting of cigarette exposure, as has been demonstrated in mice alveolar cells [44]. Further studies are needed to test the relationship between blood and pulmonary phospholipids in smokers and to understand whether the plasma PC perturbations associated with QIA represent a systemic manifestation of PC dysregulation in surfactant, or another phospholipid perturbation altogether.

Sphingomyelins (SM) were another lipid subclass negatively-associated with QIA, and they were of particular interest given their many roles in fetal lung development and lung inflammation [45]. Patients with IPF have downregulated plasma SM [46], including the SM(D18:1/20:0) species that we identified with QIA. A previous study of COPD phenotypes in the COPDGene cohort found that some SMs were also negatively-associated with emphysema [47]. In our analysis, four SMs were associated with lower odds of QIA-predominance compared to emphysema-predominance. Our findings complement the prior study as it did not account for QIA in the assessment of emphysema, suggesting that both QIA and emphysema CT measures should be considered when studying the metabolomics of smoking-related disease.

### Carbohydrates

Amongst carbohydrates, our pathway analyses showed QIA was enriched for certain sugars including maltose, sucrose, and xylose. As these sugars mostly come from the diet and the breakdown of food starches in the digestive system, these metabolites may be related to the gut-lung axis, in which changes in inflammation and microbiota in the gut mucosa cross-talk with lung mucosa [48]. Also positively-associated with QIA was the sialic acid amino sugar *N*-acetylneuraminate (Neu5Ac), which may be reflective of inflammation. Sialic acids are often the terminal sugars on mucin, and variations in these sugar attachments may indicate regulation by proinflammatory cytokines or modification by bacteria. [49] Higher Neu5Ac levels in bronchoalveolar lavage fluid have been found to be associated with COPD and with increased bacterial binding in smokers [50, 51]. Lastly, erythronate and its precursor *N*-acetylglucosamine were also positively-associated with QIA. These extracellular matrix degradation products are associated with pulmonary fibrosis in animal models [52], and they may reflect remodeling during QIA development.

### Strengths and limitations

With more patients with a smoking history receiving screening CT scans than before, we need a deeper understanding of the biology that underlies the subtle interstitial changes that are often caught incidentally. The metabolites significant in our exploratory study provide initial insight into the biochemical activity and pathways associated with QIA. We found associations with several metabolomic features previously linked with IPF and COPD, suggestive of shared disease activity between early-stage QIA and later-stage advanced diseases.

We also identified metabolic features that differed between participants with QIA- and emphysema-predominant phenotypes, which provide initial insight into possible common and different underlying pathways. The two smoking-related phenotypes share risk factors and imaging and physiologic features, especially before very advanced disease develops [53]. Since the metabolomic levels of the combined-predominant group did not clearly fall in between those of the QIA- and emphysema-predominant phenotypes, their metabolomic profiles reflect more complicated processes requiring further investigation.

There are several limitations to our study. Although one of the strengths of our study is the large sample size of thoroughly phenotyped ever-smokers, our results need replication in other smoking and population-based cohorts for validation of potential biomarkers. Our analyses can provide insight into, and generate hypotheses for, possible pathogenic pathways of QIA but cannot be used to elucidate exact mechanisms. Furthermore, while the global metabolomics panel captures a broad range of different classes of metabolites, it is not quantitative; in future work aimed at pinpointing mechanisms, targeted assays will be required. Due to the cross-sectional nature of our data, interpretations of causality are limited; longitudinal studies may help elucidate temporal relationships more clearly. We defined CT phenotypes with the predominant CT features occupying at least 5% of the lung volume; although a binary cutoff for emphysema at 5% is well-established as a clinically meaningful value [26], a similar 5% cutoff has been used for QIA but is less robust [1]. Lastly, we used the HMDB identifier and KEGG background database for our pathway analysis because they are widely used, acknowledging the following limitations. Given the novelty of high-throughput metabolomics and rapid accumulation of new data in the field, some metabolites are unclearly notated or not found at all in the databases, other metabolites are redundant in multiple pathways [54]; thus, we may have not been able to detect some pathways that are nonetheless biologically important in QIA.

## Conclusions

Lipid, amino acid, and carbohydrate metabolites associated with inflammation and immune response, extracellular matrix remodeling, surfactant, and muscle cachexia may play important roles in the earliest stages of smoking-related lung disease. These metabolic signals provide initial insight into the biochemical associations with QIA as one of the earliest stages of smoking-related lung disease activity. These signals suggest future biomarkers for early detection of disease and potential therapeutic targets before progression to IPF and COPD.

### Supplementary Information

**Additional file 1. Figure S1.** Participants categorized into four CT-based phenotypes, defining those with ≥5% QIA and <5% emphysema as QIA-predominant, ≥5% QIA and ≥5% emphysema as combined-predominant, <5% QIA and ≥5% emphysema as emphysema-predominant, and <5% QIA and <5% emphysema as neither-predominant. **Table S1. **Distribution of all available metabolites in our analysis, by Metabolon class and sub-class. **Table S2.** Baseline characteristics, by CT phenotype. **Table S3. **Baseline characteristic of COPDGene cohort of smokers with CT measurements and exam data, with (our study cohort) and without metabolomics data collected. **Table S4: **Univariate regression of each metabolite with continuous percent QIA. **Table S5: **Multivariable regression of each metabolite with continuous percent QIA. **Table S6:** Analysis of Variance analysis of each metabolite with CT phenotypes, arranged by Metabolon classes and sub-classes. **Table S7. **Multinomial logistic regression of each metabolite with the CT phenotypes predominant QIA versus predominant emphysema.

## Data Availability

Data is available from the authors upon request and with permission from the COPDGene study group.

## Abbreviations

ANOVA: Analysis of variance
BMI: Body mass index
COPD: Chronic obstructive pulmonary disease
CT: Computed tomography
FEV1: Forced expiratory volume in 1s
FVC: Forced vital capacity
HMDB: Human Metabolome Database
ICS: Inhaled corticosteroid
IPF: Idiopathic pulmonary fibrosis
KEGG: Kyoto encyclopedia of genes and genomes
PC: Phosphatidylcholines
PE: Phosphatidylethanolamines
QIA: Quantitative interstitial abnormalities
SM: Sphingomyelins
