# Supplementary material for: Plasma metabolomics and quantitative interstitial abnormalities in ever-smokers
Source: Respir Res. 2023 Nov 4;24:265. doi: 10.1186/s12931-023-02576-2 (PMC10625195; doi:10.1186/s12931-023-02576-2)
Supplement: Supplementary file 1 — Additional file 1. Figure S1. Participants categorized into four CT-based phenotypes, defining those with ≥5% QIA and <5% emphysema as QIA-predominant, ≥5% QIA and ≥5% emphysema as combined-predominant, <5% QIA and ≥5% emphysema as emphysema-predominant, and <5% QIA and <5% emphysema as neither-predominant. Table S1. Distribution of all available metabolites in our analysis, by Metabolon class and sub-class. Table S2. Baseline characteristics, by CT phenotype. Table S3. Baseline characteristic of COPDGene cohort of smokers with CT measurements and exam data, with (our study cohort) and without metabolomics data collected. Table S4: Univariate regression of each metabolite with continuous percent QIA. Table S5: Multivariable regression of each metabolite with continuous percent QIA. Table S6: Analysis of Variance analysis of each metabolite with CT phenotypes, arranged by Metabolon classes and sub-classes. Table S7. Multinomial logistic regression of each metabolite with the CT phenotypes predominant QIA versus predominant emphysema. [file 12931_2023_2576_MOESM1_ESM.docx]

**Additional file 1: METHODS**

The Genetic Epidemiology of COPD (COPDGene) is a prospective cohort of over 10,300 white and black ever-smokers with at least a 10-pack-year smoking history (and 457 never-smoker controls), aged 45-80 years, without prior bronchiectasis or ILD, from 21 study centers in the United States.^1^ For this current study, only current or former smokers were included. At five-year follow-up visit (visit 2, July 2013 – July 2017), 6,284 participants that returned underwent collection of inspiratory and expiratory chest CT scans, pre- and post-bronchodilator spirometric testing, questionnaires, and blood collection.^2,3^ CT scans were obtained at inspiration (200 milliampere-seconds, mA) and after expiration (50 mA) with submillimeter slice reconstruction.^1^

At the visit, from two study centers (National Jewish Health, University of Iowa), 1,136 participants underwent collection of fresh frozen plasma collected using an 8.5 mL p100 tube (Becton Dickinson).^4^ Metabolites were profiled from the plasma under a standard procedure using Metabolon Global Metabolomics Platform (Morrisville, NC), as previously described.^4-7^ Briefly, samples were extracted with methanol (Glen Mills GenoGrinder 2000) and centrifugation. The samples were then divided into five subsets for analysis: two for analysis by two separate reverse-phase/ultrahigh- performance liquid chromatography/tandem mass spectrometry (RP/UPLC-MS/MS) methods with positive ion mode electrospray ionization (ESI), one for analysis by RP/UPLC-MS/MS with negative ion mode ESI, one for analysis by hydrophilic interaction liquid chromatography (HILIC)/UPLC-MS/MS with negative ion mode ESI. One subset was reserved for backup. Experimental peaks were by Metabolon software and matched against an in-house library that includes authentic standards and routinely-detected unknown compounds. The platform reported 1,392 metabolites that were grouped into classes (called “super pathways”) and sub-classes (called “sub pathways”).

There are advantages of using plasma for our analysis. Metabolon prefers EDTA preserved plasma over other blood-related biofluids. There are a number of reasons for this including that serum is more prone to hemolysis which can be highly variable across specimens.^8,9^ Prior studies have also shown good reproducibility of plasma metabolites measured by a liquid chromatography/tandem mass spectrometry metabolomics platform, even after delayed processing.^10^

The COPDGene study (NCT00608764) was approved by the institutional review board (IRB) for ethical review at all 21 participating centers. All participants provided written informed consent. The IRB and protocol numbers are as follows: Ann Arbor VA Medical Center 2014-060462 (Ann Arbor VA IRB); Baylor College of Medicine H-22209 (IRB for Baylor College of Medicine); Brigham and Women's Hospital 2007P000554 (Partners Human Research Committee); Columbia University Medical Center AAAC9324 (Columbia University IRB); Duke University Medical Center Pro00004464 (Duke University Health System IRB); Johns Hopkins University NA_00011524 (Johns Hopkins Medicine IRB); L.A. Biomedical Research Inst. 12756-03 (John F. Wolf, M.D. Human Subjects Committee); Michael E. DeBakey VAMC H-22202 (Institutional Review Board for Human Subject Research for Baylor College of Medicine and Affiliated Hospitals); Minneapolis VA Medical Center 4128-A (Minneapolis VA Health Care System Minnesota); Health Partners Twin Cities 07-127 (Health Partners IRB); Morehouse School of Medicine 97826 (Morehouse School of Medicine IRB); National Jewish Health 1883a (National Jewish Health IRB); Reliant Medical Group (Fallon) 1441 (Reliant Medical Group IRB); Temple University 21659 (Temple IRB); University of Alabama, Birmingham F070712014 (University of Alabama at Birmingham IRB for Human Use); University of California, San Diego 140070 (UCSD Human Research Protections Program); University of Iowa 200710717 (University of Iowa IRB); University of Michigan HUM00014973 (University of Michigan Medical School IRB); University of Minnesota 0801M24949 (University of Minnesota IRB Human Subjects Committee); University of Pittsburgh #07120059 (University of Pittsburgh IRB); and UTHSC at San Antonio HSC20070644H (UT Health Science Center San Antonio IRB)

**Additional file 1: FIGURE**

**Figure S1:** Participants categorized into four CT-based phenotypes, defining those with ≥5% QIA and <5% emphysema as QIA-predominant, ≥5% QIA and ≥5% emphysema as combined-predominant, <5% QIA and ≥5% emphysema as emphysema-predominant, and <5% QIA and <5% emphysema as neither-predominant.


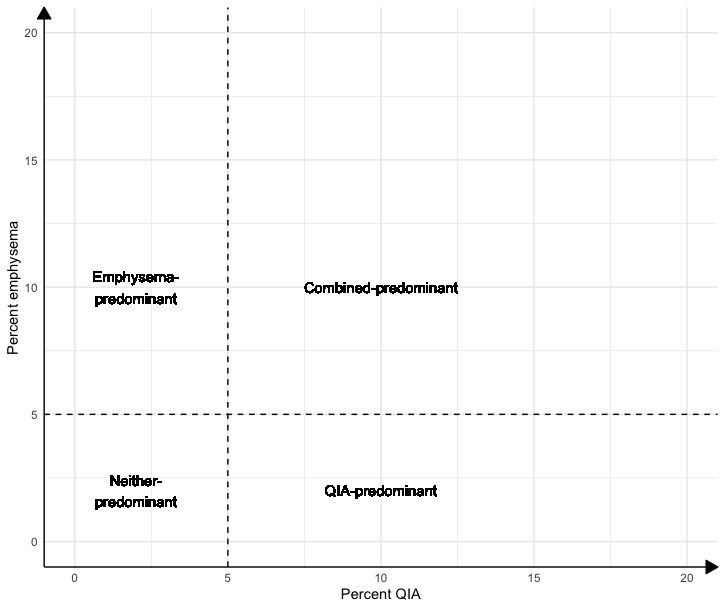


**Additional file 1: TABLES**

**Table S1:** Distribution of all available metabolites in our analysis, by Metabolon class and sub-class

| **Class** | **Sub-class** | **N in visit 2** |
| --- | --- | --- |
| Amino Acid | Alanine and Aspartate Metabolism | 8 |
| Amino Acid | Creatine Metabolism | 3 |
| Amino Acid | Glutamate Metabolism | 11 |
| Amino Acid | Glutathione Metabolism | 7 |
| Amino Acid | Glycine, Serine and Threonine Metabolism | 10 |
| Amino Acid | Guanidino and Acetamido Metabolism | 1 |
| Amino Acid | Histidine Metabolism | 13 |
| Amino Acid | Leucine, Isoleucine and Valine Metabolism | 31 |
| Amino Acid | Lysine Metabolism | 12 |
| Amino Acid | Methionine, Cysteine, SAM and Taurine Metabolism | 17 |
| Amino Acid | Phenylalanine Metabolism | 7 |
| Amino Acid | Polyamine Metabolism | 6 |
| Amino Acid | Tryptophan Metabolism | 16 |
| Amino Acid | Tyrosine Metabolism | 17 |
| Amino Acid | Urea cycle; Arginine and Proline Metabolism | 21 |
| Carbohydrate | Advanced Glycation End-product | 1 |
| Carbohydrate | Aminosugar Metabolism | 5 |
| Carbohydrate | Disaccharides and Oligosaccharides | 1 |
| Carbohydrate | Fructose, Mannose and Galactose Metabolism | 4 |
| Carbohydrate | Glycogen Metabolism | 1 |
| Carbohydrate | Glycolysis, Gluconeogenesis, and Pyruvate Metabolism | 6 |
| Carbohydrate | Pentose Metabolism | 6 |
| Cofactors and Vitamins | Ascorbate and Aldarate Metabolism | 3 |
| Cofactors and Vitamins | Hemoglobin and Porphyrin Metabolism | 4 |
| Cofactors and Vitamins | Nicotinate and Nicotinamide Metabolism | 5 |
| Cofactors and Vitamins | Pantothenate and CoA Metabolism | 1 |
| Cofactors and Vitamins | Tocopherol Metabolism | 5 |
| Cofactors and Vitamins | Vitamin A Metabolism | 5 |
| Cofactors and Vitamins | Vitamin B6 Metabolism | 2 |
| Energy | Oxidative Phosphorylation | 1 |
| Energy | TCA Cycle | 9 |
| Lipid | Androgenic Steroids | 14 |
| Lipid | Carnitine Metabolism | 2 |
| Lipid | Ceramides | 11 |
| Lipid | Corticosteroids | 2 |
| Lipid | Diacylglycerol | 19 |
| Lipid | Dihydroceramides | 2 |
| Lipid | Dihydrosphingomyelins | 5 |
| Lipid | Endocannabinoid | 5 |
| Lipid | Fatty Acid Metabolism (Acyl Carnitine, Dicarboxylate) | 5 |
| Lipid | Fatty Acid Metabolism (Acyl Carnitine, Hydroxy) | 3 |
| Lipid | Fatty Acid Metabolism (Acyl Carnitine, Long Chain Saturated) | 8 |
| Lipid | Fatty Acid Metabolism (Acyl Carnitine, Medium Chain) | 5 |
| Lipid | Fatty Acid Metabolism (Acyl Carnitine, Monounsaturated) | 8 |
| Lipid | Fatty Acid Metabolism (Acyl Carnitine, Polyunsaturated) | 5 |
| Lipid | Fatty Acid Metabolism (Acyl Carnitine, Short Chain) | 1 |
| Lipid | Fatty Acid Metabolism (Acyl Choline) | 4 |
| Lipid | Fatty Acid Metabolism (Acyl Glutamine) | 1 |
| Lipid | Fatty Acid Metabolism (Acyl Glycine) | 2 |
| Lipid | Fatty Acid Metabolism (also BCAA Metabolism) | 5 |
| Lipid | Fatty Acid Synthesis | 1 |
| Lipid | Fatty Acid, Amide | 2 |
| Lipid | Fatty Acid, Amino | 3 |
| Lipid | Fatty Acid, Branched | 2 |
| Lipid | Fatty Acid, Dicarboxylate | 23 |
| Lipid | Fatty Acid, Dihydroxy | 2 |
| Lipid | Fatty Acid, Monohydroxy | 20 |
| Lipid | Glycerolipid Metabolism | 2 |
| Lipid | Hexosylceramides (HCER) | 7 |
| Lipid | Inositol Metabolism | 1 |
| Lipid | Ketone Bodies | 1 |
| Lipid | Lactosylceramides (LCER) | 3 |
| Lipid | Long Chain Monounsaturated Fatty Acid | 7 |
| Lipid | Long Chain Polyunsaturated Fatty Acid (n3 and n6) | 14 |
| Lipid | Long Chain Saturated Fatty Acid | 7 |
| Lipid | Lysophospholipid | 24 |
| Lipid | Lysoplasmalogen | 3 |
| Lipid | Medium Chain Fatty Acid | 8 |
| Lipid | Mevalonate Metabolism | 1 |
| Lipid | Monoacylglycerol | 3 |
| Lipid | Phosphatidylcholine (PC) | 19 |
| Lipid | Phosphatidylethanolamine (PE) | 11 |
| Lipid | Phosphatidylglycerol (PG) | 1 |
| Lipid | Phosphatidylinositol (PI) | 6 |
| Lipid | Phospholipid Metabolism | 6 |
| Lipid | Plasmalogen | 11 |
| Lipid | Pregnenolone Steroids | 4 |
| Lipid | Primary Bile Acid Metabolism | 8 |
| Lipid | Progestin Steroids | 4 |
| Lipid | Secondary Bile Acid Metabolism | 15 |
| Lipid | Short Chain Fatty Acid | 1 |
| Lipid | Sphingolipid Synthesis | 3 |
| Lipid | Sphingomyelins | 28 |
| Lipid | Sphingosines | 2 |
| Lipid | Sterol | 3 |
| Nucleotide | Purine Metabolism, (Hypo)Xanthine/Inosine containing | 6 |
| Nucleotide | Purine Metabolism, Adenine containing | 4 |
| Nucleotide | Purine Metabolism, Guanine containing | 2 |
| Nucleotide | Pyrimidine Metabolism, Cytidine containing | 5 |
| Nucleotide | Pyrimidine Metabolism, Orotate containing | 3 |
| Nucleotide | Pyrimidine Metabolism, Thymine containing | 2 |
| Nucleotide | Pyrimidine Metabolism, Uracil containing | 10 |
| Partially Characterized Molecules | Partially Characterized Molecules | 3 |
| Peptide | Acetylated Peptides | 3 |
| Peptide | Dipeptide | 4 |
| Peptide | Fibrinogen Cleavage Peptide | 1 |
| Peptide | Gamma-glutamyl Amino Acid | 17 |
| Xenobiotics | Bacterial/Fungal | 2 |
| Xenobiotics | Benzoate Metabolism | 21 |
| Xenobiotics | Chemical | 18 |
| Xenobiotics | Drug - Cardiovascular | 1 |
| Xenobiotics | Drug - Topical Agents | 2 |
| Xenobiotics | Food Component/Plant | 38 |
| Xenobiotics | Xanthine Metabolism | 14 |

**Table S2:** Baseline characteristics, by CT phenotype

|  | QIA predominant | Both predominant | Emphysema predominant | Neither predominant |
| --- | --- | --- | --- | --- |
|  | N = 223 | N = 109 | N = 133 | N = 463 |
| Age, mean ± SD | 69.7 ± 8.2 | 72.2 ± 7.0 | 69.8 ± 7.8 | 64.7 ± 8.4 |
| Male | 82 (36.8) | 65 (59.6) | 79 (59.4) | 240 (51.8) |
| Race |  |  |  |  |
| White | 197 (88.3) | 102 (93.6) | 127 (95.5) | 424 (91.6) |
| Black | 26 (11.7) | 7 (6.4) | 6 (4.5) | 39 (8.4) |
| Former Smoker | 154 (69.1) | 89 (81.7) | 120 (90.2) | 325 (70.2) |
| Pack Years, mean ± SD | 44.6 ± 23.5 | 62.3 ± 29.0 | 52.0 ± 26.0 | 38.1 ± 19.9 |
| Body Mass Index (kg/m2), mean ± SD | 31.5 ± 6.9 | 29.4 ± 5.7 | 26.4 ± 5.7 | 28.4 ± 5.4 |
| ICS use | 14 (6.3) | 6 (5.5) | 12 (9.0) | 17 (3.7) |
| Post Bronchodilator FEV1 (L), mean ± SD | 2.0 ± 0.6 | 1.4 ± 0.7 | 1.4 ± 0.8 | 2.6 ± 0.8 |
| Post Bronchodilator FEV1 (percent predicted), mean ± SD | 81.3 ± 21.2 | 54.2 ± 21.5 | 50.5 ± 23.5 | 89.5 ± 19.7 |
| Post Bronchodilator FVC (L), mean ± SD | 2.8 ± 0.8 | 2.7 ± 1.0 | 2.8 ± 1.0 | 3.5 ± 0.9 |
| Post Bronchodilator FVC (percent predicted), mean ± SD | 83.1 ± 17.1 | 76.8 ± 18.9 | 78.5 ± 21.2 | 93.0 ± 15.8 |
| GOLD class |  |  |  |  |
| PRISm | 44 (19.7) | 2 ( 1.8) | 1 ( 0.8) | 35 ( 7.6) |
| GOLD 0 | 119 (53.4) | 4 ( 3.7) | 7 ( 5.3) | 282 (61.0) |
| GOLD 1 | 16 (7.2) | 16 (14.7) | 15 (11.3) | 50 (10.8) |
| GOLD 2 | 33 (14.8) | 52 (47.7) | 40 (30.1) | 73 (15.8) |
| GOLD 3 | 10 (4.5) | 25 (22.9) | 53 (39.8) | 21 ( 4.5) |
| GOLD 4 | 1 (0.4) | 10 ( 9.2) | 17 (12.8) | 1 ( 0.2) |
| Percentage of Lung Occupied by QIA, mean ± SD | 9.4 ± 4.9 | 8.9 ± 4.3 | 3.0 ± 1.1 | 2.5 ± 1.2 |
| Percentage of Lung Occupied by Emphysema, mean ± SD | 0.8 ± 1.2 | 26.1 ± 18.2 | 31.1 ± 20.7 | 0.7 ± 1.0 |

**Table S3:** Baseline characteristic of COPDGene cohort of smokers with CT measurements and exam data, with (our study cohort) and without metabolomics data collected.

|  | N = 928 | N = 3755 |
| --- | --- | --- |
| Age, mean ± SD | 67.5 ± 8.6 | 64.9 ± 8.6 |
| Male | 466 (50.2) | 1788 (47.6) |
| Self-reported race |  |  |
| White | 850 (91.6) | 2439 (65.0) |
| Black | 78 (8.4) | 1316 (35.0) |
| Former Smoker | 688 (74.1) | 2159 (57.5) |
| Pack Years, mean ± SD | 44.5 ± 24.3 | 43.7 ± 23.8 |
| Body Mass Index (kg/m2), mean ± SD | 29.0 ± 6.1 | 29.0 ± 6.4 |
| Inhaled corticosteroid use | 49 (5.3) | 174 (4.6) |
| Post Bronchodilator FEV1 (L), mean ± SD | 2.0 ± 0.9 | 2.0 ± 0.8 |
| Post Bronchodilator FEV1 (percent predicted), mean ± SD | 77.8 ± 26.0 | 78.0 ± 23.9 |
| Post Bronchodilator FVC (L), mean ± SD | 3.1 ± 1.0 | 3.0 ± 0.9 |
| Post Bronchodilator FVC (percent predicted), mean ± SD | 86.6 ± 18.5 | 86.8 ± 17.5 |
| GOLD class |  |  |
| PRISm (reduced FEV1 and FVC, with a FEV1-to-FVC ratio of ≥0.7) | 82 (8.8) | 501 (13.4) |
| GOLD 0 | 412 (44.4) | 1788 (47.9) |
| GOLD 1 | 97 (10.5) | 316 (8.5) |
| GOLD 2 | 198 (21.4) | 711 (19.0) |
| GOLD 3 | 109 (11.8) | 346 (9.3) |
| GOLD 4 | 29 (3.1) | 74 (2.0) |
| Percentage of Lung Occupied by QIA, mean ± SD | 5.0 ± 4.3 | 5.9 ± 4.7 |
| ≥ 5% Percentage of Lung Occupied by QIA | 332 (35.8) | 1703 (45.4) |
| Percentage of Lung Occupied by Emphysema, mean ± SD | 8.1 ± 16.0 | 5.7 ± 12.7 |
| ≥ 5% Percentage of Lung Occupied by Emphysema | 242 (26.1) | 769 (20.5) |

**Table S4:** Univariate regression of each metabolite with continuous percent QIA

| **Metabolite** | **HMDB ID** | **Percent QIA per unit metabolite, mean [CI]** | **p** | **FDR p** | **Metabolon class** | **Metabolon sub-class** |
| --- | --- | --- | --- | --- | --- | --- |
| N-acetylalanine | 0000766 | 2.68 [1.75 to 3.62] | < 0.001 | < 0.001 | Amino Acid | Alanine and Aspartate Metabolism |
| Hydroxyasparagine | 32332 | 2.68 [2.09 to 3.26] | < 0.001 | < 0.001 | Amino Acid | Alanine and Aspartate Metabolism |
| C-glycosyltryptophan | 0240296 | 2.64 [2.04 to 3.25] | < 0.001 | < 0.001 | Amino Acid | Tryptophan Metabolism |
| Pseudouridine | 0000767 | 2.62 [1.88 to 3.37] | < 0.001 | < 0.001 | Nucleotide | Pyrimidine Metabolism, Uracil containing |
| N-acetylneuraminate | 0000230 | 2.45 [1.83 to 3.06] | < 0.001 | < 0.001 | Carbohydrate | Aminosugar Metabolism |
| 5-6-dihydrouridine | 0000497 | 2.38 [1.72 to 3.04] | < 0.001 | < 0.001 | Nucleotide | Pyrimidine Metabolism, Uracil containing |
| Erythronate | 0000613 | 2.34 [1.70 to 2.98] | < 0.001 | < 0.001 | Carbohydrate | Aminosugar Metabolism |
| Dimethylarginine-sdma-adma | 0003334,0001539 | 2.29 [1.25 to 3.34] | < 0.001 | < 0.001 | Amino Acid | Urea cycle; Arginine and Proline Metabolism |
| N-acetylserine | 0002931 | 2.21 [1.52 to 2.91] | < 0.001 | < 0.001 | Amino Acid | Glycine, Serine and Threonine Metabolism |
| N-2-n-2-dimethylguanosine | 0004824 | 2.16 [1.51 to 2.81] | < 0.001 | < 0.001 | Nucleotide | Purine Metabolism, Guanine containing |
| N-formylmethionine | 0001015 | 2.12 [1.28 to 2.96] | < 0.001 | < 0.001 | Amino Acid | Methionine, Cysteine, SAM and Taurine Metabolism |
| Aconitate-cis-or-trans | 0000958,000072 | 2.08 [1.35 to 2.81] | < 0.001 | < 0.001 | Energy | TCA Cycle |
| 2-3-dihydroxy-5-methylthio-4-pentenoate-dmtpa | 0240388 | 2.08 [1.43 to 2.73] | < 0.001 | < 0.001 | Amino Acid | Methionine, Cysteine, SAM and Taurine Metabolism |
| 5-methylthioadenosine-mta | 0001173 | 2.02 [1.37 to 2.67] | < 0.001 | < 0.001 | Amino Acid | Polyamine Metabolism |
| Gamma-carboxyglutamate | 0041900 | 1.93 [1.33 to 2.53] | < 0.001 | < 0.001 | Amino Acid | Glutamate Metabolism |
| N-acetylmethionine | 0011745 | 1.91 [1.31 to 2.50] | < 0.001 | < 0.001 | Amino Acid | Methionine, Cysteine, SAM and Taurine Metabolism |
| Sulfate | 01448 | 1.83 [0.94 to 2.72] | < 0.001 | < 0.001 | Xenobiotics | Chemical |
| N-acetylglucosamine-n-acetylgalactosamine | 0000212,0000215 | 1.82 [1.26 to 2.38] | < 0.001 | < 0.001 | Carbohydrate | Aminosugar Metabolism |
| N-1-methyladenosine | 0003331 | 1.73 [0.80 to 2.67] | < 0.001 | 0.002 | Nucleotide | Purine Metabolism, Adenine containing |
| N-acetylthreonine | 0062557 | 1.68 [1.05 to 2.31] | < 0.001 | < 0.001 | Amino Acid | Glycine, Serine and Threonine Metabolism |
| Kynurenine | 0000684 | 1.67 [0.96 to 2.38] | < 0.001 | < 0.001 | Amino Acid | Tryptophan Metabolism |
| Phenylalanine | 0000159 | 1.63 [0.45 to 2.81] | 0.007 | 0.027 | Amino Acid | Phenylalanine Metabolism |
| N-6-carbamoylthreonyladenosine | 0041623 | 1.62 [1.08 to 2.15] | < 0.001 | < 0.001 | Nucleotide | Purine Metabolism, Adenine containing |
| 1-methyl-4-imidazoleacetate | 0002820 | 1.55 [1.06 to 2.03] | < 0.001 | < 0.001 | Amino Acid | Histidine Metabolism |
| N-4-acetylcytidine | 0005923 | 1.39 [0.97 to 1.82] | < 0.001 | < 0.001 | Nucleotide | Pyrimidine Metabolism, Cytidine containing |
| Gamma-glutamylphenylalanine | 0000594 | 1.38 [0.77 to 1.99] | < 0.001 | < 0.001 | Peptide | Gamma-glutamyl Amino Acid |
| 5-galactosylhydroxy-lysine |  | 1.38 [1.00 to 1.76] | < 0.001 | < 0.001 | Amino Acid | Lysine Metabolism |
| 4-acetamidobutanoate | 0003681 | 1.35 [0.86 to 1.84] | < 0.001 | < 0.001 | Amino Acid | Polyamine Metabolism |
| Succinylcarnitine-c-4-dc | 0061717 | 1.35 [0.85 to 1.85] | < 0.001 | < 0.001 | Energy | TCA Cycle |
| N-6-acetyllysine | 0000206 | 1.30 [0.66 to 1.93] | < 0.001 | < 0.001 | Amino Acid | Lysine Metabolism |
| N-acetylputrescine | 0002064 | 1.29 [0.64 to 1.93] | < 0.001 | < 0.001 | Amino Acid | Polyamine Metabolism |
| Cystine | 0000192 | 1.28 [0.64 to 1.91] | < 0.001 | < 0.001 | Amino Acid | Methionine, Cysteine, SAM and Taurine Metabolism |
| Mannonate |  | 1.26 [0.81 to 1.72] | < 0.001 | < 0.001 | Xenobiotics | Food Component/Plant |
| Quinolinate | 0000232 | 1.25 [0.91 to 1.60] | < 0.001 | < 0.001 | Cofactors and Vitamins | Nicotinate and Nicotinamide Metabolism |
| 2-keto-3-deoxy-gluconate | 0001353 | 1.25 [0.74 to 1.75] | < 0.001 | < 0.001 | Xenobiotics | Food Component/Plant |
| Sphingomyelin-d-18-1-18-1-d-18-2-18-0 | 0012101 | 1.24 [0.50 to 1.99] | 0.001 | 0.006 | Lipid | Sphingomyelins |
| Cytidine | 0000089 | 1.21 [0.67 to 1.75] | < 0.001 | < 0.001 | Nucleotide | Pyrimidine Metabolism, Cytidine containing |
| O-sulfo-tyrosine | 0155722 | 1.19 [0.59 to 1.79] | < 0.001 | < 0.001 | Xenobiotics | Chemical |
| N-acetylvaline | 0011757 | 1.18 [0.39 to 1.97] | 0.003 | 0.015 | Amino Acid | Leucine, Isoleucine and Valine Metabolism |
| Gulonate | 0003290 | 1.12 [0.68 to 1.57] | < 0.001 | < 0.001 | Cofactors and Vitamins | Ascorbate and Aldarate Metabolism |
| Acisoga | 0061384 | 1.12 [0.74 to 1.51] | < 0.001 | < 0.001 | Amino Acid | Polyamine Metabolism |
| Arabonate-xylonate | 0000539 | 1.10 [0.63 to 1.57] | < 0.001 | < 0.001 | Carbohydrate | Pentose Metabolism |
| N-acetyl-isoputreanine |  | 1.09 [0.64 to 1.55] | < 0.001 | < 0.001 | Amino Acid | Polyamine Metabolism |
| Sphingosine | 0000252 | 1.06 [0.49 to 1.63] | < 0.001 | 0.002 | Lipid | Sphingosines |
| Dimethylglycine | 0000092 | 1.05 [0.43 to 1.67] | < 0.001 | 0.006 | Amino Acid | Glycine, Serine and Threonine Metabolism |
| Stearoyl-sphingomyelin-d-18-1-18-0 | 0001348 | 1.04 [0.30 to 1.79] | 0.006 | 0.024 | Lipid | Sphingomyelins |
| Mannose | 0000169 | 1.00 [0.37 to 1.62] | 0.002 | 0.008 | Carbohydrate | Fructose, Mannose and Galactose Metabolism |
| Adenine | 0000034 | 0.99 [0.43 to 1.56] | < 0.001 | 0.004 | Nucleotide | Purine Metabolism, Adenine containing |
| Methionine-sulfone | 0062174 | 0.97 [0.56 to 1.39] | < 0.001 | < 0.001 | Amino Acid | Methionine, Cysteine, SAM and Taurine Metabolism |
| 2-aminoheptanoate | 0094649 | 0.96 [0.49 to 1.43] | < 0.001 | < 0.001 | Lipid | Fatty Acid, Amino |
| Phenylacetylglutamine | 0006344 | 0.95 [0.62 to 1.27] | < 0.001 | < 0.001 | Peptide | Acetylated Peptides |
| Sphingomyelin-d-18-1-20-2-d-18-2-20-1-d-16-1-22-2 |  | 0.95 [0.45 to 1.45] | < 0.001 | 0.001 | Lipid | Sphingomyelins |
| Beta-citrylglutamate |  | 0.93 [0.40 to 1.46] | < 0.001 | 0.003 | Amino Acid | Glutamate Metabolism |
| Ribonate | 0000867 | 0.92 [0.43 to 1.41] | < 0.001 | 0.002 | Carbohydrate | Pentose Metabolism |
| Alpha-ketoglutaramate | 0001552 | 0.91 [0.31 to 1.50] | 0.003 | 0.013 | Amino Acid | Glutamate Metabolism |
| 1-ribosyl-imidazoleacetate | 0002331 | 0.90 [0.48 to 1.33] | < 0.001 | < 0.001 | Amino Acid | Histidine Metabolism |
| N-n-n-trimethyl-5-aminovalerate |  | 0.90 [0.47 to 1.34] | < 0.001 | < 0.001 | Amino Acid | Lysine Metabolism |
| 1-methylhistidine | 0000001 | 0.87 [0.39 to 1.35] | < 0.001 | 0.003 | Amino Acid | Histidine Metabolism |
| Sphingomyelin-d-18-1-22-2-d-18-2-22-1-d-16-1-24-2 | 0240670,0240672,0240669 | 0.87 [0.24 to 1.51] | 0.007 | 0.027 | Lipid | Sphingomyelins |
| N-acetylglutamate | 0001138 | 0.86 [0.39 to 1.33] | < 0.001 | 0.002 | Amino Acid | Glutamate Metabolism |
| Arabitol-xylitol | 0001851,0000568,0002917 | 0.85 [0.28 to 1.41] | 0.004 | 0.016 | Carbohydrate | Pentose Metabolism |
| Dimethylguanidino-valeric-acid-dmgv |  | 0.85 [0.60 to 1.09] | < 0.001 | < 0.001 | Amino Acid | Urea cycle; Arginine and Proline Metabolism |
| 3-hydroxyadipate | 0000345 | 0.83 [0.50 to 1.16] | < 0.001 | < 0.001 | Lipid | Fatty Acid, Dicarboxylate |
| 1-carboxyethyltyrosine |  | 0.83 [0.52 to 1.14] | < 0.001 | < 0.001 | Amino Acid | Tyrosine Metabolism |
| Vanillylmandelate-vma | 0000291 | 0.82 [0.38 to 1.26] | < 0.001 | 0.002 | Amino Acid | Tyrosine Metabolism |
| 1-carboxyethylphenylalanine |  | 0.82 [0.50 to 1.15] | < 0.001 | < 0.001 | Amino Acid | Phenylalanine Metabolism |
| 3-hydroxy-3-methylglutarate | 0000355 | 0.79 [0.42 to 1.17] | < 0.001 | < 0.001 | Lipid | Mevalonate Metabolism |
| N-6-carboxymethyllysine | 0240347 | 0.79 [0.31 to 1.28] | 0.001 | 0.007 | Carbohydrate | Advanced Glycation End-product |
| Adipoylcarnitine-c-6-dc | 0061677 | 0.79 [0.44 to 1.14] | < 0.001 | < 0.001 | Lipid | Fatty Acid Metabolism (Acyl Carnitine, Dicarboxylate) |
| Trimethylamine-n-oxide | 0000925 | 0.78 [0.46 to 1.09] | < 0.001 | < 0.001 | Lipid | Phospholipid Metabolism |
| Maltose | 0000163 | 0.77 [0.53 to 1.02] | < 0.001 | < 0.001 | Carbohydrate | Glycogen Metabolism |
| S-3-hydroxybutyrylcarnitine | 0013127 | 0.76 [0.42 to 1.09] | < 0.001 | < 0.001 | Lipid | Fatty Acid Metabolism (Acyl Carnitine, Hydroxy) |
| 2-methylmalonylcarnitine-c-4-dc | 0013133 | 0.73 [0.33 to 1.13] | < 0.001 | 0.002 | Lipid | Fatty Acid Metabolism (also BCAA Metabolism) |
| N-acetyltaurine | 0240253 | 0.73 [0.32 to 1.15] | < 0.001 | 0.003 | Amino Acid | Methionine, Cysteine, SAM and Taurine Metabolism |
| Sphingadienine |  | 0.73 [0.15 to 1.32] | 0.014 | 0.048 | Lipid | Sphingolipid Synthesis |
| 1-carboxyethylvaline |  | 0.72 [0.38 to 1.05] | < 0.001 | < 0.001 | Amino Acid | Leucine, Isoleucine and Valine Metabolism |
| Sphinganine | 0000269 | 0.71 [0.16 to 1.27] | 0.012 | 0.043 | Lipid | Sphingolipid Synthesis |
| N-1-methylinosine | 0002721 | 0.71 [0.32 to 1.10] | < 0.001 | 0.002 | Nucleotide | Purine Metabolism, (Hypo)Xanthine/Inosine containing |
| Gluconate | 0000625 | 0.70 [0.38 to 1.03] | < 0.001 | < 0.001 | Xenobiotics | Food Component/Plant |
| 5-hydroxylysine | 0000450 | 0.70 [0.33 to 1.07] | < 0.001 | 0.002 | Amino Acid | Lysine Metabolism |
| 3-ureidopropionate | 0000026 | 0.69 [0.32 to 1.07] | < 0.001 | 0.002 | Nucleotide | Pyrimidine Metabolism, Uracil containing |
| 3-methylglutarylcarnitine-2 | 0000552 | 0.69 [0.46 to 0.92] | < 0.001 | < 0.001 | Amino Acid | Leucine, Isoleucine and Valine Metabolism |
| Homocitrulline | 0000679 | 0.68 [0.37 to 0.99] | < 0.001 | < 0.001 | Amino Acid | Urea cycle; Arginine and Proline Metabolism |
| 3-methoxytyrosine | 0001434 | 0.66 [0.28 to 1.04] | < 0.001 | 0.005 | Amino Acid | Tyrosine Metabolism |
| Glycine-conjugate-of-c-10-h-12-o-2 |  | 0.66 [0.33 to 1.00] | < 0.001 | < 0.001 | Partially Characterized Molecules | Partially Characterized Molecules |
| Glutamate-gamma-methyl-ester | 0061715 | 0.65 [0.15 to 1.15] | 0.010 | 0.038 | Amino Acid | Glutamate Metabolism |
| Arabinose | 0029942 | 0.64 [0.20 to 1.09] | 0.005 | 0.020 | Carbohydrate | Pentose Metabolism |
| 6-oxopiperidine-2-carboxylate | 0061705 | 0.64 [0.20 to 1.07] | 0.004 | 0.018 | Amino Acid | Lysine Metabolism |
| Ylose | 0000098 | 0.63 [0.32 to 0.93] | < 0.001 | < 0.001 | Carbohydrate | Pentose Metabolism |
| Gamma-cehc-glucuronide |  | 0.63 [0.36 to 0.90] | < 0.001 | < 0.001 | Cofactors and Vitamins | Tocopherol Metabolism |
| Methyl-indole-3-acetate | 0029738 | 0.61 [0.27 to 0.96] | < 0.001 | 0.003 | Xenobiotics | Food Component/Plant |
| Phenylacetylglutamate | 0059772 | 0.61 [0.36 to 0.87] | < 0.001 | < 0.001 | Peptide | Acetylated Peptides |
| 1-stearoyl-2-docosahexaenoyl-gpe-18-0-22-6 | 0009012 | 0.60 [0.28 to 0.93] | < 0.001 | 0.002 | Lipid | Phosphatidylethanolamine (PE) |
| Gamma-glutamylvaline | 0011172 | 0.58 [0.22 to 0.94] | 0.002 | 0.008 | Peptide | Gamma-glutamyl Amino Acid |
| Orotidine | 0000788 | 0.58 [0.33 to 0.83] | < 0.001 | < 0.001 | Nucleotide | Pyrimidine Metabolism, Orotate containing |
| Methylmalonate-mma | 0000202 | 0.57 [0.24 to 0.91] | < 0.001 | 0.005 | Lipid | Fatty Acid Metabolism (also BCAA Metabolism) |
| Vanillactate | 0000913 | 0.57 [0.25 to 0.88] | < 0.001 | 0.003 | Amino Acid | Tyrosine Metabolism |
| 3-methylglutaconate | 0000522 | 0.56 [0.24 to 0.88] | < 0.001 | 0.004 | Amino Acid | Leucine, Isoleucine and Valine Metabolism |
| 6-hydroxyindole-sulfate | 0000682 | 0.56 [0.26 to 0.87] | < 0.001 | 0.002 | Xenobiotics | Chemical |
| 1-carboxyethylisoleucine |  | 0.56 [0.26 to 0.86] | < 0.001 | 0.002 | Amino Acid | Leucine, Isoleucine and Valine Metabolism |
| 4-hydroxyglutamate | 0001344 | 0.54 [0.26 to 0.83] | < 0.001 | 0.001 | Amino Acid | Glutamate Metabolism |
| N-methylhydroxyproline |  | 0.54 [0.21 to 0.86] | 0.001 | 0.006 | Amino Acid | Urea cycle; Arginine and Proline Metabolism |
| 3-hydroxyhexanoate | 0061652,0010718 | 0.52 [0.11 to 0.93] | 0.013 | 0.046 | Lipid | Fatty Acid, Monohydroxy |
| Valerate-5-0 | 0000892 | 0.51 [0.15 to 0.87] | 0.006 | 0.023 | Lipid | Short Chain Fatty Acid |
| Hydantoin-5-propionate | 0001212 | 0.50 [0.24 to 0.77] | < 0.001 | 0.001 | Amino Acid | Histidine Metabolism |
| Glycosyl-n-2-hydroxynervonoyl-sphingosine-d-18-1-24-1-2-oh |  | 0.50 [0.17 to 0.84] | 0.004 | 0.016 | Lipid | Hexosylceramides (HCER) |
| Cystathionine | 0000099 | 0.49 [0.20 to 0.78] | < 0.001 | 0.006 | Amino Acid | Methionine, Cysteine, SAM and Taurine Metabolism |
| N-stearoyl-sphinganine-d-18-0-18-0 | 0011761 | 0.49 [0.20 to 0.79] | 0.001 | 0.006 | Lipid | Dihydroceramides |
| 3-indoxyl-sulfate | 0000682 | 0.49 [0.19 to 0.79] | 0.001 | 0.007 | Amino Acid | Tryptophan Metabolism |
| 7-methylurate | 0011107 | 0.49 [0.20 to 0.79] | 0.001 | 0.006 | Xenobiotics | Xanthine Metabolism |
| Cis-4-decenoylcarnitine-c-10-1 | 0013205 | 0.49 [0.13 to 0.85] | 0.007 | 0.027 | Lipid | Fatty Acid Metabolism (Acyl Carnitine, Monounsaturated) |
| Glycine-conjugate-of-c-10-h-14-o-2-1 |  | 0.49 [0.20 to 0.78] | 0.001 | 0.006 | Partially Characterized Molecules | Partially Characterized Molecules |
| Phenylacetate | 0000209 | 0.48 [0.23 to 0.73] | < 0.001 | 0.001 | Amino Acid | Phenylalanine Metabolism |
| Lyxonate | 0060255 | 0.48 [0.14 to 0.82] | 0.005 | 0.021 | Carbohydrate | Pentose Metabolism |
| Heptenedioate-c-7-1-dc |  | 0.48 [0.15 to 0.82] | 0.005 | 0.020 | Lipid | Fatty Acid, Dicarboxylate |
| 1-carboxyethylleucine |  | 0.48 [0.14 to 0.82] | 0.006 | 0.023 | Amino Acid | Leucine, Isoleucine and Valine Metabolism |
| 4-hydroxyhippurate | 0013678 | 0.46 [0.20 to 0.73] | < 0.001 | 0.004 | Xenobiotics | Benzoate Metabolism |
| 1-oleoyl-2-docosahexaenoyl-gpe-18-1-22-6 | 0009078 | 0.45 [0.12 to 0.79] | 0.007 | 0.027 | Lipid | Phosphatidylethanolamine (PE) |
| Glucuronate | 0000127 | 0.44 [0.17 to 0.70] | 0.001 | 0.006 | Carbohydrate | Aminosugar Metabolism |
| 1-palmitoyl-2-docosahexaenoyl-gpe-16-0-22-6 | 0008946 | 0.44 [0.09 to 0.79] | 0.014 | 0.047 | Lipid | Phosphatidylethanolamine (PE) |
| Erythritol | 0002994 | 0.43 [0.18 to 0.69] | < 0.001 | 0.004 | Xenobiotics | Food Component/Plant |
| Indoleacetylglutamine | 0013240 | 0.42 [0.15 to 0.68] | 0.002 | 0.011 | Amino Acid | Tryptophan Metabolism |
| Sucrose | 0000258 | 0.38 [0.20 to 0.56] | < 0.001 | < 0.001 | Carbohydrate | Disaccharides and Oligosaccharides |
| Phenylacetylcarnitine |  | 0.38 [0.15 to 0.60] | 0.001 | 0.006 | Peptide | Acetylated Peptides |
| Delta-cehc |  | 0.38 [0.09 to 0.67] | 0.011 | 0.040 | Cofactors and Vitamins | Tocopherol Metabolism |
| P-cresol-sulfate | 0011635 | 0.35 [0.12 to 0.58] | 0.003 | 0.015 | Xenobiotics | Benzoate Metabolism |
| 4-methylguaiacol-sulfate |  | 0.34 [0.12 to 0.56] | 0.003 | 0.013 | Xenobiotics | Benzoate Metabolism |
| P-cresol-glucuronide | 0011686 | 0.34 [0.19 to 0.49] | < 0.001 | < 0.001 | Amino Acid | Tyrosine Metabolism |
| N-1-methyl-2-pyridone-5-carboxamide | 0004193 | 0.32 [0.07 to 0.57] | 0.012 | 0.043 | Cofactors and Vitamins | Nicotinate and Nicotinamide Metabolism |
| Taurodeoxycholate | 0000896 | 0.26 [0.08 to 0.43] | 0.003 | 0.016 | Lipid | Secondary Bile Acid Metabolism |
| 2-isopropylmalate | 0000402 | 0.26 [0.10 to 0.43] | 0.002 | 0.010 | Xenobiotics | Food Component/Plant |
| Mannitol-sorbitol | 0000247,0000765 | 0.26 [0.07 to 0.45] | 0.009 | 0.033 | Carbohydrate | Fructose, Mannose and Galactose Metabolism |
| 3-hydroxyhippurate | 0006116 | 0.26 [0.10 to 0.42] | 0.002 | 0.009 | Xenobiotics | Benzoate Metabolism |
| Taurochenodeoxycholate | 0000951 | 0.24 [0.07 to 0.41] | 0.005 | 0.022 | Lipid | Primary Bile Acid Metabolism |
| Taurocholate | 0000036 | 0.19 [0.04 to 0.34] | 0.013 | 0.045 | Lipid | Primary Bile Acid Metabolism |
| 1-2-3-benzenetriol-sulfate-2 | 0060018,0060016 | -0.22 [-0.38 to -0.06] | 0.007 | 0.028 | Xenobiotics | Chemical |
| 4-vinylphenol-sulfate | 0062775 | -0.25 [-0.44 to -0.07] | 0.006 | 0.026 | Xenobiotics | Benzoate Metabolism |
| Paraxanthine | 0001860 | -0.26 [-0.46 to -0.05] | 0.013 | 0.045 | Xenobiotics | Xanthine Metabolism |
| Androsterone-glucuronide | 0002829 | -0.26 [-0.47 to -0.05] | 0.015 | 0.050 | Lipid | Androgenic Steroids |
| 5-alpha-androstan-3-beta-17-beta-diol-disulfate | 00493 | -0.28 [-0.42 to -0.14] | < 0.001 | 0.001 | Lipid | Androgenic Steroids |
| Pregnenediol-disulfate-c-21-h-34-o-8-s-2 |  | -0.30 [-0.52 to -0.09] | 0.005 | 0.021 | Lipid | Pregnenolone Steroids |
| Androstenediol-3-beta-17-beta-disulfate-1 | 0240313 | -0.32 [-0.49 to -0.16] | < 0.001 | 0.001 | Lipid | Androgenic Steroids |
| Androsterone-sulfate | 0002759 | -0.34 [-0.51 to -0.18] | < 0.001 | < 0.001 | Lipid | Androgenic Steroids |
| Beta-cryptoxanthin | 0033844 | -0.34 [-0.57 to -0.10] | 0.006 | 0.023 | Cofactors and Vitamins | Vitamin A Metabolism |
| Pregnenolone-sulfate | 0000774 | -0.35 [-0.59 to -0.11] | 0.005 | 0.019 | Lipid | Pregnenolone Steroids |
| 5-alpha-androstan-3-alpha-17-beta-diol-monosulfate-1 |  | -0.36 [-0.58 to -0.13] | 0.002 | 0.011 | Lipid | Androgenic Steroids |
| Androstenediol-3-beta-17-beta-disulfate-2 | 0240313 | -0.37 [-0.60 to -0.14] | 0.001 | 0.007 | Lipid | Androgenic Steroids |
| Tryptophan-betaine | 0061115 | -0.38 [-0.55 to -0.21] | < 0.001 | < 0.001 | Amino Acid | Tryptophan Metabolism |
| 21-hydroxypregnenolone-disulfate |  | -0.38 [-0.63 to -0.12] | 0.004 | 0.019 | Lipid | Pregnenolone Steroids |
| Carotene-diol-3 |  | -0.39 [-0.66 to -0.11] | 0.007 | 0.027 | Cofactors and Vitamins | Vitamin A Metabolism |
| Sulfate-of-piperine-metabolite-c-18-h-21-no-3-1 |  | -0.39 [-0.62 to -0.16] | < 0.001 | 0.005 | Xenobiotics | Food Component/Plant |
| Sulfate-of-piperine-metabolite-c-18-h-21-no-3-3 |  | -0.40 [-0.65 to -0.15] | 0.002 | 0.008 | Xenobiotics | Food Component/Plant |
| Phytanate | 0000801 | -0.41 [-0.72 to -0.10] | 0.010 | 0.037 | Xenobiotics | Food Component/Plant |
| Epiandrosterone-sulfate | 0062657 | -0.43 [-0.61 to -0.26] | < 0.001 | < 0.001 | Lipid | Androgenic Steroids |
| 1-5-anhydroglucitol-1-5-ag | 0002712 | -0.44 [-0.77 to -0.12] | 0.008 | 0.029 | Carbohydrate | Glycolysis, Gluconeogenesis, and Pyruvate Metabolism |
| Androstenediol-3-beta-17-beta-monosulfate-2 | 0240429,0186954 | -0.44 [-0.66 to -0.22] | < 0.001 | < 0.001 | Lipid | Androgenic Steroids |
| Androstenediol-3-beta-17-beta-monosulfate-1 | 0240429 | -0.45 [-0.65 to -0.25] | < 0.001 | < 0.001 | Lipid | Androgenic Steroids |
| 1-linoleoyl-2-linolenoyl-gpc-18-2-18-3 | 0008141 | -0.45 [-0.74 to -0.16] | 0.002 | 0.011 | Lipid | Phosphatidylcholine (PC) |
| Androstenediol-3-alpha-17-alpha-monosulfate-3 |  | -0.47 [-0.67 to -0.26] | < 0.001 | < 0.001 | Lipid | Androgenic Steroids |
| 4-hydroxychlorothalonil | 0240624 | -0.47 [-0.80 to -0.14] | 0.005 | 0.020 | Xenobiotics | Chemical |
| 1-myristoyl-2-arachidonoyl-gpc-14-0-20-4 | 0007883 | -0.49 [-0.81 to -0.16] | 0.003 | 0.015 | Lipid | Phosphatidylcholine (PC) |
| Dehydroepiandrosterone-sulfate-dhea-s | 0001032 | -0.57 [-0.79 to -0.36] | < 0.001 | < 0.001 | Lipid | Androgenic Steroids |
| Eicosanedioate-c-20-dc |  | -0.58 [-0.97 to -0.18] | 0.005 | 0.019 | Lipid | Fatty Acid, Dicarboxylate |
| Glycosyl-n-behenoyl-sphingadienine-d-18-2-22-0 |  | -0.58 [-1.04 to -0.13] | 0.012 | 0.043 | Lipid | Hexosylceramides (HCER) |
| Glycerophosphorylcholine-gpc | 0000086 | -0.60 [-1.06 to -0.14] | 0.011 | 0.041 | Lipid | Phospholipid Metabolism |
| Cortisone | 0002802 | -0.62 [-1.05 to -0.20] | 0.004 | 0.018 | Lipid | Corticosteroids |
| 1-1-enyl-stearoyl-2-oleoyl-gpe-p-18-0-18-1 | 0011375 | -0.62 [-1.10 to -0.14] | 0.011 | 0.041 | Lipid | Plasmalogen |
| 2-stearoyl-gpe-18-0 | 0011129 | -0.63 [-1.09 to -0.17] | 0.008 | 0.029 | Lipid | Lysophospholipid |
| Behenoylcarnitine-c-22 | 0062468 | -0.63 [-1.02 to -0.25] | 0.001 | 0.006 | Lipid | Fatty Acid Metabolism (Acyl Carnitine, Long Chain Saturated) |
| Lignoceroylcarnitine-c-24 | 0240665 | -0.65 [-1.14 to -0.17] | 0.008 | 0.029 | Lipid | Fatty Acid Metabolism (Acyl Carnitine, Long Chain Saturated) |
| N-acetylglycine | 0000532 | -0.67 [-1.04 to -0.29] | < 0.001 | 0.003 | Amino Acid | Glycine, Serine and Threonine Metabolism |
| Pregnenediol-sulfate-c-21-h-34-o-5-s | 0000774 | -0.67 [-0.94 to -0.41] | < 0.001 | < 0.001 | Lipid | Pregnenolone Steroids |
| 1-linolenoyl-gpc-18-3 | 0010388 | -0.67 [-1.03 to -0.31] | < 0.001 | 0.002 | Lipid | Lysophospholipid |
| 1-palmitoyl-2-oleoyl-gpi-16-0-18-1 | 0009783 | -0.67 [-1.06 to -0.28] | < 0.001 | 0.004 | Lipid | Phosphatidylinositol (PI) |
| Gamma-glutamylcitrulline |  | -0.67 [-1.11 to -0.23] | 0.003 | 0.014 | Peptide | Gamma-glutamyl Amino Acid |
| 1-1-enyl-stearoyl-2-arachidonoyl-gpe-p-18-0-20-4 | 0005779 | -0.69 [-1.19 to -0.18] | 0.008 | 0.029 | Lipid | Plasmalogen |
| Alpha-hydroxyisocaproate | 0000665,00746,0000624 | -0.70 [-1.15 to -0.25] | 0.002 | 0.010 | Amino Acid | Leucine, Isoleucine and Valine Metabolism |
| 1-arachidonoyl-gpc-20-4-n-6 | 0010395 | -0.70 [-1.18 to -0.21] | 0.005 | 0.021 | Lipid | Lysophospholipid |
| Androstenediol-3-alpha-17-alpha-monosulfate-2 |  | -0.70 [-0.99 to -0.41] | < 0.001 | < 0.001 | Lipid | Androgenic Steroids |
| 1-palmitoyl-gpe-16-0 | 0011503 | -0.71 [-1.28 to -0.15] | 0.013 | 0.047 | Lipid | Lysophospholipid |
| Cys-gly-oxidized |  | -0.72 [-1.22 to -0.22] | 0.004 | 0.019 | Amino Acid | Glutathione Metabolism |
| Lactosyl-n-nervonoyl-sphingosine-d-18-1-24-1 | 0004872 | -0.73 [-1.22 to -0.23] | 0.004 | 0.018 | Lipid | Lactosylceramides (LCER) |
| Stearoylcarnitine-c-18 | 0000848 | -0.74 [-1.33 to -0.15] | 0.014 | 0.049 | Lipid | Fatty Acid Metabolism (Acyl Carnitine, Long Chain Saturated) |
| Ergothioneine | 0003045 | -0.74 [-0.99 to -0.49] | < 0.001 | < 0.001 | Xenobiotics | Food Component/Plant |
| 2-hydroxylaurate |  | -0.74 [-1.24 to -0.24] | 0.004 | 0.018 | Lipid | Fatty Acid, Monohydroxy |
| 1-palmitoleoyl-gpc-16-1 | 0010383 | -0.76 [-1.20 to -0.31] | < 0.001 | 0.005 | Lipid | Lysophospholipid |
| 1-stearoyl-2-linoleoyl-gpi-18-0-18-2 | 0009809 | -0.77 [-1.30 to -0.24] | 0.004 | 0.019 | Lipid | Phosphatidylinositol (PI) |
| Glycerol-3-phosphate | 0000126 | -0.81 [-1.37 to -0.24] | 0.005 | 0.020 | Lipid | Glycerolipid Metabolism |
| N-behenoyl-sphingadienine-d-18-2-22-0 |  | -0.81 [-1.30 to -0.32] | 0.001 | 0.007 | Lipid | Ceramides |
| 3-methyl-2-oxovalerate | 0000491 | -0.82 [-1.40 to -0.24] | 0.005 | 0.022 | Amino Acid | Leucine, Isoleucine and Valine Metabolism |
| 1-stearoyl-gpe-18-0 | 0011130 | -0.85 [-1.42 to -0.29] | 0.003 | 0.015 | Lipid | Lysophospholipid |
| 1-oleoyl-gpe-18-1 | 0011506 | -0.85 [-1.25 to -0.44] | < 0.001 | < 0.001 | Lipid | Lysophospholipid |
| 1-linoleoyl-gpe-18-2 | 0011507 | -0.88 [-1.33 to -0.43] | < 0.001 | 0.001 | Lipid | Lysophospholipid |
| Gamma-glutamylglutamine | 0011738 | -0.91 [-1.64 to -0.19] | 0.014 | 0.047 | Peptide | Gamma-glutamyl Amino Acid |
| 3-methyl-2-oxobutyrate | 0000019 | -0.92 [-1.65 to -0.20] | 0.012 | 0.043 | Amino Acid | Leucine, Isoleucine and Valine Metabolism |
| Guanidinoacetate | 0000128 | -0.96 [-1.44 to -0.48] | < 0.001 | < 0.001 | Amino Acid | Creatine Metabolism |
| 1-stearoyl-2-oleoyl-gpi-18-0-18-1 | 0240667 | -0.96 [-1.35 to -0.58] | < 0.001 | < 0.001 | Lipid | Phosphatidylinositol (PI) |
| 1-1-enyl-palmitoyl-gpc-p-16-0 | 0010407 | -0.98 [-1.41 to -0.54] | < 0.001 | < 0.001 | Lipid | Lysoplasmalogen |
| 1-1-enyl-palmitoyl-2-arachidonoyl-gpc-p-16-0-20-4 | 0011220 | -1.03 [-1.69 to -0.37] | 0.002 | 0.011 | Lipid | Plasmalogen |
| 3-formylindole | 29737 | -1.05 [-1.60 to -0.50] | < 0.001 | 0.001 | Xenobiotics | Food Component/Plant |
| 4-methyl-2-oxopentanoate | 0000695 | -1.08 [-1.66 to -0.50] | < 0.001 | 0.002 | Amino Acid | Leucine, Isoleucine and Valine Metabolism |
| Gamma-glutamyl-2-aminobutyrate |  | -1.09 [-1.55 to -0.63] | < 0.001 | < 0.001 | Peptide | Gamma-glutamyl Amino Acid |
| 2-aminobutyrate | 0000452 | -1.10 [-1.71 to -0.50] | < 0.001 | 0.002 | Amino Acid | Glutathione Metabolism |
| Retinol-vitamin-a | 0000305 | -1.11 [-1.75 to -0.48] | < 0.001 | 0.004 | Cofactors and Vitamins | Vitamin A Metabolism |
| Cholesterol | 0000067 | -1.20 [-1.93 to -0.47] | 0.001 | 0.007 | Lipid | Sterol |
| 1-lignoceroyl-gpc-24-0 | 0010405 | -1.21 [-1.64 to -0.79] | < 0.001 | < 0.001 | Lipid | Lysophospholipid |
| 1-arachidonoyl-gpe-20-4-n-6 | 0011517 | -1.25 [-1.86 to -0.64] | < 0.001 | < 0.001 | Lipid | Lysophospholipid |
| 1-1-enyl-palmitoyl-2-linoleoyl-gpc-p-16-0-18-2 | 0011211 | -1.26 [-1.88 to -0.63] | < 0.001 | < 0.001 | Lipid | Plasmalogen |
| Arachidoylcarnitine-c-20 | 0006460 | -1.39 [-1.93 to -0.85] | < 0.001 | < 0.001 | Lipid | Fatty Acid Metabolism (Acyl Carnitine, Long Chain Saturated) |
| 1-palmitoyl-2-oleoyl-gpc-16-0-18-1 | 0007972 | -1.43 [-2.41 to -0.46] | 0.004 | 0.018 | Lipid | Phosphatidylcholine (PC) |
| 1-palmitoyl-2-dihomo-linolenoyl-gpc-16-0-20-3-n-3-or-6 |  | -1.46 [-2.61 to -0.32] | 0.012 | 0.043 | Lipid | Phosphatidylcholine (PC) |
| Tryptophan | 0000929 | -1.53 [-2.39 to -0.67] | < 0.001 | 0.003 | Amino Acid | Tryptophan Metabolism |
| 5-methyluridine-ribothymidine | 0000884 | -1.60 [-2.65 to -0.56] | 0.003 | 0.013 | Nucleotide | Pyrimidine Metabolism, Uracil containing |
| 1-2-dilinoleoyl-gpc-18-2-18-2 | 0008138 | -1.64 [-2.21 to -1.07] | < 0.001 | < 0.001 | Lipid | Phosphatidylcholine (PC) |
| 2-palmitoyl-gpc-16-0 | 0061702 | -1.76 [-2.36 to -1.16] | < 0.001 | < 0.001 | Lipid | Lysophospholipid |
| 1-linoleoyl-gpc-18-2 | 0010386 | -1.77 [-2.46 to -1.08] | < 0.001 | < 0.001 | Lipid | Lysophospholipid |
| 1-oleoyl-gpc-18-1 | 0002815 | -1.83 [-2.50 to -1.17] | < 0.001 | < 0.001 | Lipid | Lysophospholipid |
| 1-linoleoyl-2-arachidonoyl-gpc-18-2-20-4-n-6 | 0008147 | -1.88 [-2.53 to -1.23] | < 0.001 | < 0.001 | Lipid | Phosphatidylcholine (PC) |
| 1-stearoyl-gpc-18-0 | 0010384 | -1.91 [-2.69 to -1.12] | < 0.001 | < 0.001 | Lipid | Lysophospholipid |
| 1-palmitoyl-2-stearoyl-gpc-16-0-18-0 | 0007970 | -2.06 [-2.98 to -1.15] | < 0.001 | < 0.001 | Lipid | Phosphatidylcholine (PC) |
| 1-stearoyl-2-linoleoyl-gpc-18-0-18-2 | 0008039 | -2.22 [-3.46 to -0.98] | < 0.001 | 0.003 | Lipid | Phosphatidylcholine (PC) |
| 1-palmitoyl-2-linoleoyl-gpc-16-0-18-2 | 0007973 | -2.63 [-4.06 to -1.21] | < 0.001 | 0.002 | Lipid | Phosphatidylcholine (PC) |
| 1-palmitoyl-gpc-16-0 | 0010382 | -2.89 [-4.00 to -1.77] | < 0.001 | < 0.001 | Lipid | Lysophospholipid |

**Table S5:** Multivariable regression of each metabolite with continuous percent QIA

| **Metabolite** | **HMDB ID** | **Percent QIA per unit metabolite, mean [CI]** | **p** | **FDR p** | **Metabolon class** | **Metabolon sub-class** |
| --- | --- | --- | --- | --- | --- | --- |
| N-acetylneuraminate | 0000230 | 1.41 [0.80 to 2.02] | < 0.001 | < 0.001 | Carbohydrate | Aminosugar Metabolism |
| Pseudouridine | 0000767 | 1.35 [0.61 to 2.09] | < 0.001 | 0.009 | Nucleotide | Pyrimidine Metabolism, Uracil containing |
| C-glycosyltryptophan | 0240296 | 1.31 [0.69 to 1.93] | < 0.001 | 0.002 | Amino Acid | Tryptophan Metabolism |
| Erythronate | 0000613 | 1.23 [0.59 to 1.87] | < 0.001 | 0.005 | Carbohydrate | Aminosugar Metabolism |
| N-acetylserine | 0002931 | 1.23 [0.55 to 1.90] | < 0.001 | 0.010 | Amino Acid | Glycine, Serine and Threonine Metabolism |
| N-acetylmethionine | 0011745 | 1.14 [0.57 to 1.72] | < 0.001 | 0.004 | Amino Acid | Methionine, Cysteine, SAM and Taurine Metabolism |
| 5-6-dihydrouridine | 0000497 | 1.11 [0.46 to 1.76] | < 0.001 | 0.015 | Nucleotide | Pyrimidine Metabolism, Uracil containing |
| N-acetylthreonine | 0062557 | 1.01 [0.40 to 1.62] | 0.001 | 0.021 | Amino Acid | Glycine, Serine and Threonine Metabolism |
| N-2-n-2-dimethylguanosine | 0004824 | 1.01 [0.35 to 1.66] | 0.003 | 0.030 | Nucleotide | Purine Metabolism, Guanine containing |
| Cytidine | 0000089 | 0.98 [0.48 to 1.48] | < 0.001 | 0.004 | Nucleotide | Pyrimidine Metabolism, Cytidine containing |
| Hydroxyasparagine | 32332 | 0.98 [0.32 to 1.64] | 0.004 | 0.038 | Amino Acid | Alanine and Aspartate Metabolism |
| Sphingosine | 0000252 | 0.94 [0.40 to 1.47] | < 0.001 | 0.013 | Lipid | Sphingosines |
| N-acetylglucosamine-n-acetylgalactosamine | 0000212,0000215 | 0.92 [0.37 to 1.47] | 0.001 | 0.018 | Carbohydrate | Aminosugar Metabolism |
| N-acetylputrescine | 0002064 | 0.89 [0.30 to 1.49] | 0.003 | 0.035 | Amino Acid | Polyamine Metabolism |
| N-4-acetylcytidine | 0005923 | 0.89 [0.49 to 1.30] | < 0.001 | 0.001 | Nucleotide | Pyrimidine Metabolism, Cytidine containing |
| N-acetyl-isoputreanine |  | 0.87 [0.44 to 1.29] | < 0.001 | 0.003 | Amino Acid | Polyamine Metabolism |
| Succinylcarnitine-c-4-dc | 0061717 | 0.85 [0.37 to 1.32] | < 0.001 | 0.012 | Energy | TCA Cycle |
| 1-methyl-4-imidazoleacetate | 0002820 | 0.81 [0.35 to 1.28] | < 0.001 | 0.013 | Amino Acid | Histidine Metabolism |
| 3-ureidopropionate | 0000026 | 0.78 [0.43 to 1.14] | < 0.001 | 0.001 | Nucleotide | Pyrimidine Metabolism, Uracil containing |
| Quinolinate | 0000232 | 0.73 [0.37 to 1.08] | < 0.001 | 0.003 | Cofactors and Vitamins | Nicotinate and Nicotinamide Metabolism |
| Acisoga | 0061384 | 0.66 [0.29 to 1.02] | < 0.001 | 0.011 | Amino Acid | Polyamine Metabolism |
| 5-galactosylhydroxy-lysine |  | 0.61 [0.23 to 0.98] | 0.002 | 0.025 | Amino Acid | Lysine Metabolism |
| N-acetyltaurine | 0240253 | 0.55 [0.16 to 0.94] | 0.005 | 0.048 | Amino Acid | Methionine, Cysteine, SAM and Taurine Metabolism |
| Phenylacetylglutamine | 0006344 | 0.54 [0.23 to 0.85] | < 0.001 | 0.015 | Peptide | Acetylated Peptides |
| 1-carboxyethyltyrosine |  | 0.51 [0.22 to 0.81] | < 0.001 | 0.015 | Amino Acid | Tyrosine Metabolism |
| Maltose | 0000163 | 0.49 [0.26 to 0.73] | < 0.001 | 0.002 | Carbohydrate | Glycogen Metabolism |
| Ylose | 0000098 | 0.47 [0.19 to 0.75] | 0.001 | 0.019 | Carbohydrate | Pentose Metabolism |
| N-methylhydroxyproline |  | 0.46 [0.16 to 0.76] | 0.002 | 0.029 | Amino Acid | Urea cycle; Arginine and Proline Metabolism |
| 1-carboxyethylphenylalanine |  | 0.45 [0.14 to 0.76] | 0.004 | 0.042 | Amino Acid | Phenylalanine Metabolism |
| Dimethylguanidino-valeric-acid-dmgv |  | 0.43 [0.18 to 0.67] | < 0.001 | 0.015 | Amino Acid | Urea cycle; Arginine and Proline Metabolism |
| Taurocholenate-sulfate |  | 0.42 [0.17 to 0.67] | < 0.001 | 0.018 | Lipid | Secondary Bile Acid Metabolism |
| Glycochenodeoxycholate-3-sulfate | 0002409,0002496,0002497 | 0.30 [0.10 to 0.50] | 0.003 | 0.035 | Lipid | Primary Bile Acid Metabolism |
| Sucrose | 0000258 | 0.27 [0.10 to 0.43] | 0.002 | 0.025 | Carbohydrate | Disaccharides and Oligosaccharides |
| Taurochenodeoxycholate | 0000951 | 0.23 [0.07 to 0.38] | 0.004 | 0.041 | Lipid | Primary Bile Acid Metabolism |
| Hydroxy-cmpf |  | -0.19 [-0.31 to -0.07] | 0.002 | 0.026 | Lipid | Fatty Acid, Dicarboxylate |
| 10-heptadecenoate-17-1-n-7 | 0060038 | -0.31 [-0.53 to -0.10] | 0.005 | 0.043 | Lipid | Long Chain Monounsaturated Fatty Acid |
| Hexadecadienoate-16-2-n-6 | 0000477 | -0.31 [-0.52 to -0.10] | 0.003 | 0.035 | Lipid | Long Chain Polyunsaturated Fatty Acid (n3 and n6) |
| Stearidonate-18-4-n-3 | 0006547 | -0.34 [-0.53 to -0.14] | < 0.001 | 0.013 | Lipid | Long Chain Polyunsaturated Fatty Acid (n3 and n6) |
| Eicosapentaenoate-epa-20-5-n-3 | 0001999 | -0.36 [-0.59 to -0.13] | 0.002 | 0.025 | Lipid | Long Chain Polyunsaturated Fatty Acid (n3 and n6) |
| 10-nonadecenoate-19-1-n-9 | 0013622 | -0.36 [-0.60 to -0.12] | 0.003 | 0.034 | Lipid | Long Chain Monounsaturated Fatty Acid |
| Docosapentaenoate-n-3-dpa-22-5-n-3 | 0006528,0001976 | -0.37 [-0.61 to -0.13] | 0.002 | 0.029 | Lipid | Long Chain Polyunsaturated Fatty Acid (n3 and n6) |
| Myristate-14-0 | 0000806 | -0.38 [-0.63 to -0.12] | 0.004 | 0.041 | Lipid | Long Chain Saturated Fatty Acid |
| Tryptophan-betaine | 0061115 | -0.38 [-0.54 to -0.22] | < 0.001 | < 0.001 | Amino Acid | Tryptophan Metabolism |
| Docosahexaenoate-dha-22-6-n-3 | 0002183 | -0.44 [-0.71 to -0.17] | 0.002 | 0.025 | Lipid | Long Chain Polyunsaturated Fatty Acid (n3 and n6) |
| 14-or-15-methylpalmitate-a-17-0-or-i-17-0 | 0061859 | -0.45 [-0.75 to -0.15] | 0.003 | 0.035 | Lipid | Fatty Acid, Branched |
| 1-linoleoyl-2-linolenoyl-gpc-18-2-18-3 | 0008141 | -0.45 [-0.73 to -0.17] | 0.002 | 0.025 | Lipid | Phosphatidylcholine (PC) |
| Alpha-ketobutyrate | 0000005 | -0.48 [-0.81 to -0.15] | 0.004 | 0.040 | Amino Acid | Methionine, Cysteine, SAM and Taurine Metabolism |
| Margarate-17-0 | 0002259 | -0.49 [-0.80 to -0.19] | 0.002 | 0.025 | Lipid | Long Chain Saturated Fatty Acid |
| 1-palmitoleoyl-2-linolenoyl-gpc-16-1-18-3 | 0008008 | -0.52 [-0.84 to -0.19] | 0.002 | 0.026 | Lipid | Phosphatidylcholine (PC) |
| Ergothioneine | 0003045 | -0.56 [-0.80 to -0.33] | < 0.001 | < 0.001 | Xenobiotics | Food Component/Plant |
| Behenoylcarnitine-c-22 | 0062468 | -0.56 [-0.91 to -0.21] | 0.002 | 0.025 | Lipid | Fatty Acid Metabolism (Acyl Carnitine, Long Chain Saturated) |
| 1-1-enyl-palmitoyl-gpc-p-16-0 | 0010407 | -0.58 [-1.00 to -0.17] | 0.005 | 0.048 | Lipid | Lysoplasmalogen |
| Ceramide-d-16-1-24-1-d-18-1-22-1 |  | -0.62 [-1.02 to -0.23] | 0.002 | 0.027 | Lipid | Ceramides |
| Gamma-glutamylcitrulline |  | -0.65 [-1.06 to -0.23] | 0.002 | 0.027 | Peptide | Gamma-glutamyl Amino Acid |
| Stearate-18-0 | 0000827 | -0.66 [-1.12 to -0.19] | 0.005 | 0.048 | Lipid | Long Chain Saturated Fatty Acid |
| Pentadecanoate-15-0 | 0000826 | -0.66 [-1.11 to -0.22] | 0.003 | 0.035 | Lipid | Long Chain Saturated Fatty Acid |
| 1-myristoyl-2-arachidonoyl-gpc-14-0-20-4 | 0007883 | -0.67 [-0.99 to -0.36] | < 0.001 | 0.002 | Lipid | Phosphatidylcholine (PC) |
| Stearoyl-ethanolamide | 0013078 | -0.76 [-1.27 to -0.25] | 0.003 | 0.035 | Lipid | Endocannabinoid |
| 3-formylindole | 29737 | -0.77 [-1.29 to -0.25] | 0.004 | 0.039 | Xenobiotics | Food Component/Plant |
| 1-arachidonoyl-gpe-20-4-n-6 | 0011517 | -0.83 [-1.40 to -0.27] | 0.004 | 0.039 | Lipid | Lysophospholipid |
| 1-lignoceroyl-gpc-24-0 | 0010405 | -0.84 [-1.25 to -0.43] | < 0.001 | 0.003 | Lipid | Lysophospholipid |
| Ceramide-d-18-1-20-0-d-16-1-22-0-d-20-1-18-0 | 0240684,0240682,0004951 | -0.91 [-1.40 to -0.41] | < 0.001 | 0.009 | Lipid | Ceramides |
| Citrulline | 0000904 | -0.92 [-1.56 to -0.29] | 0.005 | 0.043 | Amino Acid | Urea cycle; Arginine and Proline Metabolism |
| N-behenoyl-sphingadienine-d-18-2-22-0 |  | -0.93 [-1.38 to -0.47] | < 0.001 | 0.003 | Lipid | Ceramides |
| Sphingomyelin-d-17-1-14-0-d-16-1-15-0 |  | -0.95 [-1.33 to -0.57] | < 0.001 | < 0.001 | Lipid | Sphingomyelins |
| 1-2-dilinoleoyl-gpc-18-2-18-2 | 0008138 | -0.98 [-1.53 to -0.44] | < 0.001 | 0.010 | Lipid | Phosphatidylcholine (PC) |
| Arachidoylcarnitine-c-20 | 0006460 | -1.00 [-1.52 to -0.48] | < 0.001 | 0.005 | Lipid | Fatty Acid Metabolism (Acyl Carnitine, Long Chain Saturated) |
| Sphingomyelin-d-17-2-16-0-d-18-2-15-0 | 0240677 | -1.02 [-1.47 to -0.58] | < 0.001 | < 0.001 | Lipid | Sphingomyelins |
| 1-oleoyl-gpc-18-1 | 0002815 | -1.04 [-1.69 to -0.39] | 0.002 | 0.025 | Lipid | Lysophospholipid |
| 1-linoleoyl-gpc-18-2 | 0010386 | -1.04 [-1.69 to -0.39] | 0.002 | 0.025 | Lipid | Lysophospholipid |
| 3-methyl-2-oxobutyrate | 0000019 | -1.06 [-1.75 to -0.37] | 0.003 | 0.030 | Amino Acid | Leucine, Isoleucine and Valine Metabolism |
| Gamma-glutamyl-2-aminobutyrate |  | -1.06 [-1.48 to -0.64] | < 0.001 | < 0.001 | Peptide | Gamma-glutamyl Amino Acid |
| 1-stearoyl-2-arachidonoyl-gpi-18-0-20-4 | 0009815 | -1.10 [-1.80 to -0.39] | 0.002 | 0.028 | Lipid | Phosphatidylinositol (PI) |
| 2-palmitoyl-gpc-16-0 | 0061702 | -1.10 [-1.68 to -0.53] | < 0.001 | 0.005 | Lipid | Lysophospholipid |
| Thyroxine | 0000248 | -1.13 [-1.83 to -0.42] | 0.002 | 0.025 | Amino Acid | Tyrosine Metabolism |
| 2-aminobutyrate | 0000452 | -1.13 [-1.69 to -0.57] | < 0.001 | 0.003 | Amino Acid | Glutathione Metabolism |
| Sphingomyelin-d-18-2-14-0-d-18-1-14-1 | 0240637,0240612 | -1.15 [-1.63 to -0.66] | < 0.001 | < 0.001 | Lipid | Sphingomyelins |
| Retinol-vitamin-a | 0000305 | -1.16 [-1.74 to -0.57] | < 0.001 | 0.004 | Cofactors and Vitamins | Vitamin A Metabolism |
| Sphingomyelin-d-18-1-20-0-d-16-1-22-0 | 0012102 | -1.40 [-2.22 to -0.57] | < 0.001 | 0.017 | Lipid | Sphingomyelins |
| 1-stearoyl-gpc-18-0 | 0010384 | -1.45 [-2.19 to -0.70] | < 0.001 | 0.005 | Lipid | Lysophospholipid |
| 1-palmitoyl-2-stearoyl-gpc-16-0-18-0 | 0007970 | -1.45 [-2.31 to -0.59] | 0.001 | 0.018 | Lipid | Phosphatidylcholine (PC) |
| 1-linoleoyl-2-arachidonoyl-gpc-18-2-20-4-n-6 | 0008147 | -1.59 [-2.21 to -0.97] | < 0.001 | < 0.001 | Lipid | Phosphatidylcholine (PC) |
| 1-palmitoyl-gpc-16-0 | 0010382 | -1.71 [-2.76 to -0.66] | 0.001 | 0.024 | Lipid | Lysophospholipid |
| 1-palmitoyl-2-linoleoyl-gpc-16-0-18-2 | 0007973 | -1.92 [-3.25 to -0.59] | 0.005 | 0.044 | Lipid | Phosphatidylcholine (PC) |
| 1-stearoyl-2-linoleoyl-gpc-18-0-18-2 | 0008039 | -2.09 [-3.26 to -0.91] | < 0.001 | 0.013 | Lipid | Phosphatidylcholine (PC) |

Adjusted for age, sex, body mass index, smoking status, pack-years, and inhaled corticosteroid use

**Table S6:** Analysis of Variance analysis of each metabolite with CT phenotypes, arranged by Metabolon classes and sub-classes.

|  | | | | | **Tukey pairwise comparison p-values** | | | | | |
| --- | --- | --- | --- | --- | --- | --- | --- | --- | --- | --- |
| **Metabolite** | **HMDB ID** | **Metabolon class** | **Metabolon sub-class** | **FDR p** | **QIA vs both** | **QIA vs emphysema** | **QIA vs neither** | **Emphysema vs both** | **Emphysema vs neither** | **Both vs neither** |
| Aspartate | 0000191 | Amino Acid | Alanine and Aspartate Metabolism | 0.004 | 0.601 | < 0.001 | 0.946 | 0.156 | 0.001 | 0.784 |
| Asparagine | 0000168 | Amino Acid | Alanine and Aspartate Metabolism | < 0.001 | 0.206 | 0.188 | 0.141 | 1.000 | < 0.001 | < 0.001 |
| N-acetylalanine | 0000766 | Amino Acid | Alanine and Aspartate Metabolism | < 0.001 | 0.701 | 0.010 | < 0.001 | 0.336 | 0.906 | 0.039 |
| Hydroxyasparagine | 32332 | Amino Acid | Alanine and Aspartate Metabolism | < 0.001 | 0.953 | < 0.001 | < 0.001 | 0.027 | 0.326 | < 0.001 |
| Guanidinoacetate | 0000128 | Amino Acid | Creatine Metabolism | 0.004 | 0.683 | 0.073 | < 0.001 | 0.718 | 0.930 | 0.256 |
| Creatine | 0000064 | Amino Acid | Creatine Metabolism | 0.004 | 0.025 | 0.001 | 0.285 | 0.945 | 0.046 | 0.306 |
| Glutamate | 0000148 | Amino Acid | Glutamate Metabolism | 0.001 | 0.563 | < 0.001 | 0.103 | 0.049 | 0.013 | 0.989 |
| N-acetylglutamate | 0001138 | Amino Acid | Glutamate Metabolism | 0.035 | 0.996 | 0.220 | 0.017 | 0.480 | 0.990 | 0.182 |
| 4-hydroxyglutamate | 0001344 | Amino Acid | Glutamate Metabolism | < 0.001 | 0.838 | < 0.001 | 0.008 | < 0.001 | 0.002 | 0.415 |
| Gamma-carboxyglutamate | 0041900 | Amino Acid | Glutamate Metabolism | < 0.001 | 0.629 | 0.032 | < 0.001 | 0.602 | 0.673 | 0.054 |
| Cysteine-glutathione-disulfide | 0000656 | Amino Acid | Glutathione Metabolism | 0.043 | 0.875 | 0.030 | 1.000 | 0.344 | 0.011 | 0.834 |
| Betaine | 0000043 | Amino Acid | Glycine, Serine and Threonine Metabolism | 0.020 | 0.004 | 0.670 | 0.793 | 0.140 | 0.960 | 0.012 |
| N-acetylthreonine | 0062557 | Amino Acid | Glycine, Serine and Threonine Metabolism | < 0.001 | 0.998 | 0.072 | < 0.001 | 0.227 | 0.923 | 0.020 |
| N-acetylserine | 0002931 | Amino Acid | Glycine, Serine and Threonine Metabolism | < 0.001 | 0.920 | 0.034 | < 0.001 | 0.312 | 0.465 | 0.003 |
| Histidine | 0000177 | Amino Acid | Histidine Metabolism | 0.007 | 0.389 | 0.267 | 0.478 | 1.000 | 0.008 | 0.024 |
| 1-methylhistidine | 0000001 | Amino Acid | Histidine Metabolism | 0.005 | 0.866 | 0.014 | 0.003 | 0.246 | 0.970 | 0.254 |
| 1-methyl-4-imidazoleacetate | 0002820 | Amino Acid | Histidine Metabolism | < 0.001 | 0.896 | 0.065 | < 0.001 | 0.463 | 0.774 | 0.040 |
| 1-ribosyl-imidazoleacetate | 0002331 | Amino Acid | Histidine Metabolism | 0.033 | 0.932 | 0.225 | 0.068 | 0.135 | 1.000 | 0.055 |
| Hydantoin-5-propionate | 0001212 | Amino Acid | Histidine Metabolism | 0.002 | 0.719 | 0.987 | 0.013 | 0.605 | 0.137 | 0.003 |
| Leucine | 0000687 | Amino Acid | Leucine, Isoleucine and Valine Metabolism | 0.020 | 0.597 | 0.010 | 0.993 | 0.434 | 0.007 | 0.650 |
| Valine | 0000883 | Amino Acid | Leucine, Isoleucine and Valine Metabolism | 0.013 | 0.935 | 0.006 | 0.996 | 0.107 | 0.003 | 0.963 |
| N-acetylvaline | 0011757 | Amino Acid | Leucine, Isoleucine and Valine Metabolism | 0.017 | 0.964 | 0.034 | 0.012 | 0.240 | 0.960 | 0.266 |
| 3-methylglutaconate | 0000522 | Amino Acid | Leucine, Isoleucine and Valine Metabolism | 0.016 | 0.942 | 0.067 | 0.007 | 0.395 | 1.000 | 0.242 |
| 3-methylglutarylcarnitine-2 | 0000552 | Amino Acid | Leucine, Isoleucine and Valine Metabolism | < 0.001 | 0.842 | 0.360 | < 0.001 | 0.143 | 0.274 | < 0.001 |
| 1-carboxyethylvaline |  | Amino Acid | Leucine, Isoleucine and Valine Metabolism | < 0.001 | 0.495 | < 0.001 | < 0.001 | 0.039 | 0.483 | 0.222 |
| 1-carboxyethylleucine |  | Amino Acid | Leucine, Isoleucine and Valine Metabolism | 0.002 | 0.802 | < 0.001 | 0.006 | 0.068 | 0.383 | 0.433 |
| 1-carboxyethylisoleucine |  | Amino Acid | Leucine, Isoleucine and Valine Metabolism | < 0.001 | 0.547 | < 0.001 | < 0.001 | 0.076 | 0.375 | 0.475 |
| N-6-n-6-n-6-trimethyllysine | 0001325 | Amino Acid | Lysine Metabolism | 0.016 | 0.030 | 0.010 | 0.250 | 0.999 | 0.207 | 0.372 |
| Lysine | 0003405 | Amino Acid | Lysine Metabolism | 0.050 | 0.919 | 0.017 | 0.966 | 0.221 | 0.020 | 0.986 |
| 5-hydroxylysine | 0000450 | Amino Acid | Lysine Metabolism | 0.002 | 0.875 | < 0.001 | 0.241 | 0.019 | 0.008 | 0.930 |
| N-6-acetyllysine | 0000206 | Amino Acid | Lysine Metabolism | 0.019 | 0.460 | 0.017 | 0.008 | 0.644 | 0.924 | 0.832 |
| 5-galactosylhydroxy-lysine |  | Amino Acid | Lysine Metabolism | < 0.001 | 0.995 | < 0.001 | < 0.001 | 0.003 | 0.867 | 0.002 |
| N-n-n-trimethyl-5-aminovalerate |  | Amino Acid | Lysine Metabolism | 0.020 | 1.000 | 0.020 | 0.061 | 0.071 | 0.657 | 0.231 |
| N-formylmethionine | 0001015 | Amino Acid | Methionine, Cysteine, SAM and Taurine Metabolism | < 0.001 | 0.928 | 0.055 | < 0.001 | 0.382 | 0.805 | 0.029 |
| Cystine | 0000192 | Amino Acid | Methionine, Cysteine, SAM and Taurine Metabolism | < 0.001 | 0.260 | < 0.001 | < 0.001 | 0.290 | 0.601 | 0.751 |
| Cystathionine | 0000099 | Amino Acid | Methionine, Cysteine, SAM and Taurine Metabolism | 0.027 | 1.000 | 0.071 | 0.028 | 0.193 | 0.976 | 0.177 |
| Hypotaurine | 0000965 | Amino Acid | Methionine, Cysteine, SAM and Taurine Metabolism | 0.001 | 0.005 | 0.107 | 0.998 | 0.681 | 0.039 | < 0.001 |
| N-acetylmethionine | 0011745 | Amino Acid | Methionine, Cysteine, SAM and Taurine Metabolism | < 0.001 | 0.992 | 0.085 | < 0.001 | 0.110 | 0.273 | < 0.001 |
| Methionine-sulfone | 0062174 | Amino Acid | Methionine, Cysteine, SAM and Taurine Metabolism | < 0.001 | 0.219 | 0.001 | < 0.001 | 0.496 | 0.901 | 0.709 |
| N-acetyltaurine | 0240253 | Amino Acid | Methionine, Cysteine, SAM and Taurine Metabolism | 0.018 | 0.931 | 0.761 | 0.025 | 0.515 | 0.594 | 0.025 |
| 2-3-dihydroxy-5-methylthio-4-pentenoate-dmtpa | 0240388 | Amino Acid | Methionine, Cysteine, SAM and Taurine Metabolism | < 0.001 | 0.968 | < 0.001 | < 0.001 | 0.011 | 0.981 | 0.004 |
| Phenylalanine | 0000159 | Amino Acid | Phenylalanine Metabolism | 0.009 | 0.788 | < 0.001 | 0.314 | 0.078 | 0.025 | 0.991 |
| Phenylacetate | 0000209 | Amino Acid | Phenylalanine Metabolism | 0.020 | 0.986 | 0.922 | 0.020 | 0.834 | 0.324 | 0.047 |
| 1-carboxyethylphenylalanine |  | Amino Acid | Phenylalanine Metabolism | < 0.001 | 0.677 | < 0.001 | < 0.001 | 0.018 | 0.555 | 0.090 |
| N-acetylputrescine | 0002064 | Amino Acid | Polyamine Metabolism | 0.022 | 0.906 | 0.359 | 0.041 | 0.189 | 0.983 | 0.029 |
| 5-methylthioadenosine-mta | 0001173 | Amino Acid | Polyamine Metabolism | < 0.001 | 0.955 | < 0.001 | < 0.001 | 0.018 | 1.000 | 0.002 |
| 4-acetamidobutanoate | 0003681 | Amino Acid | Polyamine Metabolism | 0.002 | 0.906 | 0.238 | 0.003 | 0.123 | 0.874 | 0.004 |
| Acisoga | 0061384 | Amino Acid | Polyamine Metabolism | < 0.001 | 0.991 | 0.012 | < 0.001 | 0.090 | 0.775 | 0.001 |
| N-1-n-8-acetylspermidine | 0002189,  0001276 | Amino Acid | Polyamine Metabolism | < 0.001 | 0.012 | 0.986 | 0.191 | 0.065 | 0.177 | < 0.001 |
| N-acetyl-isoputreanine |  | Amino Acid | Polyamine Metabolism | < 0.001 | 0.973 | 0.002 | < 0.001 | 0.038 | 0.910 | 0.043 |
| Tryptophan | 0000929 | Amino Acid | Tryptophan Metabolism | 0.008 | 1.000 | 0.912 | 0.028 | 0.967 | 0.013 | 0.111 |
| Kynurenine | 0000684 | Amino Acid | Tryptophan Metabolism | < 0.001 | 0.951 | 0.007 | < 0.001 | 0.008 | 1.000 | < 0.001 |
| 3-indoxyl-sulfate | 0000682 | Amino Acid | Tryptophan Metabolism | 0.014 | 0.963 | 0.005 | 0.050 | 0.081 | 0.398 | 0.474 |
| Indoleacetylglutamine | 0013240 | Amino Acid | Tryptophan Metabolism | 0.050 | 0.686 | 0.553 | 0.009 | 0.999 | 0.669 | 0.634 |
| Tryptophan-betaine | 0061115 | Amino Acid | Tryptophan Metabolism | < 0.001 | 0.976 | 0.104 | < 0.001 | 0.098 | 0.916 | 0.004 |
| C-glycosyltryptophan | 0240296 | Amino Acid | Tryptophan Metabolism | < 0.001 | 0.846 | 0.002 | < 0.001 | 0.094 | 0.183 | < 0.001 |
| Vanillylmandelate-vma | 0000291 | Amino Acid | Tyrosine Metabolism | < 0.001 | 0.043 | 0.285 | 0.043 | 0.818 | < 0.001 | < 0.001 |
| 3-methoxytyrosine | 0001434 | Amino Acid | Tyrosine Metabolism | 0.009 | 0.113 | 0.958 | 0.327 | 0.068 | 0.835 | 0.001 |
| Phenol-sulfate | 0060015 | Amino Acid | Tyrosine Metabolism | < 0.001 | 0.382 | < 0.001 | 0.374 | 0.032 | < 0.001 | 0.955 |
| Vanillactate | 0000913 | Amino Acid | Tyrosine Metabolism | 0.008 | 0.743 | 0.808 | 0.025 | 0.339 | 0.534 | 0.006 |
| P-cresol-glucuronide | 0011686 | Amino Acid | Tyrosine Metabolism | < 0.001 | 0.993 | 0.343 | < 0.001 | 0.350 | 0.508 | 0.006 |
| 1-carboxyethyltyrosine |  | Amino Acid | Tyrosine Metabolism | < 0.001 | 0.193 | < 0.001 | < 0.001 | 0.144 | 0.842 | 0.264 |
| Arginine | 0000517 | Amino Acid | Urea cycle; Arginine and Proline Metabolism | 0.001 | 0.663 | 0.774 | 0.033 | 0.996 | 0.005 | 0.005 |
| Citrulline | 0000904 | Amino Acid | Urea cycle; Arginine and Proline Metabolism | 0.042 | 0.747 | 0.026 | 0.033 | 0.457 | 0.823 | 0.758 |
| Proline | 0000162,  0003411 | Amino Acid | Urea cycle; Arginine and Proline Metabolism | 0.007 | 0.278 | < 0.001 | 0.136 | 0.332 | 0.053 | 0.989 |
| Trans-4-hydroxyproline | 0000725 | Amino Acid | Urea cycle; Arginine and Proline Metabolism | 0.014 | 0.559 | 0.002 | 0.439 | 0.220 | 0.024 | 0.992 |
| Homoarginine | 0000670 | Amino Acid | Urea cycle; Arginine and Proline Metabolism | < 0.001 | 0.368 | 0.068 | 0.179 | 0.927 | < 0.001 | 0.005 |
| Homocitrulline | 0000679 | Amino Acid | Urea cycle; Arginine and Proline Metabolism | 0.003 | 0.909 | 0.013 | 0.001 | 0.195 | 0.986 | 0.157 |
| Pro-hydroxy-pro | 0006695 | Amino Acid | Urea cycle; Arginine and Proline Metabolism | 0.019 | 0.712 | 0.004 | 0.828 | 0.218 | 0.011 | 0.956 |
| Dimethylarginine-sdma-adma | 0003334,  0001539 | Amino Acid | Urea cycle; Arginine and Proline Metabolism | 0.004 | 0.466 | < 0.001 | 0.010 | 0.227 | 0.360 | 0.853 |
| 2-oxoarginine | 0004225 | Amino Acid | Urea cycle; Arginine and Proline Metabolism | 0.023 | 0.959 | 0.033 | 0.988 | 0.030 | 0.005 | 0.991 |
| Dimethylguanidino-valeric-acid-dmgv |  | Amino Acid | Urea cycle; Arginine and Proline Metabolism | < 0.001 | 1.000 | 0.009 | < 0.001 | 0.031 | 0.737 | < 0.001 |
| N-acetylneuraminate | 0000230 | Carbohydrate | Aminosugar Metabolism | < 0.001 | 0.868 | 0.044 | < 0.001 | 0.423 | 0.194 | 0.001 |
| Erythronate | 0000613 | Carbohydrate | Aminosugar Metabolism | < 0.001 | 0.739 | < 0.001 | < 0.001 | 0.090 | 0.926 | 0.004 |
| N-acetylglucosamine-n-acetylgalactosamine | 0000212,  0000215 | Carbohydrate | Aminosugar Metabolism | < 0.001 | 0.581 | 0.030 | < 0.001 | 0.003 | 0.922 | < 0.001 |
| Sucrose | 0000258 | Carbohydrate | Disaccharides and Oligosaccharides | 0.025 | 0.910 | 0.485 | 0.006 | 0.930 | 0.661 | 0.276 |
| Mannose | 0000169 | Carbohydrate | Fructose, Mannose and Galactose Metabolism | 0.008 | 0.932 | 0.990 | 0.017 | 0.853 | 0.150 | 0.018 |
| Fructose | 0000660 | Carbohydrate | Fructose, Mannose and Galactose Metabolism | 0.036 | 0.532 | 0.006 | 0.394 | 0.396 | 0.085 | 0.993 |
| Maltose | 0000163 | Carbohydrate | Glycogen Metabolism | < 0.001 | 0.347 | 0.012 | < 0.001 | 0.687 | 0.735 | 0.102 |
| Glucose | 0000122 | Carbohydrate | Glycolysis, Gluconeogenesis, and Pyruvate Metabolism | 0.020 | 0.034 | 0.022 | 0.028 | 1.000 | 0.807 | 0.827 |
| 1-5-anhydroglucitol-1-5-ag | 0002712 | Carbohydrate | Glycolysis, Gluconeogenesis, and Pyruvate Metabolism | 0.010 | 0.278 | 0.005 | 0.008 | 0.613 | 0.702 | 0.962 |
| Ylose | 0000098 | Carbohydrate | Pentose Metabolism | 0.007 | 0.691 | < 0.001 | 0.101 | 0.099 | 0.073 | 0.947 |
| Ribonate | 0000867 | Carbohydrate | Pentose Metabolism | 0.003 | 0.270 | 0.054 | < 0.001 | 0.954 | 0.920 | 0.613 |
| Arabonate-xylonate | 0000539 | Carbohydrate | Pentose Metabolism | 0.011 | 0.788 | 0.075 | 0.002 | 0.623 | 0.994 | 0.322 |
| Lyxonate | 0060255 | Carbohydrate | Pentose Metabolism | 0.044 | 0.999 | 0.123 | 0.034 | 0.308 | 0.997 | 0.224 |
| Gulonate | 0003290 | Cofactors and Vitamins | Ascorbate and Aldarate Metabolism | < 0.001 | 0.438 | 0.002 | < 0.001 | 0.315 | 1.000 | 0.154 |
| Quinolinate | 0000232 | Cofactors and Vitamins | Nicotinate and Nicotinamide Metabolism | < 0.001 | 0.996 | < 0.001 | < 0.001 | 0.002 | 0.935 | < 0.001 |
| Gamma-cehc-glucuronide |  | Cofactors and Vitamins | Tocopherol Metabolism | 0.009 | 0.255 | 0.029 | 0.001 | 0.904 | 1.000 | 0.858 |
| Delta-cehc |  | Cofactors and Vitamins | Tocopherol Metabolism | 0.041 | 0.058 | 0.095 | 0.023 | 0.990 | 0.997 | 0.940 |
| Retinol-vitamin-a | 0000305 | Cofactors and Vitamins | Vitamin A Metabolism | 0.023 | 0.956 | 0.766 | 0.141 | 0.984 | 0.023 | 0.123 |
| Malate | 0031518,  0000156,  0000744 | Energy | TCA Cycle | 0.044 | 0.699 | 0.536 | 0.008 | 0.999 | 0.663 | 0.598 |
| Citrate | 0000094 | Energy | TCA Cycle | 0.040 | 0.859 | 0.064 | 0.961 | 0.509 | 0.009 | 0.599 |
| Aconitate-cis-or-trans | 0000958,  000072 | Energy | TCA Cycle | < 0.001 | 0.529 | 0.010 | < 0.001 | 0.487 | 0.738 | 0.039 |
| Succinylcarnitine-c-4-dc | 0061717 | Energy | TCA Cycle | 0.003 | 0.955 | 0.002 | 0.009 | 0.051 | 0.521 | 0.250 |
| Dehydroepiandrosterone-sulfate-dhea-s | 0001032 | Lipid | Androgenic Steroids | < 0.001 | 0.051 | 0.865 | < 0.001 | 0.345 | < 0.001 | < 0.001 |
| Androsterone-sulfate | 0002759 | Lipid | Androgenic Steroids | 0.004 | 0.893 | 0.999 | 0.012 | 0.874 | 0.079 | 0.010 |
| Epiandrosterone-sulfate | 0062657 | Lipid | Androgenic Steroids | < 0.001 | 0.993 | 0.842 | < 0.001 | 0.779 | 0.048 | 0.002 |
| 5-alpha-androstan-3-beta-17-beta-diol-disulfate | 00493 | Lipid | Androgenic Steroids | 0.020 | 0.851 | 0.859 | 0.005 | 1.000 | 0.244 | 0.352 |
| Androstenediol-3-beta-17-beta-disulfate-1 | 0240313 | Lipid | Androgenic Steroids | < 0.001 | 0.536 | 0.277 | 0.023 | 0.990 | < 0.001 | 0.001 |
| Androstenediol-3-beta-17-beta-disulfate-2 | 0240313 | Lipid | Androgenic Steroids | 0.005 | 0.376 | 0.483 | 0.291 | 0.995 | 0.013 | 0.010 |
| Androstenediol-3-alpha-17-alpha-monosulfate-2 |  | Lipid | Androgenic Steroids | 0.001 | 0.982 | 0.924 | < 0.001 | 0.998 | 0.051 | 0.046 |
| Androstenediol-3-alpha-17-alpha-monosulfate-3 |  | Lipid | Androgenic Steroids | 0.004 | 0.913 | 0.825 | < 0.001 | 0.999 | 0.133 | 0.133 |
| Androstenediol-3-beta-17-beta-monosulfate-1 | 0240429 | Lipid | Androgenic Steroids | < 0.001 | 0.420 | 0.638 | 0.002 | 0.981 | < 0.001 | < 0.001 |
| Androstenediol-3-beta-17-beta-monosulfate-2 | 0240429,  0186954 | Lipid | Androgenic Steroids | < 0.001 | 0.281 | 0.266 | < 0.001 | 1.000 | < 0.001 | < 0.001 |
| Androsterone-glucuronide | 0002829 | Lipid | Androgenic Steroids | 0.028 | 0.646 | 1.000 | 0.015 | 0.768 | 0.084 | 0.741 |
| N-behenoyl-sphingadienine-d-18-2-22-0 |  | Lipid | Ceramides | 0.016 | 0.637 | 0.984 | 0.073 | 0.513 | 0.374 | 0.009 |
| Cortisone | 0002802 | Lipid | Corticosteroids | < 0.001 | 0.625 | 0.936 | 0.005 | 0.388 | 0.149 | < 0.001 |
| Palmitoyl-linoleoyl-glycerol-16-0-18-2-2 | 0007103 | Lipid | Diacylglycerol | 0.003 | 0.106 | < 0.001 | 0.574 | 0.606 | 0.007 | 0.433 |
| Palmitoyl-oleoyl-glycerol-16-0-18-1-2 | 0007102 | Lipid | Diacylglycerol | 0.009 | 0.359 | 0.007 | 0.999 | 0.596 | 0.003 | 0.332 |
| Palmitoyl-arachidonoyl-glycerol-16-0-20-4-2 | 0007112 | Lipid | Diacylglycerol | 0.001 | 0.142 | 0.001 | 0.988 | 0.637 | < 0.001 | 0.155 |
| Palmitoleoyl-linoleoyl-glycerol-16-1-18-2-1 | 0007132 | Lipid | Diacylglycerol | 0.009 | 0.727 | < 0.001 | 0.540 | 0.104 | 0.010 | 0.999 |
| Diacylglycerol-14-0-18-1-16-0-16-1-1 |  | Lipid | Diacylglycerol | 0.002 | 0.182 | 0.004 | 1.000 | 0.728 | < 0.001 | 0.105 |
| Oleoyl-arachidonoyl-glycerol-18-1-20-4-2 | 0007228 | Lipid | Diacylglycerol | 0.013 | 0.216 | 0.011 | 0.984 | 0.819 | 0.009 | 0.254 |
| Diacylglycerol-16-1-18-2-2-16-0-18-3-1 |  | Lipid | Diacylglycerol | 0.010 | 0.061 | 0.049 | 1.000 | 1.000 | 0.023 | 0.034 |
| Diacylglycerol-12-0-18-1-14-0-16-1-16-0-14-1-2 | 0007012,  0007096 | Lipid | Diacylglycerol | 0.001 | 0.030 | 0.011 | 1.000 | 0.999 | 0.004 | 0.015 |
| N-stearoyl-sphinganine-d-18-0-18-0 | 0011761 | Lipid | Dihydroceramides | 0.037 | 0.982 | 0.113 | 0.078 | 0.114 | 0.957 | 0.112 |
| Octadecenedioylcarnitine-c-18-1-dc |  | Lipid | Fatty Acid Metabolism (Acyl Carnitine, Dicarboxylate) | < 0.001 | 0.491 | 0.284 | 0.126 | 0.996 | < 0.001 | 0.007 |
| Octadecanedioylcarnitine-c-18-dc |  | Lipid | Fatty Acid Metabolism (Acyl Carnitine, Dicarboxylate) | < 0.001 | 0.599 | 0.449 | 0.062 | 0.999 | 0.001 | 0.006 |
| Adipoylcarnitine-c-6-dc | 0061677 | Lipid | Fatty Acid Metabolism (Acyl Carnitine, Dicarboxylate) | < 0.001 | 0.564 | 0.974 | < 0.001 | 0.848 | < 0.001 | < 0.001 |
| Suberoylcarnitine-c-8-dc |  | Lipid | Fatty Acid Metabolism (Acyl Carnitine, Dicarboxylate) | < 0.001 | 0.477 | 0.364 | 0.071 | 1.000 | < 0.001 | 0.003 |
| S-3-hydroxybutyrylcarnitine | 0013127 | Lipid | Fatty Acid Metabolism (Acyl Carnitine, Hydroxy) | < 0.001 | 0.959 | 0.190 | < 0.001 | 0.601 | 0.259 | 0.008 |
| 3-hydroxyoleoylcarnitine |  | Lipid | Fatty Acid Metabolism (Acyl Carnitine, Hydroxy) | 0.002 | 0.767 | 0.076 | 0.339 | 0.649 | < 0.001 | 0.089 |
| Myristoylcarnitine-c-14 | 0005066 | Lipid | Fatty Acid Metabolism (Acyl Carnitine, Long Chain Saturated) | < 0.001 | 0.116 | 0.144 | 0.245 | 0.997 | < 0.001 | < 0.001 |
| Stearoylcarnitine-c-18 | 0000848 | Lipid | Fatty Acid Metabolism (Acyl Carnitine, Long Chain Saturated) | < 0.001 | 0.010 | < 0.001 | 0.290 | 0.737 | 0.002 | 0.160 |
| Arachidoylcarnitine-c-20 | 0006460 | Lipid | Fatty Acid Metabolism (Acyl Carnitine, Long Chain Saturated) | < 0.001 | 0.198 | < 0.001 | < 0.001 | 0.095 | 0.122 | 0.868 |
| Hexanoylcarnitine-c-6 | 0000756 | Lipid | Fatty Acid Metabolism (Acyl Carnitine, Medium Chain) | < 0.001 | 0.073 | 0.226 | 0.284 | 0.938 | 0.002 | < 0.001 |
| Octanoylcarnitine-c-8 | 0000791 | Lipid | Fatty Acid Metabolism (Acyl Carnitine, Medium Chain) | < 0.001 | 0.036 | 0.028 | 0.308 | 1.000 | < 0.001 | < 0.001 |
| Decanoylcarnitine-c-10 | 0000651 | Lipid | Fatty Acid Metabolism (Acyl Carnitine, Medium Chain) | < 0.001 | 0.024 | 0.007 | 0.576 | 0.998 | < 0.001 | < 0.001 |
| Laurylcarnitine-c-12 | 000225 | Lipid | Fatty Acid Metabolism (Acyl Carnitine, Medium Chain) | < 0.001 | 0.009 | 0.009 | 0.457 | 0.998 | < 0.001 | < 0.001 |
| Nonanoylcarnitine-c-9 | 0013288 | Lipid | Fatty Acid Metabolism (Acyl Carnitine, Medium Chain) | < 0.001 | 0.037 | 0.026 | 0.662 | 1.000 | < 0.001 | < 0.001 |
| Oleoylcarnitine-c-18-1 | 0005065 | Lipid | Fatty Acid Metabolism (Acyl Carnitine, Monounsaturated) | < 0.001 | 0.023 | 0.006 | 1.000 | 0.996 | 0.001 | 0.008 |
| Cis-4-decenoylcarnitine-c-10-1 | 0013205 | Lipid | Fatty Acid Metabolism (Acyl Carnitine, Monounsaturated) | < 0.001 | 0.149 | 0.062 | 0.009 | 0.997 | < 0.001 | < 0.001 |
| Myristoleoylcarnitine-c-14-1 | 0240588 | Lipid | Fatty Acid Metabolism (Acyl Carnitine, Monounsaturated) | < 0.001 | 0.004 | < 0.001 | 0.709 | 0.984 | < 0.001 | < 0.001 |
| Palmitoleoylcarnitine-c-16-1 | 0013207 | Lipid | Fatty Acid Metabolism (Acyl Carnitine, Monounsaturated) | < 0.001 | 0.038 | 0.015 | 0.392 | 0.999 | < 0.001 | < 0.001 |
| Eicosenoylcarnitine-c-20-1 |  | Lipid | Fatty Acid Metabolism (Acyl Carnitine, Monounsaturated) | < 0.001 | 0.003 | < 0.001 | 0.381 | 0.726 | < 0.001 | 0.051 |
| 5-dodecenoylcarnitine-c-12-1 | 13326 | Lipid | Fatty Acid Metabolism (Acyl Carnitine, Monounsaturated) | < 0.001 | 0.019 | 0.025 | 0.167 | 0.995 | < 0.001 | < 0.001 |
| Linoleoylcarnitine-c-18-2 | 0006469 | Lipid | Fatty Acid Metabolism (Acyl Carnitine, Polyunsaturated) | 0.002 | 0.304 | 0.059 | 0.663 | 0.946 | 0.001 | 0.028 |
| Linolenoylcarnitine-c-18-3 |  | Lipid | Fatty Acid Metabolism (Acyl Carnitine, Polyunsaturated) | 0.003 | 0.559 | 0.018 | 0.850 | 0.558 | < 0.001 | 0.169 |
| Dihomo-linoleoylcarnitine-c-20-2 |  | Lipid | Fatty Acid Metabolism (Acyl Carnitine, Polyunsaturated) | 0.037 | 0.222 | 0.108 | 0.999 | 0.998 | 0.041 | 0.119 |
| Hexanoylglutamine |  | Lipid | Fatty Acid Metabolism (Acyl Glutamine) | 0.003 | 0.864 | 0.777 | 0.039 | 1.000 | 0.006 | 0.020 |
| 2-aminoheptanoate | 0094649 | Lipid | Fatty Acid, Amino | 0.009 | 0.243 | 0.011 | 0.003 | 0.787 | 0.948 | 0.922 |
| Sebacate-c-10-dc | 0000792 | Lipid | Fatty Acid, Dicarboxylate | 0.012 | 0.695 | 0.115 | 0.650 | 0.801 | 0.003 | 0.158 |
| Octadecanedioate-c-18-dc | 0000782 | Lipid | Fatty Acid, Dicarboxylate | 0.027 | 0.784 | 0.074 | 0.898 | 0.624 | 0.006 | 0.400 |
| Eicosanedioate-c-20-dc |  | Lipid | Fatty Acid, Dicarboxylate | 0.003 | 0.949 | 0.009 | 0.036 | 0.009 | 0.580 | 0.040 |
| 3-hydroxyadipate | 0000345 | Lipid | Fatty Acid, Dicarboxylate | < 0.001 | 0.902 | 0.441 | < 0.001 | 0.917 | 0.026 | 0.004 |
| Dodecenedioate-c-12-1-dc | 0000933 | Lipid | Fatty Acid, Dicarboxylate | 0.047 | 0.374 | 0.238 | 0.915 | 0.999 | 0.043 | 0.107 |
| Heptenedioate-c-7-1-dc |  | Lipid | Fatty Acid, Dicarboxylate | < 0.001 | 0.786 | 0.651 | 0.010 | 0.999 | < 0.001 | 0.004 |
| 3-hydroxyoctanoate | 0001954 | Lipid | Fatty Acid, Monohydroxy | 0.004 | 0.821 | 1.000 | 0.021 | 0.886 | 0.067 | 0.009 |
| 3-hydroxydecanoate | 0002203 | Lipid | Fatty Acid, Monohydroxy | 0.045 | 0.780 | 0.996 | 0.163 | 0.907 | 0.206 | 0.044 |
| 3-hydroxysebacate | 0000350 | Lipid | Fatty Acid, Monohydroxy | < 0.001 | 0.999 | 0.953 | 0.004 | 0.989 | 0.004 | 0.031 |
| 3-hydroxyhexanoate | 0061652,  0010718 | Lipid | Fatty Acid, Monohydroxy | < 0.001 | 0.251 | 0.733 | 0.073 | 0.854 | 0.009 | < 0.001 |
| 2-hydroxylaurate |  | Lipid | Fatty Acid, Monohydroxy | 0.012 | 0.955 | 0.026 | 0.008 | 0.223 | 0.963 | 0.239 |
| Glycerol-3-phosphate | 0000126 | Lipid | Glycerolipid Metabolism | < 0.001 | 1.000 | 0.434 | 0.017 | 0.550 | < 0.001 | 0.121 |
| Glycosyl-n-behenoyl-sphingadienine-d-18-2-22-0 |  | Lipid | Hexosylceramides (HCER) | 0.022 | 0.962 | 0.011 | 0.055 | 0.126 | 0.530 | 0.493 |
| Glycosyl-n-2-hydroxynervonoyl-sphingosine-d-18-1-24-1-2-oh |  | Lipid | Hexosylceramides (HCER) | < 0.001 | 0.122 | 0.801 | 0.034 | 0.030 | 0.600 | < 0.001 |
| Lactosyl-n-nervonoyl-sphingosine-d-18-1-24-1 | 0004872 | Lipid | Lactosylceramides (LCER) | 0.020 | 0.910 | 0.028 | 0.014 | 0.294 | 0.933 | 0.390 |
| Tetradecadienoate-14-2 | 0000560 | Lipid | Long Chain Polyunsaturated Fatty Acid (n3 and n6) | 0.028 | 0.477 | 0.145 | 0.873 | 0.958 | 0.015 | 0.136 |
| 1-palmitoyl-gpc-16-0 | 0010382 | Lipid | Lysophospholipid | < 0.001 | 0.892 | 0.803 | 0.002 | 0.493 | 0.192 | 0.002 |
| 1-stearoyl-gpc-18-0 | 0010384 | Lipid | Lysophospholipid | 0.003 | 0.881 | 0.598 | 0.005 | 0.311 | 0.514 | 0.004 |
| 1-oleoyl-gpc-18-1 | 0002815 | Lipid | Lysophospholipid | < 0.001 | 0.997 | 0.077 | < 0.001 | 0.121 | 0.718 | 0.002 |
| 1-linoleoyl-gpc-18-2 | 0010386 | Lipid | Lysophospholipid | < 0.001 | 0.928 | 0.205 | < 0.001 | 0.121 | 0.647 | 0.001 |
| 1-stearoyl-gpe-18-0 | 0011130 | Lipid | Lysophospholipid | < 0.001 | 0.068 | 0.171 | 0.233 | 0.963 | < 0.001 | < 0.001 |
| 1-stearoyl-gpg-18-0 |  | Lipid | Lysophospholipid | 0.044 | 0.214 | 0.232 | 0.975 | 0.999 | 0.068 | 0.070 |
| 1-palmitoleoyl-gpc-16-1 | 0010383 | Lipid | Lysophospholipid | 0.010 | 0.928 | 1.000 | 0.028 | 0.961 | 0.081 | 0.027 |
| 2-palmitoyl-gpc-16-0 | 0061702 | Lipid | Lysophospholipid | < 0.001 | 0.979 | 0.328 | < 0.001 | 0.278 | 0.132 | < 0.001 |
| 1-palmitoyl-gpe-16-0 | 0011503 | Lipid | Lysophospholipid | < 0.001 | 0.141 | 0.088 | 0.353 | 1.000 | < 0.001 | 0.002 |
| 1-oleoyl-gpe-18-1 | 0011506 | Lipid | Lysophospholipid | < 0.001 | 0.112 | 0.991 | 0.007 | 0.282 | 0.014 | < 0.001 |
| 1-linoleoyl-gpe-18-2 | 0011507 | Lipid | Lysophospholipid | < 0.001 | 0.126 | 1.000 | 0.008 | 0.202 | 0.037 | < 0.001 |
| 1-arachidonoyl-gpe-20-4-n-6 | 0011517 | Lipid | Lysophospholipid | 0.006 | 0.363 | 0.999 | 0.104 | 0.527 | 0.174 | 0.002 |
| 1-palmitoyl-gpi-16-0 | 0061695 | Lipid | Lysophospholipid | 0.023 | 0.265 | 0.033 | 1.000 | 0.910 | 0.016 | 0.215 |
| 1-oleoyl-gpi-18-1 | 0061693 | Lipid | Lysophospholipid | 0.004 | 0.465 | 0.970 | 0.071 | 0.785 | 0.058 | 0.003 |
| 1-lignoceroyl-gpc-24-0 | 0010405 | Lipid | Lysophospholipid | < 0.001 | 0.493 | 0.299 | < 0.001 | 0.030 | 0.513 | < 0.001 |
| 2-stearoyl-gpe-18-0 | 0011129 | Lipid | Lysophospholipid | 0.004 | 0.281 | 0.889 | 0.158 | 0.747 | 0.056 | 0.002 |
| 1-linolenoyl-gpc-18-3 | 0010388 | Lipid | Lysophospholipid | < 0.001 | 0.212 | 0.993 | 0.008 | 0.420 | 0.017 | < 0.001 |
| 1-1-enyl-palmitoyl-gpc-p-16-0 | 0010407 | Lipid | Lysoplasmalogen | 0.034 | 1.000 | 0.375 | 0.020 | 0.495 | 0.930 | 0.106 |
| Cis-4-decenoate-10-1-n-6 | 0004980 | Lipid | Medium Chain Fatty Acid | 0.016 | 0.147 | 0.294 | 0.855 | 0.974 | 0.044 | 0.018 |
| 3-hydroxy-3-methylglutarate | 0000355 | Lipid | Mevalonate Metabolism | 0.001 | 0.826 | 0.034 | < 0.001 | 0.422 | 0.975 | 0.114 |
| 1-palmitoyl-2-linoleoyl-gpc-16-0-18-2 | 0007973 | Lipid | Phosphatidylcholine (PC) | 0.002 | 0.886 | 0.953 | 0.005 | 0.689 | 0.125 | 0.004 |
| 1-palmitoyl-2-oleoyl-gpc-16-0-18-1 | 0007972 | Lipid | Phosphatidylcholine (PC) | < 0.001 | 0.315 | 0.851 | 0.061 | 0.821 | 0.016 | < 0.001 |
| 1-myristoyl-2-palmitoyl-gpc-14-0-16-0 | 0007869 | Lipid | Phosphatidylcholine (PC) | < 0.001 | 0.104 | 0.133 | 0.232 | 0.996 | < 0.001 | < 0.001 |
| 1-2-dilinoleoyl-gpc-18-2-18-2 | 0008138 | Lipid | Phosphatidylcholine (PC) | < 0.001 | 0.834 | 0.113 | < 0.001 | 0.039 | 0.237 | < 0.001 |
| 1-stearoyl-2-oleoyl-gpc-18-0-18-1 | 0008038 | Lipid | Phosphatidylcholine (PC) | < 0.001 | 0.198 | 0.731 | 0.016 | 0.798 | 0.002 | < 0.001 |
| 1-palmitoyl-2-stearoyl-gpc-16-0-18-0 | 0007970 | Lipid | Phosphatidylcholine (PC) | 0.011 | 0.975 | 0.698 | 0.011 | 0.562 | 0.526 | 0.024 |
| 1-stearoyl-2-linoleoyl-gpc-18-0-18-2 | 0008039 | Lipid | Phosphatidylcholine (PC) | 0.003 | 0.837 | 0.879 | 0.008 | 0.507 | 0.272 | 0.005 |
| 1-palmitoyl-2-palmitoleoyl-gpc-16-0-16-1 | 0007969 | Lipid | Phosphatidylcholine (PC) | 0.001 | 0.257 | 0.058 | 0.570 | 0.965 | < 0.001 | 0.015 |
| 1-palmitoyl-2-dihomo-linolenoyl-gpc-16-0-20-3-n-3-or-6 |  | Lipid | Phosphatidylcholine (PC) | 0.006 | 0.149 | 0.748 | 0.384 | 0.713 | 0.071 | 0.002 |
| 1-linoleoyl-2-arachidonoyl-gpc-18-2-20-4-n-6 | 0008147 | Lipid | Phosphatidylcholine (PC) | < 0.001 | 0.624 | 0.727 | < 0.001 | 0.200 | 0.154 | < 0.001 |
| 1-myristoyl-2-arachidonoyl-gpc-14-0-20-4 | 0007883 | Lipid | Phosphatidylcholine (PC) | < 0.001 | 0.151 | 0.502 | 0.100 | 0.888 | 0.003 | < 0.001 |
| 1-linoleoyl-2-linolenoyl-gpc-18-2-18-3 | 0008141 | Lipid | Phosphatidylcholine (PC) | 0.003 | 0.169 | 0.999 | 0.130 | 0.205 | 0.338 | < 0.001 |
| 1-palmitoleoyl-2-linolenoyl-gpc-16-1-18-3 | 0008008 | Lipid | Phosphatidylcholine (PC) | < 0.001 | 0.026 | 0.600 | 0.362 | 0.443 | 0.032 | < 0.001 |
| 1-palmitoyl-2-oleoyl-gpe-16-0-18-1 | 0005320 | Lipid | Phosphatidylethanolamine (PE) | < 0.001 | 0.009 | < 0.001 | 0.221 | 0.098 | < 0.001 | 0.186 |
| 1-stearoyl-2-oleoyl-gpe-18-0-18-1 | 0008993 | Lipid | Phosphatidylethanolamine (PE) | < 0.001 | 0.008 | < 0.001 | 0.997 | 0.514 | < 0.001 | 0.002 |
| 1-palmitoyl-2-linoleoyl-gpe-16-0-18-2 | 0005322 | Lipid | Phosphatidylethanolamine (PE) | < 0.001 | 0.004 | < 0.001 | 0.710 | 0.225 | < 0.001 | 0.022 |
| 1-stearoyl-2-linoleoyl-gpe-18-0-18-2 | 0008994 | Lipid | Phosphatidylethanolamine (PE) | < 0.001 | 0.003 | < 0.001 | 0.911 | 0.436 | < 0.001 | 0.005 |
| 1-stearoyl-2-arachidonoyl-gpe-18-0-20-4 | 0009003 | Lipid | Phosphatidylethanolamine (PE) | < 0.001 | 0.029 | < 0.001 | 0.258 | 0.102 | < 0.001 | 0.359 |
| 1-palmitoyl-2-arachidonoyl-gpe-16-0-20-4 | 0005323 | Lipid | Phosphatidylethanolamine (PE) | < 0.001 | 0.035 | < 0.001 | 0.724 | 0.170 | < 0.001 | 0.133 |
| 1-palmitoyl-2-docosahexaenoyl-gpe-16-0-22-6 | 0008946 | Lipid | Phosphatidylethanolamine (PE) | < 0.001 | 0.126 | < 0.001 | 0.013 | 0.027 | 0.001 | 1.000 |
| 1-stearoyl-2-docosahexaenoyl-gpe-18-0-22-6 | 0009012 | Lipid | Phosphatidylethanolamine (PE) | < 0.001 | 0.121 | < 0.001 | < 0.001 | 0.007 | 0.002 | 0.922 |
| 1-oleoyl-2-linoleoyl-gpe-18-1-18-2 | 0005349 | Lipid | Phosphatidylethanolamine (PE) | < 0.001 | 0.030 | < 0.001 | 0.896 | 0.814 | < 0.001 | 0.062 |
| 1-oleoyl-2-arachidonoyl-gpe-18-1-20-4 | 0009069 | Lipid | Phosphatidylethanolamine (PE) | 0.016 | 0.220 | 0.004 | 0.725 | 0.680 | 0.021 | 0.556 |
| 1-oleoyl-2-docosahexaenoyl-gpe-18-1-22-6 | 0009078 | Lipid | Phosphatidylethanolamine (PE) | < 0.001 | 0.704 | < 0.001 | 0.011 | 0.037 | 0.109 | 0.641 |
| 1-palmitoyl-2-linoleoyl-gpi-16-0-18-2 | 0009784 | Lipid | Phosphatidylinositol (PI) | 0.001 | 0.007 | 0.050 | 1.000 | 0.879 | 0.022 | 0.002 |
| 1-stearoyl-2-arachidonoyl-gpi-18-0-20-4 | 0009815 | Lipid | Phosphatidylinositol (PI) | 0.030 | 0.119 | 0.854 | 0.754 | 0.545 | 0.317 | 0.008 |
| 1-palmitoyl-2-arachidonoyl-gpi-16-0-20-4 | 0009789 | Lipid | Phosphatidylinositol (PI) | 0.001 | 0.066 | 0.017 | 0.974 | 0.993 | 0.002 | 0.013 |
| 1-stearoyl-2-linoleoyl-gpi-18-0-18-2 | 0009809 | Lipid | Phosphatidylinositol (PI) | < 0.001 | 0.003 | 0.221 | 0.203 | 0.406 | 0.001 | < 0.001 |
| 1-palmitoyl-2-oleoyl-gpi-16-0-18-1 | 0009783 | Lipid | Phosphatidylinositol (PI) | < 0.001 | 0.026 | 0.523 | 0.023 | 0.507 | < 0.001 | < 0.001 |
| 1-stearoyl-2-oleoyl-gpi-18-0-18-1 | 0240667 | Lipid | Phosphatidylinositol (PI) | < 0.001 | 0.050 | 0.980 | < 0.001 | 0.191 | 0.002 | < 0.001 |
| Choline-phosphate | 0001565 | Lipid | Phospholipid Metabolism | < 0.001 | 0.504 | 0.048 | 0.024 | 0.784 | < 0.001 | 0.001 |
| Choline | 0000097 | Lipid | Phospholipid Metabolism | 0.050 | 0.933 | 0.011 | 0.301 | 0.158 | 0.186 | 0.905 |
| Glycerophosphoethanolamine | 0000114 | Lipid | Phospholipid Metabolism | < 0.001 | 0.328 | 0.252 | 0.222 | 1.000 | 0.002 | 0.005 |
| Trimethylamine-n-oxide | 0000925 | Lipid | Phospholipid Metabolism | < 0.001 | 1.000 | 0.004 | < 0.001 | 0.019 | 0.988 | < 0.001 |
| 1-1-enyl-palmitoyl-2-arachidonoyl-gpe-p-16-0-20-4 | 0011352 | Lipid | Plasmalogen | 0.007 | 0.382 | 0.034 | 0.922 | 0.825 | 0.002 | 0.115 |
| 1-1-enyl-palmitoyl-2-oleoyl-gpc-p-16-0-18-1 | 0007996 | Lipid | Plasmalogen | 0.012 | 0.217 | 0.004 | 0.016 | 0.655 | 0.534 | 0.999 |
| 1-1-enyl-palmitoyl-2-linoleoyl-gpc-p-16-0-18-2 | 0011211 | Lipid | Plasmalogen | 0.002 | 0.991 | 0.135 | < 0.001 | 0.400 | 0.825 | 0.036 |
| 21-hydroxypregnenolone-disulfate |  | Lipid | Pregnenolone Steroids | < 0.001 | 0.086 | 0.351 | 0.150 | 0.884 | 0.002 | < 0.001 |
| Pregnenediol-sulfate-c-21-h-34-o-5-s | 0000774 | Lipid | Pregnenolone Steroids | 0.002 | 0.983 | 0.414 | 0.002 | 0.358 | 0.565 | 0.008 |
| Pregnenolone-sulfate | 0000774 | Lipid | Pregnenolone Steroids | 0.012 | 0.998 | 0.999 | 0.020 | 1.000 | 0.054 | 0.077 |
| Lithocholate-sulfate-1 |  | Lipid | Secondary Bile Acid Metabolism | 0.013 | 0.334 | 1.000 | 0.180 | 0.475 | 0.282 | 0.004 |
| Sphingadienine |  | Lipid | Sphingolipid Synthesis | 0.050 | 0.037 | 0.110 | 0.056 | 0.956 | 0.976 | 0.746 |
| Stearoyl-sphingomyelin-d-18-1-18-0 | 0001348 | Lipid | Sphingomyelins | 0.003 | 0.081 | 0.438 | 0.567 | 0.812 | 0.032 | 0.002 |
| Sphingomyelin-d-18-1-18-1-d-18-2-18-0 | 0012101 | Lipid | Sphingomyelins | 0.028 | 0.843 | 0.997 | 0.097 | 0.939 | 0.143 | 0.038 |
| Sphingomyelin-d-18-1-20-2-d-18-2-20-1-d-16-1-22-2 |  | Lipid | Sphingomyelins | 0.010 | 1.000 | 0.428 | 0.006 | 0.522 | 0.725 | 0.044 |
| Sphingomyelin-d-18-1-17-0-d-17-1-18-0-d-19-1-16-0 | 0240620,  0240609,  0240622 | Lipid | Sphingomyelins | 0.010 | 0.256 | 0.225 | 0.700 | 1.000 | 0.014 | 0.023 |
| Sphingomyelin-d-18-2-18-1 | 0001348 | Lipid | Sphingomyelins | 0.008 | 0.726 | 0.328 | 0.286 | 0.961 | 0.005 | 0.060 |
| Sphingosine | 0000252 | Lipid | Sphingosines | 0.034 | 0.868 | 0.026 | 0.030 | 0.334 | 0.840 | 0.580 |
| Cholesterol | 0000067 | Lipid | Sterol | 0.003 | 0.216 | 0.999 | 0.119 | 0.354 | 0.200 | < 0.001 |
| 3-beta-hydroxy-5-cholestenoate | 0012453 | Lipid | Sterol | 0.020 | 0.942 | 1.000 | 0.041 | 0.938 | 0.153 | 0.041 |
| N-1-methylinosine | 0002721 | Nucleotide | Purine Metabolism, (Hypo)Xanthine/Inosine containing | 0.008 | 0.827 | 0.548 | 0.019 | 0.230 | 0.786 | 0.009 |
| Adenosine-5-monophosphate-amp | 0000045 | Nucleotide | Purine Metabolism, Adenine containing | 0.041 | 0.820 | 0.339 | 0.565 | 0.922 | 0.018 | 0.208 |
| N-1-methyladenosine | 0003331 | Nucleotide | Purine Metabolism, Adenine containing | 0.011 | 1.000 | 0.144 | 0.007 | 0.260 | 0.991 | 0.061 |
| N-6-carbamoylthreonyladenosine | 0041623 | Nucleotide | Purine Metabolism, Adenine containing | < 0.001 | 0.857 | 0.049 | < 0.001 | 0.455 | 0.453 | 0.009 |
| N-2-n-2-dimethylguanosine | 0004824 | Nucleotide | Purine Metabolism, Guanine containing | < 0.001 | 0.963 | 0.294 | < 0.001 | 0.216 | 0.050 | < 0.001 |
| N-4-acetylcytidine | 0005923 | Nucleotide | Pyrimidine Metabolism, Cytidine containing | < 0.001 | 0.839 | 0.167 | < 0.001 | 0.753 | 0.449 | 0.050 |
| Dihydroorotate | 03349 | Nucleotide | Pyrimidine Metabolism, Orotate containing | 0.017 | 1.000 | 0.336 | 0.008 | 0.519 | 0.868 | 0.083 |
| Orotidine | 0000788 | Nucleotide | Pyrimidine Metabolism, Orotate containing | < 0.001 | 0.977 | 0.004 | < 0.001 | 0.058 | 0.953 | 0.051 |
| Pseudouridine | 0000767 | Nucleotide | Pyrimidine Metabolism, Uracil containing | < 0.001 | 0.964 | 0.036 | < 0.001 | 0.248 | 0.543 | 0.003 |
| 3-ureidopropionate | 0000026 | Nucleotide | Pyrimidine Metabolism, Uracil containing | 0.013 | 0.967 | 0.042 | 0.053 | 0.040 | 0.841 | 0.066 |
| 5-6-dihydrouridine | 0000497 | Nucleotide | Pyrimidine Metabolism, Uracil containing | < 0.001 | 0.987 | 0.209 | < 0.001 | 0.530 | 0.129 | 0.002 |
| 5-methyluridine-ribothymidine | 0000884 | Nucleotide | Pyrimidine Metabolism, Uracil containing | 0.004 | 0.062 | 0.004 | 0.003 | 0.915 | 0.800 | 1.000 |
| Glutamine-degradant |  | Partially Characterized Molecules | Partially Characterized Molecules | 0.004 | 0.004 | 0.014 | 0.579 | 0.954 | 0.101 | 0.031 |
| Glycine-conjugate-of-c-10-h-12-o-2 |  | Partially Characterized Molecules | Partially Characterized Molecules | 0.028 | 0.895 | 0.246 | 0.007 | 0.783 | 0.925 | 0.316 |
| Glycine-conjugate-of-c-10-h-14-o-2-1 |  | Partially Characterized Molecules | Partially Characterized Molecules | 0.020 | 0.872 | 0.279 | 0.004 | 0.840 | 0.847 | 0.293 |
| Phenylacetylglutamine | 0006344 | Peptide | Acetylated Peptides | < 0.001 | 0.731 | 0.346 | < 0.001 | 0.090 | 0.506 | < 0.001 |
| Phenylacetylcarnitine |  | Peptide | Acetylated Peptides | 0.028 | 0.872 | 0.737 | 0.050 | 0.405 | 0.752 | 0.026 |
| Phenylacetylglutamate | 0059772 | Peptide | Acetylated Peptides | < 0.001 | 0.766 | 0.578 | 0.002 | 0.203 | 0.397 | < 0.001 |
| Gamma-glutamylhistidine | 0029151 | Peptide | Gamma-glutamyl Amino Acid | 0.019 | 0.136 | 0.006 | 0.590 | 0.841 | 0.046 | 0.496 |
| Gamma-glutamylphenylalanine | 0000594 | Peptide | Gamma-glutamyl Amino Acid | 0.002 | 0.482 | < 0.001 | 0.010 | 0.115 | 0.175 | 0.841 |
| Gamma-glutamylvaline | 0011172 | Peptide | Gamma-glutamyl Amino Acid | 0.002 | 0.491 | 0.001 | 0.001 | 0.252 | 0.723 | 0.580 |
| Gamma-glutamylcitrulline |  | Peptide | Gamma-glutamyl Amino Acid | 0.034 | 0.989 | 0.018 | 0.093 | 0.122 | 0.534 | 0.478 |
| 1-h-indole-7-acetic-acid |  | Xenobiotics | Bacterial/Fungal | 0.004 | 0.255 | 0.475 | 0.320 | 0.971 | 0.014 | 0.005 |
| 3-phenylpropionate-hydrocinnamate | 0000764 | Xenobiotics | Benzoate Metabolism | 0.024 | 0.088 | 0.008 | 0.109 | 0.938 | 0.334 | 0.830 |
| P-cresol-sulfate | 0011635 | Xenobiotics | Benzoate Metabolism | 0.033 | 0.464 | 0.770 | 0.210 | 0.143 | 0.949 | 0.011 |
| 4-hydroxyhippurate | 0013678 | Xenobiotics | Benzoate Metabolism | 0.004 | 0.131 | < 0.001 | 0.038 | 0.517 | 0.133 | 0.987 |
| 4-vinylphenol-sulfate | 0062775 | Xenobiotics | Benzoate Metabolism | 0.014 | 0.157 | 0.990 | 0.390 | 0.359 | 0.353 | 0.003 |
| O-cresol-sulfate | 0011635 | Xenobiotics | Benzoate Metabolism | < 0.001 | 0.070 | 0.005 | 0.948 | 0.926 | < 0.001 | 0.011 |
| 3-hydroxyhippurate | 0006116 | Xenobiotics | Benzoate Metabolism | 0.009 | 0.860 | 0.001 | 0.060 | 0.077 | 0.182 | 0.719 |
| 4-methylguaiacol-sulfate |  | Xenobiotics | Benzoate Metabolism | 0.043 | 0.934 | 0.020 | 0.074 | 0.221 | 0.615 | 0.626 |
| 3-methoxycatechol-sulfate-2 |  | Xenobiotics | Benzoate Metabolism | 0.035 | 0.742 | 0.106 | 0.921 | 0.024 | 0.012 | 0.924 |
| Edta | 0015109 | Xenobiotics | Chemical | 0.032 | 0.015 | 0.095 | 0.066 | 0.880 | 0.951 | 0.503 |
| Sulfate | 01448 | Xenobiotics | Chemical | < 0.001 | 0.380 | < 0.001 | 0.001 | 0.100 | 0.249 | 0.705 |
| Dimethyl-sulfone | 0004983 | Xenobiotics | Chemical | 0.030 | 0.245 | 0.462 | 0.764 | 0.970 | 0.072 | 0.027 |
| 4-hydroxychlorothalonil | 0240624 | Xenobiotics | Chemical | 0.013 | 0.235 | 0.005 | 0.830 | 0.665 | 0.013 | 0.488 |
| 2-methoxyresorcinol-sulfate |  | Xenobiotics | Chemical | 0.007 | 0.764 | 0.046 | 0.737 | 0.011 | 0.001 | 0.992 |
| 6-hydroxyindole-sulfate | 0000682 | Xenobiotics | Chemical | 0.012 | 0.995 | 0.010 | 0.030 | 0.074 | 0.641 | 0.250 |
| 1-2-3-benzenetriol-sulfate-2 | 0060018,  0060016 | Xenobiotics | Chemical | 0.022 | 0.882 | 0.974 | 0.009 | 0.990 | 0.138 | 0.370 |
| Perfluorooctanesulfonate-pfos | 0059586 | Xenobiotics | Chemical | < 0.001 | 0.088 | 0.029 | 0.857 | 0.996 | 0.001 | 0.008 |
| Perfluorooctanoate-pfoa | 0059587 | Xenobiotics | Chemical | 0.010 | 0.052 | 0.004 | 0.484 | 0.946 | 0.048 | 0.313 |
| 2-naphthol-sulfate |  | Xenobiotics | Chemical | 0.022 | 0.328 | 0.029 | 1.000 | 0.846 | 0.013 | 0.262 |
| Gluconate | 0000625 | Xenobiotics | Food Component/Plant | < 0.001 | 0.208 | 0.440 | < 0.001 | 0.960 | 0.170 | 0.565 |
| Homostachydrine | 0033433 | Xenobiotics | Food Component/Plant | < 0.001 | 0.003 | < 0.001 | 0.945 | 0.994 | < 0.001 | 0.004 |
| Ergothioneine | 0003045 | Xenobiotics | Food Component/Plant | < 0.001 | 0.358 | 0.623 | 0.002 | 0.968 | < 0.001 | < 0.001 |
| Cinnamoylglycine | 0011621 | Xenobiotics | Food Component/Plant | 0.031 | 0.564 | 0.007 | 0.769 | 0.395 | 0.026 | 0.902 |
| Mannonate |  | Xenobiotics | Food Component/Plant | < 0.001 | 0.848 | 0.015 | < 0.001 | 0.270 | 0.855 | 0.018 |
| 2-piperidinone | 0011749 | Xenobiotics | Food Component/Plant | 0.048 | 0.608 | 0.029 | 0.996 | 0.602 | 0.020 | 0.643 |
| 2-keto-3-deoxy-gluconate | 0001353 | Xenobiotics | Food Component/Plant | < 0.001 | 0.765 | 0.395 | 0.001 | 0.120 | 0.534 | < 0.001 |
| 3-formylindole | 29737 | Xenobiotics | Food Component/Plant | 0.005 | 0.701 | 0.984 | 0.002 | 0.907 | 0.045 | 0.381 |
| Sulfate-of-piperine-metabolite-c-18-h-21-no-3-1 |  | Xenobiotics | Food Component/Plant | 0.022 | 0.948 | 0.357 | 0.031 | 0.236 | 0.969 | 0.035 |
| Sulfate-of-piperine-metabolite-c-18-h-21-no-3-3 |  | Xenobiotics | Food Component/Plant | 0.040 | 0.938 | 0.482 | 0.056 | 0.305 | 0.958 | 0.050 |
| 2-4-or-2-5-dimethylphenol-sulfate |  | Xenobiotics | Food Component/Plant | < 0.001 | 0.040 | < 0.001 | 0.665 | 0.534 | < 0.001 | 0.171 |
| 7-methylxanthine | 0001991 | Xenobiotics | Xanthine Metabolism | 0.027 | 0.060 | 0.029 | 0.025 | 1.000 | 0.877 | 0.936 |
| 1-methylxanthine | 0010738 | Xenobiotics | Xanthine Metabolism | 0.021 | 0.092 | 0.129 | 0.004 | 0.994 | 0.981 | 1.000 |
| 7-methylurate | 0011107 | Xenobiotics | Xanthine Metabolism | 0.015 | 0.965 | 0.404 | 0.226 | 0.809 | 0.005 | 0.197 |

**Table S7:** Multinomial logistic regression of each metabolite with the CT phenotypes predominant QIA versus predominant emphysema

| **Metabolite** | **HMDB** | **Odds of QIA predominant over emphysema predominant, mean [CI]** | **p** | **FDR p** | **Metabolon class** | **Metabolon sub-class** |
| --- | --- | --- | --- | --- | --- | --- |
| Dimethylarginine-sdma-adma | 0003334,  0001539 | 5.19 [2.01-13.40] | < 0.001 | 0.021 | Amino Acid | Urea cycle; Arginine and Proline Metabolism |
| Phenylalanine | 0000159 | 5.04 [1.73-14.72] | 0.003 | 0.040 | Amino Acid | Phenylalanine Metabolism |
| Asparagine | 0000168 | 4.79 [1.99-11.54] | < 0.001 | 0.018 | Amino Acid | Alanine and Aspartate Metabolism |
| Sulfate | 01448 | 4.05 [1.79-9.17] | < 0.001 | 0.022 | Xenobiotics | Chemical |
| Proline | 0000162,  0003411 | 3.36 [1.61-7.03] | 0.001 | 0.028 | Amino Acid | Urea cycle; Arginine and Proline Metabolism |
| Kynurenine | 0000684 | 2.82 [1.48-5.34] | 0.002 | 0.031 | Amino Acid | Tryptophan Metabolism |
| Erythronate | 0000613 | 2.71 [1.46-5.04] | 0.002 | 0.031 | Carbohydrate | Aminosugar Metabolism |
| N-acetyl-isoputreanine |  | 2.64 [1.73-4.04] | < 0.001 | 0.002 | Amino Acid | Polyamine Metabolism |
| Alpha-ketoglutaramate | 0001552 | 2.57 [1.46-4.50] | 0.001 | 0.026 | Amino Acid | Glutamate Metabolism |
| Gamma-glutamylphenylalanine | 0000594 | 2.49 [1.40-4.42] | 0.002 | 0.031 | Peptide | Gamma-glutamyl Amino Acid |
| 1-palmitoyl-2-linoleoyl-gpe-16-0-18-2 | 0005322 | 2.37 [1.65-3.40] | < 0.001 | < 0.001 | Lipid | Phosphatidylethanolamine (PE) |
| 1-palmitoyl-2-oleoyl-gpe-16-0-18-1 | 0005320 | 2.31 [1.66-3.23] | < 0.001 | < 0.001 | Lipid | Phosphatidylethanolamine (PE) |
| Aspartate | 0000191 | 2.25 [1.40-3.61] | < 0.001 | 0.022 | Amino Acid | Alanine and Aspartate Metabolism |
| 1-palmitoyl-gpe-16-0 | 0011503 | 2.16 [1.30-3.58] | 0.003 | 0.039 | Lipid | Lysophospholipid |
| 1-stearoyl-2-arachidonoyl-gpe-18-0-20-4 | 0009003 | 2.10 [1.39-3.18] | < 0.001 | 0.018 | Lipid | Phosphatidylethanolamine (PE) |
| 1-palmitoyl-2-arachidonoyl-gpe-16-0-20-4 | 0005323 | 2.09 [1.43-3.04] | < 0.001 | 0.011 | Lipid | Phosphatidylethanolamine (PE) |
| 3-ureidopropionate | 0000026 | 1.99 [1.40-2.83] | < 0.001 | 0.011 | Nucleotide | Pyrimidine Metabolism, Uracil containing |
| N-formylanthranilic-acid | 0004089 | 1.97 [1.32-2.95] | 0.001 | 0.026 | Amino Acid | Tryptophan Metabolism |
| 1-stearoyl-2-linoleoyl-gpe-18-0-18-2 | 0008994 | 1.94 [1.37-2.74] | < 0.001 | 0.011 | Lipid | Phosphatidylethanolamine (PE) |
| Succinylcarnitine-c-4-dc | 0061717 | 1.92 [1.22-3.01] | 0.005 | 0.049 | Energy | TCA Cycle |
| Quinolinate | 0000232 | 1.89 [1.35-2.66] | < 0.001 | 0.011 | Cofactors and Vitamins | Nicotinate and Nicotinamide Metabolism |
| Gulonate | 0003290 | 1.89 [1.25-2.87] | 0.003 | 0.037 | Cofactors and Vitamins | Ascorbate and Aldarate Metabolism |
| Methionine-sulfone | 0062174 | 1.85 [1.26-2.70] | 0.002 | 0.031 | Amino Acid | Methionine, Cysteine, SAM and Taurine Metabolism |
| 1-carboxyethylphenylalanine |  | 1.81 [1.34-2.43] | < 0.001 | 0.011 | Amino Acid | Phenylalanine Metabolism |
| Glutamate | 0000148 | 1.80 [1.25-2.61] | 0.002 | 0.031 | Amino Acid | Glutamate Metabolism |
| 1-carboxyethyltyrosine |  | 1.77 [1.33-2.34] | < 0.001 | 0.011 | Amino Acid | Tyrosine Metabolism |
| 1-stearoyl-2-oleoyl-gpe-18-0-18-1 | 0008993 | 1.76 [1.30-2.37] | < 0.001 | 0.011 | Lipid | Phosphatidylethanolamine (PE) |
| Ylose | 0000098 | 1.75 [1.31-2.33] | < 0.001 | 0.011 | Carbohydrate | Pentose Metabolism |
| 1-palmitoyl-2-docosahexaenoyl-gpe-16-0-22-6 | 0008946 | 1.75 [1.27-2.40] | < 0.001 | 0.020 | Lipid | Phosphatidylethanolamine (PE) |
| 1-carboxyethylvaline |  | 1.69 [1.24-2.29] | < 0.001 | 0.022 | Amino Acid | Leucine, Isoleucine and Valine Metabolism |
| 1-stearoyl-2-docosahexaenoyl-gpe-18-0-22-6 | 0009012 | 1.67 [1.23-2.26] | 0.001 | 0.026 | Lipid | Phosphatidylethanolamine (PE) |
| 4-hydroxyhippurate | 0013678 | 1.64 [1.29-2.09] | < 0.001 | 0.011 | Xenobiotics | Benzoate Metabolism |
| 4-hydroxyglutamate | 0001344 | 1.64 [1.26-2.12] | < 0.001 | 0.011 | Amino Acid | Glutamate Metabolism |
| 1-carboxyethylleucine |  | 1.60 [1.18-2.18] | 0.003 | 0.037 | Amino Acid | Leucine, Isoleucine and Valine Metabolism |
| 1-oleoyl-2-docosahexaenoyl-gpe-18-1-22-6 | 0009078 | 1.57 [1.17-2.11] | 0.003 | 0.037 | Lipid | Phosphatidylethanolamine (PE) |
| 1-carboxyethylisoleucine |  | 1.56 [1.19-2.04] | 0.001 | 0.027 | Amino Acid | Leucine, Isoleucine and Valine Metabolism |
| 1-oleoyl-2-linoleoyl-gpe-18-1-18-2 | 0005349 | 1.54 [1.20-1.97] | < 0.001 | 0.021 | Lipid | Phosphatidylethanolamine (PE) |
| Fructose | 0000660 | 1.52 [1.15-2.02] | 0.003 | 0.043 | Carbohydrate | Fructose, Mannose and Galactose Metabolism |
| Orotidine | 0000788 | 1.49 [1.13-1.95] | 0.004 | 0.046 | Nucleotide | Pyrimidine Metabolism, Orotate containing |
| 3-indoxyl-sulfate | 0000682 | 1.45 [1.12-1.87] | 0.005 | 0.049 | Amino Acid | Tryptophan Metabolism |
| Phenol-sulfate | 0060015 | 1.42 [1.17-1.72] | < 0.001 | 0.018 | Amino Acid | Tyrosine Metabolism |
| 3-hydroxyhippurate | 0006116 | 1.31 [1.14-1.51] | < 0.001 | 0.011 | Xenobiotics | Benzoate Metabolism |
| 2-methoxyresorcinol-sulfate |  | 1.31 [1.11-1.55] | 0.002 | 0.031 | Xenobiotics | Chemical |
| 2-4-or-2-5-dimethylphenol-sulfate |  | 1.30 [1.09-1.54] | 0.004 | 0.044 | Xenobiotics | Food Component/Plant |
| Glycocholate | 0000138 | 1.27 [1.08-1.49] | 0.004 | 0.045 | Lipid | Primary Bile Acid Metabolism |
| Cinnamoylglycine | 0011621 | 0.83 [0.73-0.94] | 0.004 | 0.047 | Xenobiotics | Food Component/Plant |
| Palmitoleate-16-1-n-7 | 0003229 | 0.78 [0.66-0.92] | 0.004 | 0.044 | Lipid | Long Chain Monounsaturated Fatty Acid |
| Myristoleate-14-1-n-5 | 0002000 | 0.76 [0.63-0.92] | 0.005 | 0.049 | Lipid | Long Chain Monounsaturated Fatty Acid |
| Tetradecadienoate-14-2 | 0000560 | 0.76 [0.63-0.92] | 0.005 | 0.049 | Lipid | Long Chain Polyunsaturated Fatty Acid (n3 and n6) |
| 7-methylxanthine | 0001991 | 0.75 [0.62-0.90] | 0.003 | 0.037 | Xenobiotics | Xanthine Metabolism |
| 10-heptadecenoate-17-1-n-7 | 0060038 | 0.74 [0.61-0.90] | 0.003 | 0.041 | Lipid | Long Chain Monounsaturated Fatty Acid |
| Hexadecadienoate-16-2-n-6 | 0000477 | 0.74 [0.61-0.89] | 0.002 | 0.031 | Lipid | Long Chain Polyunsaturated Fatty Acid (n3 and n6) |
| Linolenate-alpha-or-gamma-18-3-n-3-or-6 | 0003073,  0001388 | 0.71 [0.57-0.88] | 0.002 | 0.031 | Lipid | Long Chain Polyunsaturated Fatty Acid (n3 and n6) |
| Oleate-vaccenate-18-1 | 0003231,  0000573,  0240219,  0000207 | 0.71 [0.56-0.90] | 0.005 | 0.049 | Lipid | Long Chain Monounsaturated Fatty Acid |
| Linoleate-18-2-n-6 | 0006270,  0000673 | 0.69 [0.54-0.87] | 0.002 | 0.033 | Lipid | Long Chain Polyunsaturated Fatty Acid (n3 and n6) |
| Octanoylcarnitine-c-8 | 0000791 | 0.68 [0.53-0.88] | 0.003 | 0.037 | Lipid | Fatty Acid Metabolism (Acyl Carnitine, Medium Chain) |
| Decanoylcarnitine-c-10 | 0000651 | 0.67 [0.52-0.87] | 0.002 | 0.034 | Lipid | Fatty Acid Metabolism (Acyl Carnitine, Medium Chain) |
| Dodecadienoate-12-2 |  | 0.66 [0.52-0.85] | 0.001 | 0.028 | Lipid | Fatty Acid, Dicarboxylate |
| 5-dodecenoylcarnitine-c-12-1 | 13326 | 0.64 [0.49-0.84] | 0.002 | 0.031 | Lipid | Fatty Acid Metabolism (Acyl Carnitine, Monounsaturated) |
| 10-undecenoate-11-1-n-1 | 0033724 | 0.63 [0.47-0.85] | 0.002 | 0.037 | Lipid | Medium Chain Fatty Acid |
| Myristoleoylcarnitine-c-14-1 | 0240588 | 0.63 [0.49-0.82] | < 0.001 | 0.018 | Lipid | Fatty Acid Metabolism (Acyl Carnitine, Monounsaturated) |
| Palmitoleoylcarnitine-c-16-1 | 0013207 | 0.62 [0.45-0.86] | 0.004 | 0.049 | Lipid | Fatty Acid Metabolism (Acyl Carnitine, Monounsaturated) |
| Cis-4-decenoylcarnitine-c-10-1 | 0013205 | 0.61 [0.44-0.84] | 0.003 | 0.037 | Lipid | Fatty Acid Metabolism (Acyl Carnitine, Monounsaturated) |
| Homostachydrine | 0033433 | 0.59 [0.43-0.81] | < 0.001 | 0.024 | Xenobiotics | Food Component/Plant |
| N-6-n-6-n-6-trimethyllysine | 0001325 | 0.55 [0.38-0.81] | 0.002 | 0.033 | Amino Acid | Lysine Metabolism |
| Glycerol | 0000131 | 0.53 [0.38-0.74] | < 0.001 | 0.011 | Lipid | Glycerolipid Metabolism |
| Stearoyl-ethanolamide | 0013078 | 0.50 [0.31-0.80] | 0.004 | 0.045 | Lipid | Endocannabinoid |
| Oleoylcarnitine-c-18-1 | 0005065 | 0.49 [0.30-0.80] | 0.005 | 0.049 | Lipid | Fatty Acid Metabolism (Acyl Carnitine, Monounsaturated) |
| Eicosenoylcarnitine-c-20-1 |  | 0.47 [0.31-0.70] | < 0.001 | 0.011 | Lipid | Fatty Acid Metabolism (Acyl Carnitine, Monounsaturated) |
| Sphingomyelin-d-18-2-18-1 | 0001348 | 0.43 [0.26-0.69] | < 0.001 | 0.020 | Lipid | Sphingomyelins |
| Citrulline | 0000904 | 0.41 [0.22-0.74] | 0.003 | 0.043 | Amino Acid | Urea cycle; Arginine and Proline Metabolism |
| Stearoyl-sphingomyelin-d-18-1-18-0 | 0001348 | 0.33 [0.16-0.68] | 0.003 | 0.038 | Lipid | Sphingomyelins |
| Thyroxine | 0000248 | 0.32 [0.16-0.64] | 0.001 | 0.028 | Amino Acid | Tyrosine Metabolism |
| Sphingomyelin-d-18-1-18-1-d-18-2-18-0 | 0012101 | 0.32 [0.16-0.66] | 0.002 | 0.031 | Lipid | Sphingomyelins |
| Sphingomyelin-d-18-1-20-1-d-18-2-20-0 | 0240610,  0240632 | 0.31 [0.16-0.63] | 0.001 | 0.026 | Lipid | Sphingomyelins |

Adjusted for age, sex, body mass index, smoking status, pack-years, and inhaled corticosteroid use

**REFERENCES FOR Additional file 1**

1. Regan EA, Hokanson JE, Murphy JR, Make B, Lynch DA, Beaty TH, et al. Genetic epidemiology of COPD (COPDGene) study design. COPD 2010;7:32-43.

2. Ash SY, Harmouche R, Ross JC, Diaz AA, Hunninghake GM, Putman RK, et al. The Objective Identification and Quantification of Interstitial Lung Abnormalities in Smokers. Acad Radiol 2017;24:941-946.

3. Diaz AA, Strand M, Coxson HO, Ross JC, San Jose Estepar R, Lynch D, et al. Disease Severity Dependence of the Longitudinal Association Between CT Lung Density and Lung Function in Smokers. Chest 2018;153:638-645.

4. Gillenwater LA, Pratte KA, Hobbs BD, Cho MH, Zhuang Y, Halper-Stromberg E, et al. Plasma Metabolomic Signatures of Chronic Obstructive Pulmonary Disease and the Impact of Genetic Variants on Phenotype-Driven Modules. Netw Syst Med 2020;3:159-181.

5. Evans AM, DeHaven CD, Barrett T, Mitchell M, Milgram E. Integrated, nontargeted ultrahigh performance liquid chromatography/electrospray ionization tandem mass spectrometry platform for the identification and relative quantification of the small-molecule complement of biological systems. Anal Chem 2009;81:6656-67.

6. Dehaven CD, Evans AM, Dai H, Lawton KA. Organization of GC/MS and LC/MS metabolomics data into chemical libraries. J Cheminform 2010;2:9.

7. Miller MJ, Kennedy AD, Eckhart AD, Burrage LC, Wulff JE, Miller LA, et al. Untargeted metabolomic analysis for the clinical screening of inborn errors of metabolism. J Inherit Metab Dis 2015;38:1029-39.

8. Lippi G, Blanckaert N, Bonini P, Green S, Kitchen S, Palicka V, et al. Haemolysis: an overview of the leading cause of unsuitable specimens in clinical laboratories. Clin Chem Lab Med 2008;46:764-72.

9. Stringer KA, Younger JG, McHugh C, Yeomans L, Finkel MA, Puskarich MA, et al. Whole Blood Reveals More Metabolic Detail of the Human Metabolome than Serum as Measured by 1H-NMR Spectroscopy: Implications for Sepsis Metabolomics. Shock 2015;44:200-8.

10. Townsend MK, Clish CB, Kraft P, Wu C, Souza AL, Deik AA, et al. Reproducibility of metabolomic profiles among men and women in 2 large cohort studies. Clin Chem 2013;59:1657-67.
